# Traditional Tibetan medicine: therapeutic potential in lung diseases

**DOI:** 10.3389/fphar.2024.1365911

**Published:** 2024-03-18

**Authors:** Canlin Li, Yuan Li, Xi Huang, Si Li, Kangzhuo Sangji, Rui Gu

**Affiliations:** ^1^ School of Ethnic Medicine, Chengdu University of Traditional Chinese Medicine, Chengdu, China; ^2^ School of Pharmacy, Chengdu University of Traditional Chinese Medicine, Chengdu, China

**Keywords:** traditional Tibetan medicine, natural medicine, lung diseases, pharmacological properties, bioactive components

## Abstract

Lung diseases have become a major threat to human health worldwide. Despite advances in treatment and intervention in recent years, effective drugs are still lacking for many lung diseases. As a traditional natural medicine, Tibetan medicine has had a long history of medicinal use in ethnic minority areas, and from ancient times to the present, it has a good effect on the treatment of lung diseases and has attracted more and more attention. In this review, a total of 586 Tibetan medicines were compiled through literature research of 25 classical works on Tibetan medicine, drug standards, and some Chinese and English databases. Among them, 33 Tibetan medicines have been studied to show their effectiveness in treating lung diseases. To investigate the uses of these Tibetan medicines in greater depth, we have reviewed the ethnomedicinal, phytochemical and pharmacological properties of the four commonly used Tibetan medicines for lung diseases (rhodiola, gentian, sea buckthorn, liexiang dujuan) and the five most frequently used Tibetan medicines (safflower, licorice, sandalwood, costus, myrobalan). It is expected to provide some reference for the development of new drugs of lung diseases in the future.

## 1 Introduction

Due to factors such as environmental pollution, smoking, economic development, an accelerated pace of life, and poor lifestyle, the incidence of lung diseases has increased year by year in recent years, and lung diseases have become an important disease threatening human health worldwide (Xiao and Wei, 2018) and is a major cause of death and disability worldwide (Collaborators, 2020). Despite recent advances in patient care and intervention, many lung diseases still lack effective treatments and remain incurable (Ryter et al., 2012). Chronic obstructive pulmonary disease, asthma, tuberculosis, interstitial lung diseases, lung cancer, bronchiectasis, lung abscess, and pneumothorax are the most common lung diseases at present (Xiao and Zheng, 2018). Among them, three respiratory diseases such as chronic obstructive pulmonary disease (COPD), lung cancer, and asthma have been among the top 10 causes of death worldwide (Levine and Marciniuk, 2022). In recent years, several types of lung diseases have received widespread attention and have become a public health problem. The latest data from the World Health Organization on mortality and causes of death showed that the prevalence of COPD will continue to rise over the next 40 years as smoking rates rise in developing countries and populations in high-income countries age, with projections of more than 5.4 million deaths/year from COPD and related diseases by 2060 (Geng and Wang, 2021). In 2020, there were 2.21 million new cases and 1.8 million deaths due to lung cancer worldwide with age-standardized incidence rate (ASIR) of 22.4/100,000 (male: 31.5; female: 14.6) and age-standardized mortality rates (ASMR) of 18.0/100,000, (male: 25.9; female: 11.2/100,000). As the projections, there will be 3.8 million incident cases and 3.2 million deaths globally due to lung cancer in 2050 (Sharma, 2022). Interstitial lung disease (ILD) is a group of heterogeneous lung diseases that includes more than 200 parenchymal lesions characterized by extensive fibrosis or inflammatory abnormalities of the lung parenchyma (Ryerson and Collard, 2013). Statistically, up to one-third of patients with ILD develop severe pulmonary fibrosis and are usually diagnosed 2–9 months after the onset of symptoms (Wijsenbeek et al., 2019). Besides, pneumonia is a major health problem worldwide due to its high incidence and susceptibility to complications and high mortality rate among infectious diseases. In particular, the global pandemic of the novel coronavirus pneumonia (COVID-19) that broke out at the end of 2019 has caused unassailable damage to human society in terms of life and health. As of 4 February 2024, more than 775 million confirmed cases of COVID-19 and more than 7.0 million deaths have been reported globally (https://covid19.who.int/). Available studies show that neo-coronavirus infection may cause non-negligible long-term health damage (Chen et al., 2023).

Many of the available therapies for lung diseases have limited effectiveness or are associated with side effects (Kaczynska et al., 2023). Clofazimine, Amikacin, Tigecycline, and Linezolid are currently used to treat *Mycobacterium* abscessus complex-lung disease. However, these drugs have varying degrees of side effects, including gastrointestinal disturbances, reddish-brown skin discoloration, ototoxicity, hemoptysis, nephrotoxicity, and peripheral neuropathy (Weng et al., 2020). Although, in recent years, new approaches and new drugs have emerged for the treatment of lung diseases, such as biologic therapies, and Lactoferrin (LF). The safety and efficacy are still remaining somewhat controversial (Scallan et al., 2020; Kaczynska et al., 2023). For example, despite the effectiveness of TNF-α inhibitors in improving clinical status and slowing joint disease progression, pulmonary toxicity remains a major concern (Dixon et al., 2010; Komiya et al., 2011; Perez-Alvarez et al., 2011; Jani et al., 2014; Perez-De-Lis et al., 2017). In animal studies, LF appears to be an excellent drug, but in human studies, it is more of an immunomodulator with limited therapeutic potential for lung diseases (Kaczynska et al., 2023).

At present, there are scholars have sorted out Tibetan medicine prescriptions for COVID-19, providing important information for the basic research, clinical application, and drug development of traditional medicines for the treatment of COVID-19 or other infectious diseases (Zhang et al., 2023). Biological activities of Gentianeae in Tibetan Medicine (Chi et al., 2021) and Tibetan medicine Bang Jiang (Li et al., 2023) have been compiled. And presented their protective effects on the respiratory system. However, the above researches only provide a detailed introduction to a certain type of lung disease or Tibetan medicine of the Gentiana genus, and do not provide a comprehensive summary of the treatment of lung diseases with Tibetan medicine. Therefore, this study provides the first available data compilation for the ethnic medical knowledge of Tibetan medicine for the treatment of lung diseases. In this paper, based on the literature survey of Tibetan medicine monographs and drug standards, the Tibetan medicine, and Tibetan prescription used in the traditional Tibetan medical system, here, we summarise the methods indicated for lung diseases. Five hundred and eighty-six vegetal (medicinal) species were found to treat lung diseases in the traditional Tibetan medicine system. Among them, Rhodiola, Gentian, Sea buckthorn, and Liexiang dujuan are the ones that the most frequently used in Tibetan hospitals. Among the Tibetan medicine prescriptions recorded in the literature, safflower, licorice, sandalwood, sandalwood, and mirobaran are used most frequently. Therefore, this article provides a detailed introduction to the above drugs, including traditional usage, metabolites, pharmacological effects, and clinical research.

Traditional Tibetan medicine (TTM) is rich in content and complete in theory, and its achievements in physiology, pathology, therapeutics, and pharmacology have not only made great contributions to Chinese medicine but have also long attracted the attention of world medicine. On the other hand, many of the therapies nowadays for lung diseases have limited effectiveness or are associated with side effects. As a result, it is hoped that this review can provide a reference for the development of new drugs with no side effects and significant therapeutic effects for the treatment of lung diseases, thereby improving the quality of life of patients and prolonging their survival time.

## 2 Methods

We manually searched 25 Tibetan herbal monographs and drug standards, including Jing Zhu Materia Medica, Chinese Dictionary of Ethnic Medicines, Tibetan Medicine Drug Standards, Prescription Medicines for Ethnic Medicine Formulations, Diqing Tibetan Medicine, and National Tibetan Medicine Standard Complete Book, to collect information on Tibetan herbal medicines and prescriptions for the treatment of lung diseases. The data collected from this literature included names, original species, families, medicinal parts, and therapeutic diseases. The botanical names of original plants are mainly from the references, and verified through the “Flora of China (http://frps.eflora.cn/)” database based on their Chinese names. The database of “The Plant List (http://www.theplantlist.org/)” is used to standardize their Latin names. To understand the most commonly used Tibetan medicines for lung diseases, data mining of Tibetan prescriptions was carried out using Traditional Chinese Medicine Inheritance Support System (TCMISS) (Version 2.5) software. All the collected prescriptions were entered into the TCMISS software, and the frequency of use of each drug was ranked from the largest to the smallest by clicking on the “Frequency of Use Statistics” module. In addition, we obtained the active ingredients and biological/pharmacological effects of the selected species through Chinese databases (e.g., Wanfang, Weipu, China Knowledge Network) and international databases (e.g., ISI Web of Science, MEDLINE, Science Direct, Google Scholar) using their Chinese names, English or Latin names as search terms.

## 3 Results

### 3.1 Understanding the pathogeny of lung disease in TTM theory

TTM is one of the oldest known medical systems in the world. It has a long history of more than 2,000 years. TTM originated from a local folk tradition called “Bon”, which dates back to 300 B.C. It is a fusion of theories from early Chinese medicine, Indian medicine (Ayurveda), and Arabic medicine, and has gradually developed into a unique system of medicine (Li et al., 2018a). The Tibetan medical system is very broad in content. Its basic theories include the doctrine of the three factors, the doctrine of the five sources, the five elements, and physiology, pathology, diagnosis, treatment principles, and treatment methods. Among them, the theory of three factors is the core of Tibetan medicine theory, which explains the physiological activities, pathogenesis, and treatment mechanism of the human body by the properties of three substances, namely, “Loong (རླུང་།།)”, “Tripa (མཁྲིས་པ།)”, “Baekan (བད་ཀན།)”. It believes that these three substances are the material basis of the human body and the energy of life activities. In the physiological state, the three substances maintain coordination and balance to keep the normal physiological activities of the human body; in the pathological state, the three are out of balance under the influence of various substances, leading to various diseases.

**FIGURE 1:**
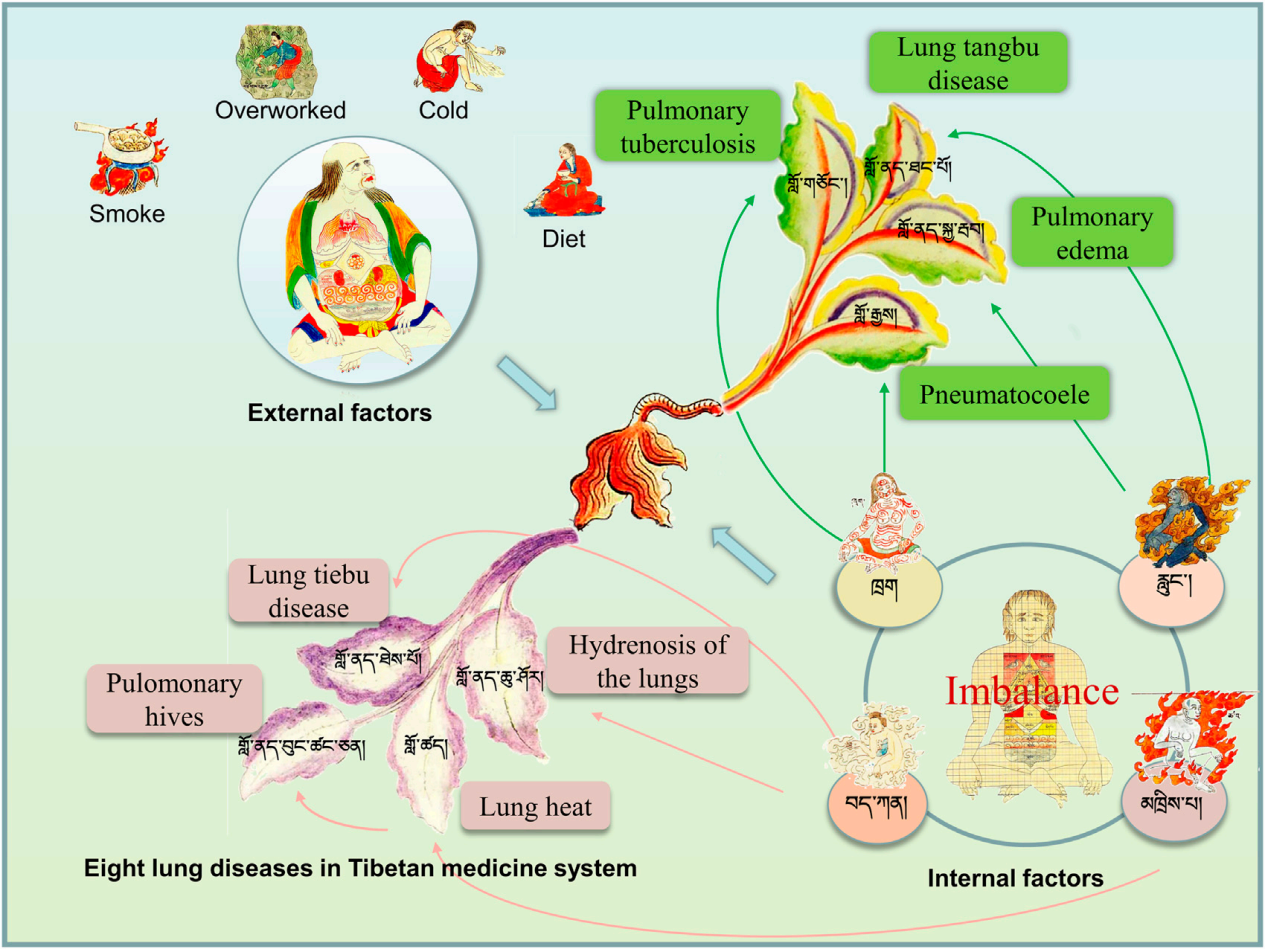
Understanding of pulmonary diseases in traditional Tibetan medicine system. Lung diseases are caused internally by the imbalance of the body’s “Loong (རླུང་།།)”, “Tripa (མཁྲིས་པ།)”, “Baekan (བད་ཀན།)”, and “Blood (ཁྲག)” and foutr external causes.

According to Tibetan medicine, the lung is a ministerial organ, the second most important of the five organs. It is written in the Secret Department of “Si Bu Yi Dian (The Four Medical Tantras)” that lung diseases are caused internally by the imbalance of the body’s “Loong (རླུང་།།)”, “Tripa (མཁྲིས་པ།)”, “Baekan (བད་ཀན།)”, and “Blood (ཁྲག)”. External causes include invasion of bodily fluids into the lungs, eating rotten and acidic food, old ghee by the barrel, overly salty food, smoking, long-term colds, and overexertion. In Tibetan medicine, lung diseases are divided into lung “tangbu” disease (གློ་ནད་ཐང་པོ།) and pulmonary edema (གློ་ནད་སྐྱ་རྦབ།) caused by “Loong (རླུང་།།)”, lung heat (གློ་ཚད།)caused by “Tripa (མཁྲིས་པ།)”, lung hydropathy and lung “tiebu” disease (གློ་ནད་ཐེས་པོ།) caused by“Baekan (བད་ཀན།)”, pulmonary tuberculosis (གློ་གཅོང་།) and pneumatocoele (གློ་རྒྱས།) caused by “Blood (ཁྲག)”, and pulmonary honeycomb (གློ་ནད་བུང་ཚང་ཅན།) caused by lung heat for a long time (Figure 1). The lungs are where “Baekan (བད་ཀན།)" exists, and the condition is milder in the summer and more severe in the winter, more comfortable during the day and more severe at night.

Most of the lung diseases recorded in “Jing Zhu Materia Medica” are lung fever diseases. In Tibetan medicine, lung fever is caused by external wind or excessive inhalation of smoke and dust, or excessive consumption of spicy and thick flavors, resulting in an imbalance of the three major substances (Loong, Tripa, Baekan) of the body, which turn into three evils and offend the lung, and then turn into heat in the lung, or excessive internal heat. Furthermore, because of the wind and cold, the hair orifices are closed and the fire and heat cannot escape through the hair orifices, resulting in the heat becoming more and more intense and burning the lung qi, causing the disease (Yang, 2013). Tibetan medicine has a unique understanding and insight into modern lung diseases. According to TTM theory, as the body is disturbed by external factors such as season, diet and living, the three factors in the body, Loong, Tripa, Baekan becomes out of balance, and the function becomes biased, which causes the COVID-19 (Liu et al., 2020). Chronic obstructive pulmonary disease is called pulmonary “tiebu” disease (གློ་ནད་ཐེས་པོ།) in Tibetan medicine system, which is a kind of lung disease caused by the invasion of “Baekan (བད་ཀན།)” into the lung. The symptoms include vomiting a large amount of green turbid sputum, shortness of breath, heaviness and fullness of the upper body, and a little peace after the sputum is released (Ren and Li, 2015).

### 3.2 Collation and analysis of natural Tibetan medicines

**FIGURE 2:**
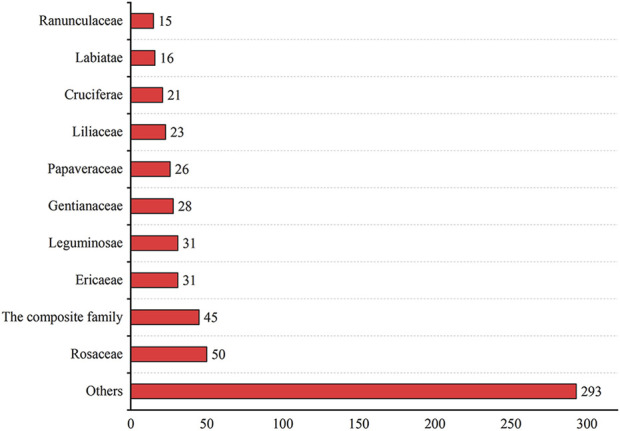
The most common families and genera of Tibetan medicine in the treatment of pulmonary diseases.

**FIGURE 3:**
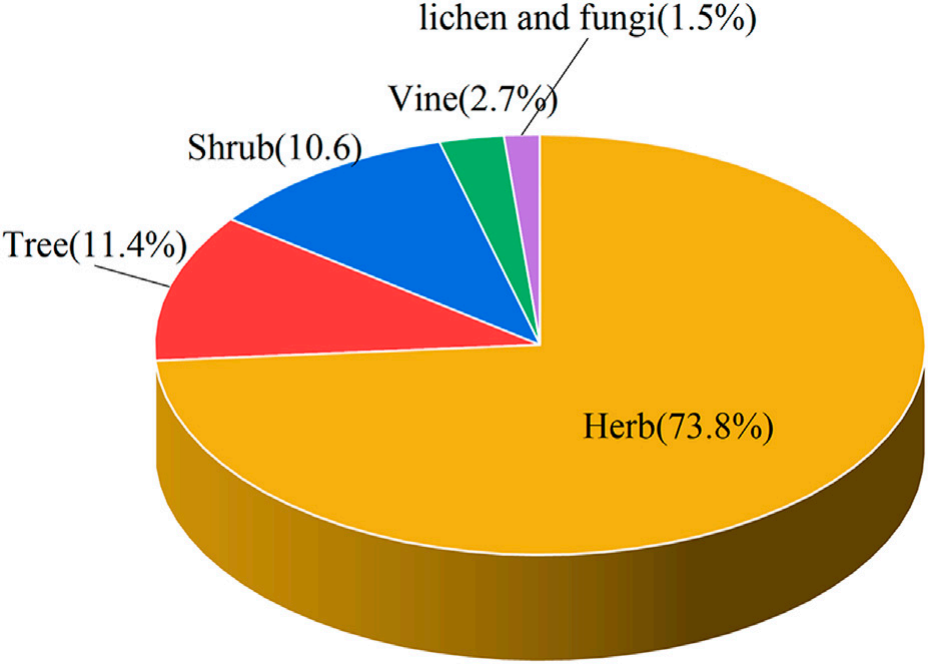
The most common source of Tibetan medicine for pulmonary diseases.

**FIGURE 4:**
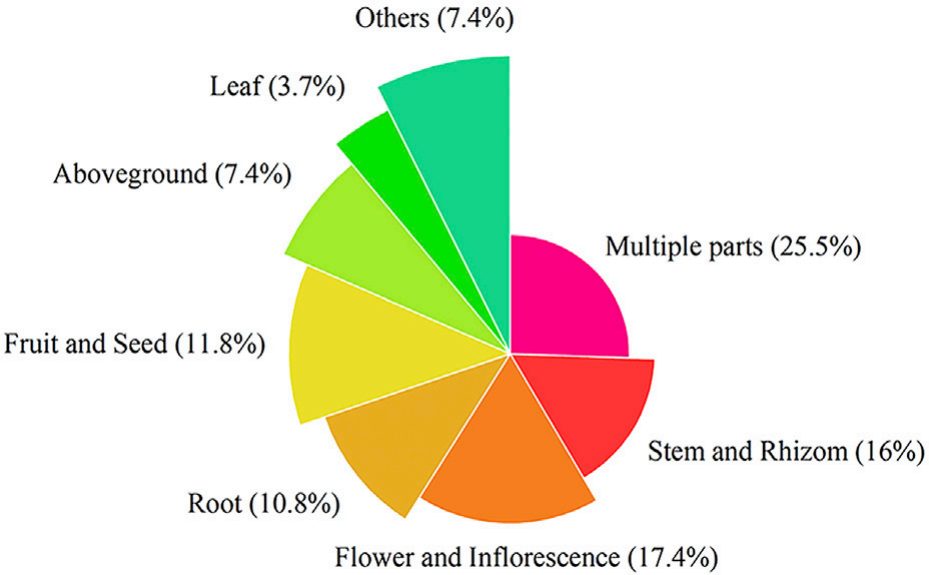
The most common vegetal products used of Tibetan medicine for pulmonary diseases.

This paper recorded the use of 586 natural Botanical Tibetan medicines in the traditional system of Tibetan medicine for the treatment of various lung diseases [see Supplementary Material]. The scientific names, families, medicinal parts, diseases treated, and reported biological activities of some natural medicines are given in Table 1 and Table 2. These medicines are distributed in 77 families. The most common families were: Rosaceae (8.6%), Composite (7.7%), Ericaceae (5.3%), Leguminosae (5.3%), Gentianaceae (4.8%), Papaveraceae (4.4%), Liliaceae (3.9%), Cruciferae (3.6%), Labiatae (2.7%), Ranunculaceae (2.6%) (Figure 2). Moreover, herbs (73.7%) were the main source of these natural Tibetan medicines, followed by trees and shrubs (11.4%, 10.6%), animals (4.8%), vines 2.7%, lichens and fungi (1.0%, 0.5%) (Figure 3). Among the various vegetal products, the whole plant was used most frequently (30.1%), followed by 17.8% for multipart, 11.1% for stems (including herbaceous stems, rhizomes, tubers, bulbs, *etc.*), flowers and roots (10.6%, 7.5%), fruits and above-ground parts (6.5%, 5.1%), leaves (2.6%), seeds and inflorescences (1.7%, 1.5%) (Figure 4).

**TABLE 1:** The most frequently medcines for treating pulmonary diseases in traditonal Tibetan medical system.

No.	Latin name	Tibetan name	Family	Types of plants	Vegetal products	Treated pulmonary diseases
1	*Rhodiola crenulata* (Hook. f. et Thoms.) H. Ohba	སྲོ་མོ་དམར་པོ།	Crassulaceae	Herb	Rhizome, root, whole herb	Lung disease, lung-heat cough Jia and Zhang. (2016)
2	*Gentiana veitchiorum* Hemsl	སྦལ་ལ་ནག་པོ།	Gentianaceae	Herb	Flower, root, rhizome	Lung heat, cough with lung heat Herbalism. (2002)
3	*Hippophae rhamnoides* L	སྟར་བུ།	Elaeagnaceae	Shrub	Dried ripe fruit or decoction and paste of fruit	Tracheitis Gema. (2016)
	*Hippophae. thibetana* Schlechtend					
	*Hippophae rhamnoides* subsp. *Sinensis* Rousi					
	*Hippophae neurocarpa* S. W. Liu et T. N. He					
4	*Rhododendron anthopogonoides* Maxim	ད་ལིས།	Ericaceae	Shrub	Dried flower, leaf	Tracheitis, emphysema China. (1995)
	*R. cephalanthum* Franch					
	*R. primulaeflarum* Bur. Er Franch					
5	*Carthamus tinctorius* L	སྦལ་ལ་ནག་པོ།	Comopsite	Herb	Flower	Pneumonia, pulmonary tuberculosis Herbalism. (2002)
6	*Aucklandia lappa* Decne	སྦལ་ལ་ནག་པོ།	Comopsite	Herb	Dried root	Pneumonia Herbalism. (2002)
7	*Arctium lappa* L	བྱི་བཟུང་།	Comopsite	Herb	Dried ripe fruit	Wind-heat type common cold Zhong and Song. (2020)
8	*Artemisia scoparia* Waldst. Et Kit	ཚར་བོང་།	Comopsite	Herb	Whole plant	Lung heat Control. (1996)
9	*Aster tataricus* L. f	ལུག་མིག	Comopsite	herb	Flower Sequence	Influenza Jia and Zhang. (2016)
10	*Glycyrrhiza uralensis* Fisch	སྦལ་ལ་ནག་པོ།	Leguminosae	Herb	Dried roots and rhizomes	Lung disease Dimaer. (2012)
11	*Santalum album* L	སྦལ་ལ་ནག་པོ།	Santalaceae	Tree	Heartwood	Lung heat, pneumonia, lung abscess Yang. (1987)
12	*Terminalia chebula* Retz	སྦལ་ལ་ནག་པོ།	Combretaceae	Tree	Dried and ripe fruit	Astringing lun for relieving cough Zhong and Song. (2020)
	*Terminalia chebula* Retz var. *tomentella* Kurz					
13	*Ephedra saxatilis* Royle ex Florin	སྦལ་ལ་ནག་པོ།	Ephedraceae	Herb	Dried grassstem	Bronchial asthma Zhong and Song. (2020)
14	*Schisandra sphenanthera* Rehd. et Wils	སྦལ་ལ་ནག་པོ།	Magnoliaceae	Vine	Dried ripe fruit	Maintenance of lungs Zhong and Song. (2020)
15	*Houttuynia* cordata Thunb	སྦལ་ལ་ནག་པོ།	Saururaceae	Herb	Fresh whole grass or dried ground parts	Abscess of lung, pneumonia, pulmonary tuberculosis Luo. (1997)
16	*Lithospermum erythrorhizon* Sieb. et Zucc	འབྲི་མོག	Boraginaceae	Herb	Dried root	Abscess of lung, cough with lung heat Ma. (2012)
17	*Oxytropis falcata* Bunge	སྔོ་སྟག་ཤ།	Leguminosae	Herb	Dried flower	Lung heat Control. (1996)
18	*Delphinium trichophorum* Franch	ག་བུར་ཏིས་ལོ།	Ranunculaceae	Herb	Aboveground part	Pandemic fever, cough with lung heat Jia and Zhang. (2016)
19	*Hypecoum erectum* L	པར་པ་ཏ།	Papaveraceae	Herb	Whole palnt	Cough from pneumonia Editorial Committee of Tibetan Medicine Journal Chinese Academy of Sciences. (1991)
20	*Meconopsis punicea* Maxim	ཨུཏྤལ་དམར་པོ།	Papaveraceae	Herb	Whole palnt	Pneumonia, lung heat Luo. (1997)
21	*Foeniculum vulgare* Mill	ཟི་ར་དཀར་པོ།	Umbelliferae	Herb	Fwhole plant	Lung heat, pneumonia, pulmonary tuberculosis Yang. (1987)
22	*Asparagus cochinchinensis* (Lour.) Merr	ལུག་མིག	Liliaceae	Herb	Tuber	Atrophic lung disease, abscess of lung Jia and Zhang. (2016)
23	*Fritillaria cirrhosa* D. Don;*Fritillaria unibracteata* Hsiao et K. C. Hsia	ཨ་བི་ཥ།	Liliaceae	Herb	Dried bulb	Tracheitis, cough with lung heat Jia. (2005)
	*Fritillaria przewalskii* Maxim					
	*Fritillaria delavayi* Franch					
24	*Lycium barbarum* L	འཕང་སྐྱ།	Solanaceae	Tree	Velamen	Pulmonary tuberculosis Jia and Zhang. (2016)
25	*Vitex trifolia* L	_	Verbenaceae	Shrub	Fruit	Pulmonary crests, Pneumonia Jia and Zhang. (2016)
26	*Fagopyrum dibotrys* (D. Don) Hara	བྲ་བོ།	Polygonaceae	Herb	Dried rhizome	Lung cancer Jia. (2005)
27	*Geranium wilfordii* Maxim	གླ་སྒང་།	Geraniaceae	Herb	Aboveground parts	Lung disease Zhong and Song. (2020)
28	*Gymnadenia conopsea* (L.)R. Br	དབང་ལག	Orchidaceae	Herb	Dried tuber	Lung disease, pulmonary cough and wheeze (Wang. (2004)
29	*Tinospora cordifolia* Miers	སླེ་ཏྲེས།	Menispermaceae	Vine	Rattan	Lung disease Wang. (2004)
30	*Vitis amurensis* Rupr	སླེ་ཏྲེས།	Vitaceae	Vine	Fruit	Pneumonia, cough of pulmonary furuncles, lung heat, lung disease Jia and Zhang. (2016)
31	*Usnea longissima* Ach	སླེ་ཏྲེས།	Usneaceae		Dried thallus	Lung heat Zhong and Song. (2020)
	*Usnea diffracta* Vain					
32	*Tremella fuciformis* berk	སླེ་ཏྲེས།	Tremellaceae		Subentity	Cough due to deficiency of the lung; dry pulmonaray cough; atrophic lung disease Luo. (1997)
33	*Cordyceps sinensis* (Berk.) Sacc	སླེ་ཏྲེས།			The fungus of the ergot family Cordyceps sinensis parasitizes on the dried complex of the larval larvae and carcasses of the Batmoth family insects	Lung disease, bronchitis; pulmonary tuberculosis, pulmonary tuberculosis Jia and Zhang. (2016)

**TABLE 2:** Biological activities of medcines in the traditonal Tibetan medical system.

NO.	Name	Biological activities	Bioactive components
1	*Rhodiola crenulata* (Hook. f. et Thoms.) H. Ohba	Protecting against lung injury in COPD mice Carozzi et al. (2017); Zhang et al. (2019), Anti-lung cancer Zhang et al. (2013a); Huang et al. (2023), acute lung injury Liu et al. (2017a); Huang et al. (2017), protective effect of hyperoxia-induced lung injury Zhu et al. (2021a), protective effect against intermittent hypoxia (IH)-induced lung injury Wu et al. (2019), protective effect on lung tissue damage in rats with acute plateau advancement Wang et al. (2022c), improves the inflammatory response of lung tissue in rats with severe pneumonia Wang et al. (2021a), inhibits emphysema development in mice Tang. (2022)	Salidroside
2	*Gentiana veitchiorum* Hemsl	Inhibiting the migration of lung cancer A-549 cells (Chen et al. (2018), therapeutic effects on pulmonary fibrosis (Zheng. (2010), anti-pulmonary fibrosis Shi et al. (2016)	Gentiopicroside
3	*Hippophae rhamnoides* L., *Hippophae. thibetana* Schlechtend, *Hippophae rhamnoides* subsp. *Sinensis* Rousi, *Hippophae neurocarpa* S. W. Liu et T. N. He	Inhibition of proliferation of A549 cell line Jia et al. (2020), and treatment of chronic bronchitis Ren. (2019)	Total flavonoids of sea buckthorn
4	*Rhododendron anthopogonoides* Maxim., *R. cephalanthum* Franch., *R. primulaeflarum* Bur. Er Franch	Inhibitory effect on the growth of Stretococcus pneumoniae College. (1973a), cough suppressing, expectorant and asthma calming effects (College. (1973b); Hu. (2015)	Hyperoside, 4-phenyl-2-butanone
5	*Carthamus tinctorius* L	Anti-pulmonary fibrosis Luan et al. (2019); Luan et al. (2021), anti-pulmonary fibrosis and lung cancer Qiao et al. (2020), improvement of lung function in COPD rats Bao et al. (2021), anti-acute lung injury rats (Wang. (2020)	SYA, safflower yellow, HSYA, AHSYB
6	*Aucklandia lappa* Decne	Anti-lung cancer Hao et al. (2010); Qiao et al. (2020)	Parthenolide, dehydrocostus lactone, costunolide
7	*Arctium lappa* L	Inhibiting neuronal apoptosis in rats with pneumococcal meningitis (Leng and Li. (2022), anti-lung cancer (Miao et al. (2021), inhibiting inflammation in lung tissue and growth of H460 lung cancer cells Yang et al. (2005); Wang. (2014); Kang et al. (2017), preventing and treating acute lung injury Zhang et al. (2010), anti-lung cancer activity Takasaki et al. (2000)	ATG, chlorogenic acid
8	*Artemisia scoparia* Waldst. Et Kit	Protective effects on human lung epithelial cell A549 induced by RSV Ma et al. (2021), treating acute lung injury (Wang et al. (2018)	ASTF
9	*Aster tataricus* L. f	Alleviating the pathological damage caused by *Mycoplasma* pneumoniae infection in mouse lung tissue Wang et al. (2022d), improving the inflammation of lung tissue in young SD rats with asthma Ai and Li. (2022), inhibiting the proliferation and invasion of lung cancer cells Yao et al. (2022)	Asterone
10	*Glycyrrhiza uralensis* Fisch	Anti-cough and expectorant, improvement of lung function Wang et al. (2021a), anti-pneumonia Xie et al. (2009); Xie and Guan. (2009), anti-acute lung injury in mice Furusawa et al. (2009); Chu et al. (2012); Ren et al. (2016), inhibiting apoptosis Tang et al. (2007); Wang et al. (2012), protective effect against acute lung injury Li. (2018b); Chen. (2019), inhibiting collagen fiber synthesis and fibroblast proliferation (He et al. (2006); He et al. (2008); He et al. (2009), anti-pulmonary fibrosis Ye et al. (2007), anti-acute radiation lung injury Yang. (2014), anti-pulmonary fibrosis Li. (2018a), reducing chronic lung injury Bu et al. (2010), inducing apoptosis in non-small cell lung cancer cells Oh et al. (2019)	Licochalcone B, liquiritigenin, isoliquiritin, liquiritin, glycyrrhizin, monoammonium glycyrrhizinate, glycyrrhetic acid, diammonium glycyrrhizinate, Licochalcone A
11	*Santalum album* L	Toxic effects on A549 human lung adenocarcinoma cells MATSUO and MIMAKI. (2020)	(7R,8R)-5-O-demethylbilagrewin, bilagrewin
12	*Terminalia chebula* Retz., *Terminalia chebula* Retz var. *tomentella* Kurz	Anti-actinomyces pneumoniae Kang et al. (2014), inhibition of lung cancer A549 cell proliferation Lin et al. (2011); Bao et al. (2012); Tian et al. (2015); Ravi et al. (2016)	
13	*Ephedra saxatilis* Royle ex Florin	Treating lung tissue damage caused by influenza A virus Geng. (2019); Deng et al. (2020), alleviating lung injury and inflammatory reaction caused by viral infection Xiaoting et al. (2020), anti-lung cancer Shao et al. (2021), reducing lung coefficient in rats with pulmonary fibrosis Gao. (2010)	Pseudoephedrine, ephedrine, ESP-B4
14	*Schisandra sphenanthera* Rehd. et Wils	Inhibiting the process of pulmonary fibrosis Wei et al. (2017), inhibiting the growth of pneumonia strain (CV6) Hakala et al. (2015), reducing inflammatory damage in lung tissue Sun et al. (2021), treating acute lung inflammation Bae et al. (2012)	Schisandrin B, schisandra chinensis lignans
15	*Houttuynia* cordata Thunb	Anti-pulmonary fibrosis (PF) Zhu et al. (2021b), alleviating lung inflammatory injury Xu et al. (2015)	Polysaccharides
16	*Lithospermum erythrorhizon* Sieb. et Zucc	Radiosensitization effect on Lewis lung cancer Luo et al. (2004)	Shikonin
17	*Oxytropis falcata* Bunge	Inhibitory effect on idiopathic pulmonary fibrosis (Zhang et al. (2022a)	TFOFB
18	*Delphinium trichophorum* Franch	Alleviating pulmonary fibrosis Arunachalam et al. (2022)	Diterpenoid alkaloids
19	*Hypecoum erectum* L	Alleviating structural changes in lung tissue Zhang et al. (2016b), anti-SARS-CoV-2 effects Zhong et al. (2021)	Hypecorinine, hyperectine, leptocarpinine
20	*Meconopsis punicea* Maxim	95% ethanol extract from it can significantly improve lung injury Huang. (2016)	-
21	*Foeniculum vulgare* Mill	Protecting against acute Lung Injury Lee et al. (2015)	-
22	*Asparagus cochinchinensis* (Lour.) Merr	Inhibitory effect on Lewis lung cancer Li et al. (2000)	ACP
23	*Fritillaria cirrhosa* D. Don;*Fritillaria unibracteata* Hsiao et K. C. Hsia, *Fritillaria przewalskii* Maxim., *Fritillaria delavayi* Franch	Inhibitory effect on A549 cell Liu et al. (2023)	-
24	*Lycium barbarum* L	Inhibiting pulmonary inflammatory response Li et al. (2018), regulating the expression of apoptosis related proteins and proto-oncogenes in human embryonic lung fibroblasts induced by beryllium sulfate Wang et al. (2017), inhibiting the development of pulmonary fibrosis Liu et al. (2016), inhibiting the proliferation of human lung adenocarcinoma cells (A549) Wang et al. (2016)	LBP, lyciyoui-sides
25	*Vitex trifolia* L	Inhibiting the proliferation and promoting apoptosis of human NSCLC line H322 Li et al. (2020b), inhibiting the growth and inducing apoptosis of human lung cancer A549 cells Hu. (2015), inhibiting human small cell lung cancer NCI-H446 cell line lung cancer stem cells Cao. (2014), inhibiting lung cancer Lu1 cells D ıaz et al. (2003)	Vitexicarpin, FVTF, polymethoxy flavones
26	*Fagopyrum dibotrys* (D. Don) Hara	Anti-pneumonia effect Liu. (2023), anti-mycoplasma pneumoniae Liu. (2022)	Luteolin
27	*Geranium wilfordii* Maxim	Protective effect on acute lung injury Wang et al. (2022a), inhibiting the proliferation of human lung cancer cell line A549 Li et al. (2016c), inhibitory effect on pneumonia pathogen Wang et al. (2013), cytotoxic activity on lung cancer cells (A549) Li et al. (2013)	Geraniin
28	*Gymnadenia conopsea* (L.) R. Br	Inhibiting silicosis progress during early exposure periods Shang et al. (2017)	-
29	*Tinospora cordifolia* Miers	Cytotoxic to A549 (Mittal et al. (2020)	-
30	*Vitis amurensis* Rupr	Inhibitory effect on the proliferation of lung cancer cells in C57BL/6 mice (Lee et al. (2006), inhibitory effect on the proliferation of A549 cells Diao. (2019), decreaseing OVA-induced lung tissue damage and mucus production in sensitized mice Li et al. (2006)	Heyneanol A, 3α-angloyloxypterokaurene L3
31	*Usnea longissima* Ach., *Usnea diffracta* Vain	Inhibitory effect on acute lung injury, protective effect on pulmonary fibrosis in mice (Su. (2015), inhibitory effect on A549 cells Guan. (2020)	Usnic acid
32	*Tremella fuciformis* berk	Inhibiting cellular apoptosis and autophagy in A549 cells Shi et al. (2018)	Tremella polysaccharides
33	*Cordyceps sinensis* (Berk.) Sacc	Inhibiting airway inflammation and improving lung function in rats with COPD (Guan and Liu. (2008), inhibiting and preventing the occurrence of pulmonary fibrosis Yang et al. (2008). Anti-non-small cell lung cancer Ji et al. (2011)	-

In this study, we found that these natural Tibetan medicines were documented to treat a variety of lung diseases, such as lung heat, pneumonia, pulmonary tuberculosis, and lung abscess. Among them, 182 (31.1%) Tibetan medicines could treat more than one type of lung disease, and 126 (21.5%) were not indicated for specific lung diseases. There are 120 (20.5%) Tibetan medicines for lung fever, 46 (7.8%) for pneumonia, and 24 (4.1%) for bronchitis. Plants of the Gentianaceae family were mostly used to treat lung fever, and some plants could also treat pneumonia and lung dryness. Orchids were mostly used to treat lung deficiency. Two plants (*Fagopyrum dibotrys* (D. Don) Hara, *Fagopyrum esculentum* Moench) were indicated to treat lung cancer. Of the 586 Tibetan medicines traditionally used in the treatment of lung diseases, only 7.2% have been experimentally demonstrated to possess various biological and pharmacological activities related to lung diseases (Table 2), such as anti-pneumonia, pulmonary fibrosis, lung cancer, and acute lung injury effects. These findings demonstrated the effectiveness of these species traditionally used to treat lung diseases. However, to date, 544 (92.8%) drugs lacked modern experimental evidence. Therefore, it is necessary for us to conduct more in-depth research on Tibetan medicines to give full play to their efficacy in treating lung diseases.

**FIGURE 5:**
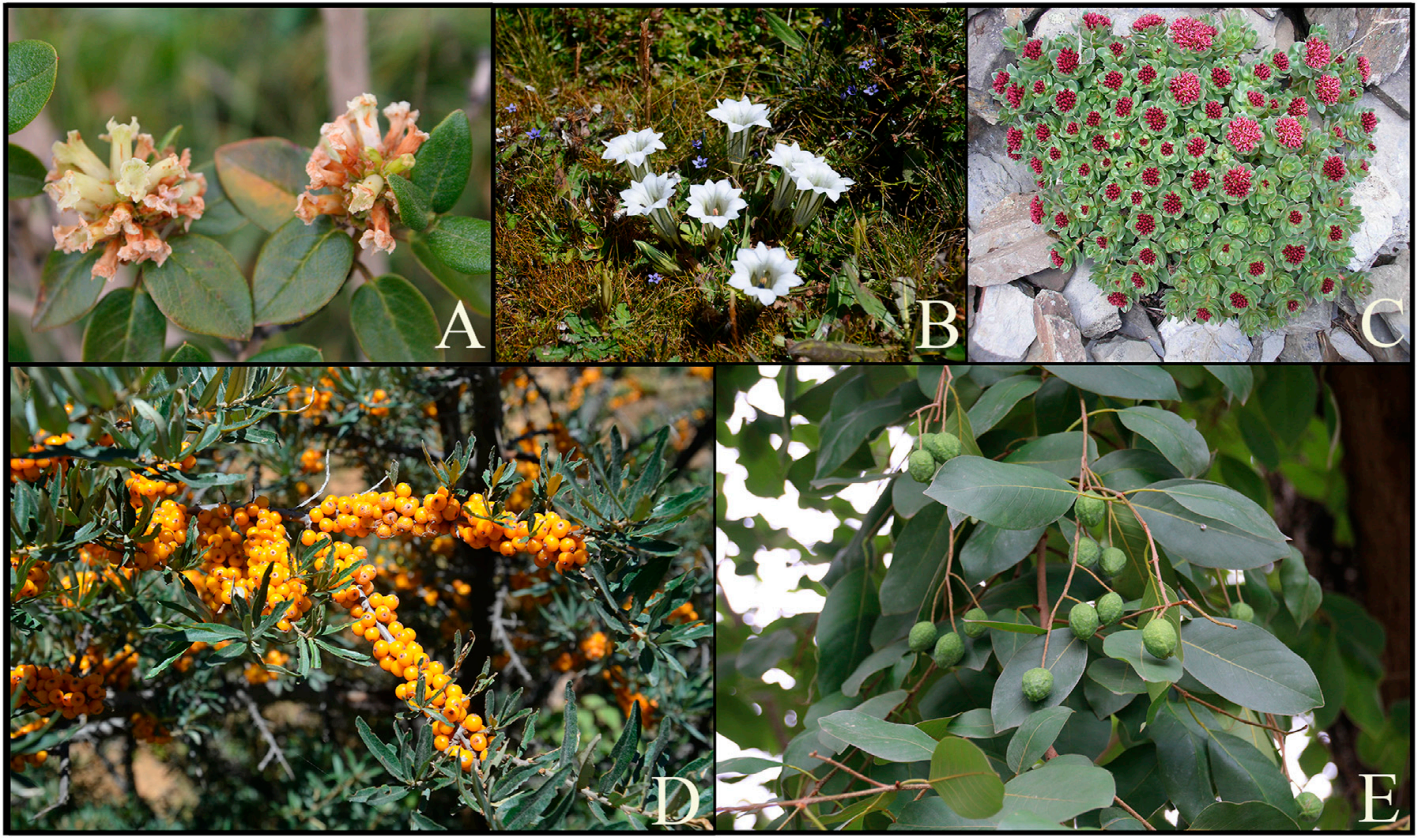
The most commonly used medicinal plants in the Tibetan medicine system to treat lung diseases. **(A)** *Rhododendron anthopogonoides* Maxim., **(B)** *Gentiana*. *szechenyii* Kanitz., **(C)** *Rhodiola crenulata* (Hook. f. et Thoms.) H. Ohba, **(D)** *Hippophae rhamnoides* subsp. *Sinensis* Rousi, **(E)** *Terminaalia chebula Retz.*

In the following section, four commonly used Tibetan medicines (Figure 5) for lung diseases and the five Tibetan medicines with the highest frequency of use in compound formulas were introduced in detail, including their names, base origins, uses, metabolites (Figure 8), and pharmacological effects. It hopes that these results can provide a basis for screening new drugs for lung diseases, provide directions and ideas for further research, and promote their global application.

### 3.3 Commonly used Tibetan medicines for lung diseases

#### 3.3.1 *Rhodiola crenulata* (Hook. f. et Thoms.) H. Ohba

Suo-luo-ma-bao (Tibetan: སྲོ་མོ་དམར་པོ།) is one of the most representative Tibetan medicine. It is called Hong Jingtian in Chinese and rhodiola in English. The medicinal parts are the dried roots and rhizomes of *Rhodiola crenulata*. In “Jing Zhu Materia Medica”, it was written that Suo-luo purifies lung heat and was divided into three kinds: white, purple, and red, of which the red one was Suo-luo-ma-bao. The “Wendaohe” said: “Solomabao grows in high mountains, rocky mountains, and other places; wherever it is born, the stems are red, harder, most, thick leaves, clusters, turning red in autumn; flowers, fruit pods, seeds are red; roots like human lung color; thick skin, big smell; taste sweet, bitter, astringent, cool; effects nourish the lungs, clear heat, nourish the vital energy.” Rhodiola is rich in chemical components, mainly containing tyrosol and its glycosides, flavonoid components, cyanogenic glycosides, coumarins, and so on. Among them, the active ingredient specified in the pharmacopeia is salidroside.

As a commonly used Tibetan medicine for the treatment of lung diseases, there are many pharmacological and clinical studies on rhodiola and its extract salidroside. In recent years, it has been found that rhodiola has various pharmacological activities for the treatment of chronic obstructive pulmonary disease, anti-lung cancer, pulmonary fibrosis, lung injury, *etc. In vitro* studies found that salidroside could protect the *in vitro* cellular model of chronic obstructive pulmonary disease through antioxidant mechanism (Hou et al., 2020). *In vivo* studies have found that salidroside may inhibit NF-κB signaling and reduce pIκBα and p-NF-κBp65 protein expression in the lungs of COPD mice, thus effectively protecting against lung injury in COPD mice (Carozzi et al., 2017). The study by Zhang et al. confirmed that salidroside effectively reduced the protein expression of myostatin, increased the protein expression of myogenin and p-akt protein content, increased cytochrome C oxidase and ATP synthase activities, and enhanced mitochondrial energy metabolism in rats with COPD, thereby improving skeletal muscle dysfunction in rats with COPD, further explaining the rationale for the effect of rhodiogenic on alleviating symptoms in COPD patients (Zhang et al., 2019). Recent clinical studies have confirmed that rhodiola combined with Western medicine for COPD can alleviate symptoms and improve prognosis to some extent. Clinical observation by Li L et al. confirmed that rhodiola injection combined with simvastatin could better improve patients’ pulmonary function, arterial blood gas performance, effectively alleviate symptoms, and reduce inflammatory response (Li et al., 2016a). According to a clinical study by Zhao., ligustrazine injection combined with radix injection of large strains improved clinical symptoms and pulmonary function and reduced pulmonary artery systolic pressure better than using atorvastatin calcium tablets (Zhao, 2015). According to Zhang et al., patients treated with rhodiola combined with Western medicine showed a shorter time taken to recover from symptoms such as wheezing, edema, and impaired consciousness, with significant improvement in pulmonary function and APACHE II scores and no significant adverse effects (Zhang, H.Y. et al., 2014). In a study by Qu et al., it was demonstrated that rhodiola combined with conventional Western medical treatment regimens could greatly improve the oxygenation status and symptoms of patients with acute exacerbations of COPD (Qu et al., 2014).

**FIGURE 6:**
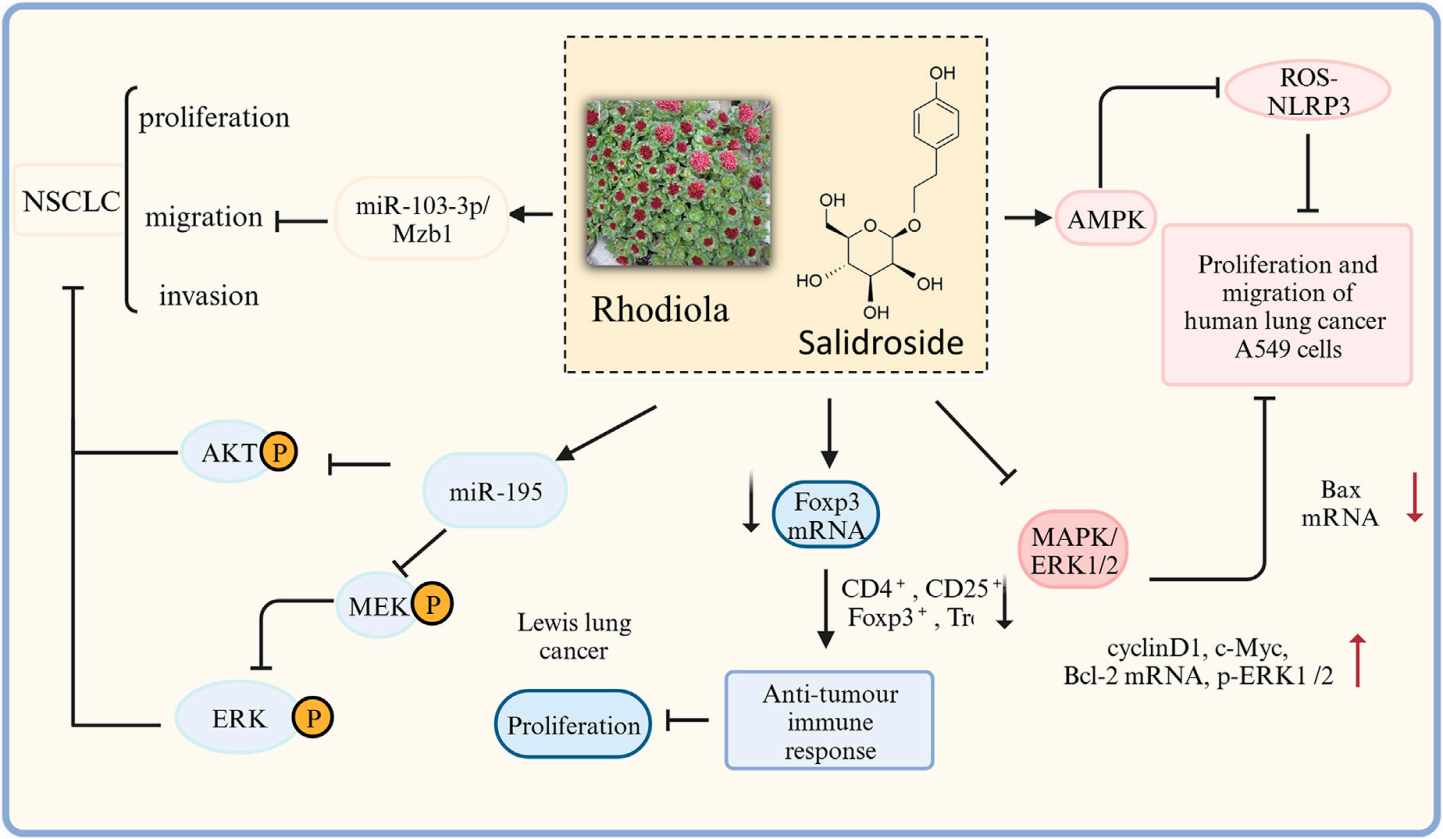
The therapeutic effects and potential mechanisms of salidroside on Lung cancer. Salidroside represses proliferation, migration and invasion of human lung cancer cells: through AKT, MEK/ERK, microRNA-103–3p/Mzb1 and MAPK/ERK1/2 signal pathway; salidroside inhibits the proliferation and migration of HUMAN lung cancer A549 cells through AMPK dependent inhibition of ROS-NLRP3 inflammasome signaling.

Numerous studies have confirmed that rhodiola extract and salidroside exert anti-lung cancer effects through multiple mechanisms of action (Figure 6). *In vitro* studies have shown that rhodiola extract may inhibit lung cancer cell proliferation, migration, epithelial-mesenchymal transition (EMT), and promote apoptosis by activating the TGF-β/Smads signaling pathway, upregulating E-cadherin and Caspase-3 expression, and inhibiting Vimentin and Bcl-2 expression related (Huang et al., 2023). In addition, Zhu et al. found that salidroside inhibits the proliferation and metastasis of lung cancer cells also by regulating target genes of aberrant microRNAs (miR103–3p/Mzb1) (Zhu et al., 2020). Wang, (2022) found that salidroside played an inhibitory role in the proliferation and migration of human lung cancer A549 cells through AMPK-dependent inhibition of ROS-NLRP3 inflammatory vesicle signaling. Salidroside block AKT and MEK/ERK signaling pathways by upregulating miR-549 expression in A195 cells, thereby inhibiting the proliferation, migration, and invasion of Non-Small-Cell Lung Cancer (NSCLC) cells (Ren et al., 2019). Zhang et al. showed in experimental Lewis lung cancer mice that rhodiola extract might enhance the antitumor immune response by down-regulating the proportion of CD4^+^ CD25^+^ Tregs and the mRNA expressions of Foxp3 and TGF-β in the tumor tissues (Zhang et al., 2013a; Zhang et al., 2013b). Li et al. found that salidroside inhibited the growth of human lung cancer A549 cell nude mice transplanted tumors, disrupted tumor cell structure, and promoted apoptosis of tumor cells, presumably by a mechanism that may be related to the inhibition of MAPK/ERK1/2 signaling pathway, upregulation of Bax mRNA and protein expression levels, downregulation of cyclinD1, c-Myc, Bcl-2 mRNA and protein expression levels, and reduction of p-ERK1/2 levels (Li et al., 2020a).

**FIGURE 7:**
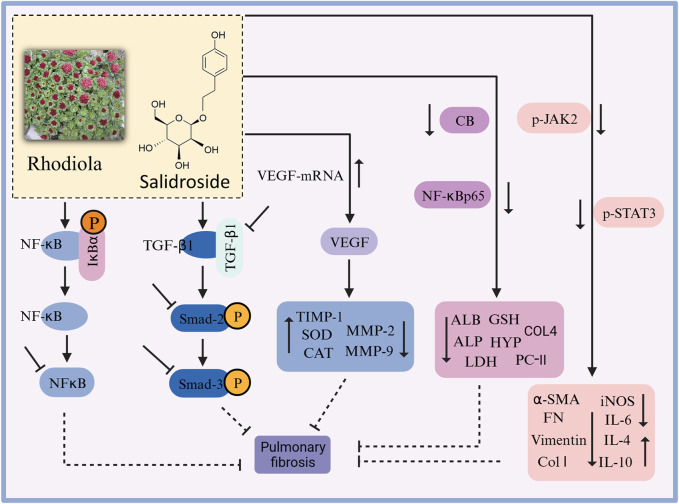
The therapeutic effects and potential mechanisms of salidroside on pulmonary fibrosis. Salidroside protects against pulmonary fibrosis: activation of Nrf2-antioxidant signaling, inhibition of NF-κB and TGF-β1/Smad-2/-3 pathways, upregulation of CEGF protein expression, and downregulation of CB, NF-κBp65, p-JAK2, and p-STAT3 protein expressions.

In recent years, there have been many studies on the mechanism of action of rhodiola against pulmonary fibrosis (Figure 7). *In vitro* studies revealed that salidroside induced the transformation of A549 cells to normal cells, reduced cell mortality, and inhibited the expression of the fibronectin EDA fragment (Zhang, Z.Y. et al., 2014a). *In vivo* studies are as follows. Zhang et al. demonstrated that rhodiola prevented bleomycin-induced pulmonary fibrosis in rats through anti-inflammatory, antioxidant, and anti-fibrotic properties (Zhang et al., 2016a). It has been shown (Li, 2015) that salidroside inhibited the bleomycin-induced increase in serum hydroxyproline and TGF-β1, prevented the bleomycin-induced decrease in pulmonary superoxide dismutase (SOD), peroxidase (POD) and catalase (CAT), and reduced pulmonary IV collagen, malondialdehyde (MDA) and TGF-β1 content, thus improving pulmonary fibrosis in rats. It also improved the enzymatic activities of SOD and CAT, and regulated the balance of matrix metalloproteinases (MMPs) and MMP inhibitors (TIMPs), thus improving bleomycin-induced pulmonary fibrosis in rats (Li et al., 2016b). In addition, it has been shown that salidroside reduced serum hydroxyproline, collagen type IV and malondialdehyde levels in lung tissues of mice with bleomycin-induced pulmonary fibrosis, while TGF-β1 levels in serum and lung tissues were significantly reduced, and pulmonary solids and collagen deposition were alleviated (Liu et al., 2017a). Salidroside ameliorate CLP-induced pulmonary fibrosis in mice with acute lung injury from sepsis by downregulating p-JAK2 and p-STAT3 protein expression (Guo et al., 2022); also by regulating collagen-related protein (Huang et al., 2018). Zhang (2015) found that salidroside significantly reduced the expression of MMP-2 and TIMP-1 mRNA and protein (*p* < 0.05) and attenuated the pathological changes of paraquat-induced fibrosis in rats. It has been shown that salidroside can inhibit transforming growth factor β1 (TGF-β1)/Smad-2/-3 and cytosolic nuclear factor κB (NF-κB) pathways, activate Nrf2 antioxidant signaling pathway, and improve pulmonary fibrosis injury in rats (Tang et al., 2016), as well as improve symptoms of acute lung injury and stop the development of pulmonary fibrosis inhibit by inhibiting the expression of TGF-β1-encoded genes and inflammatory cell infiltration (Zhang et al., 2014b).

**FIGURE 8:**
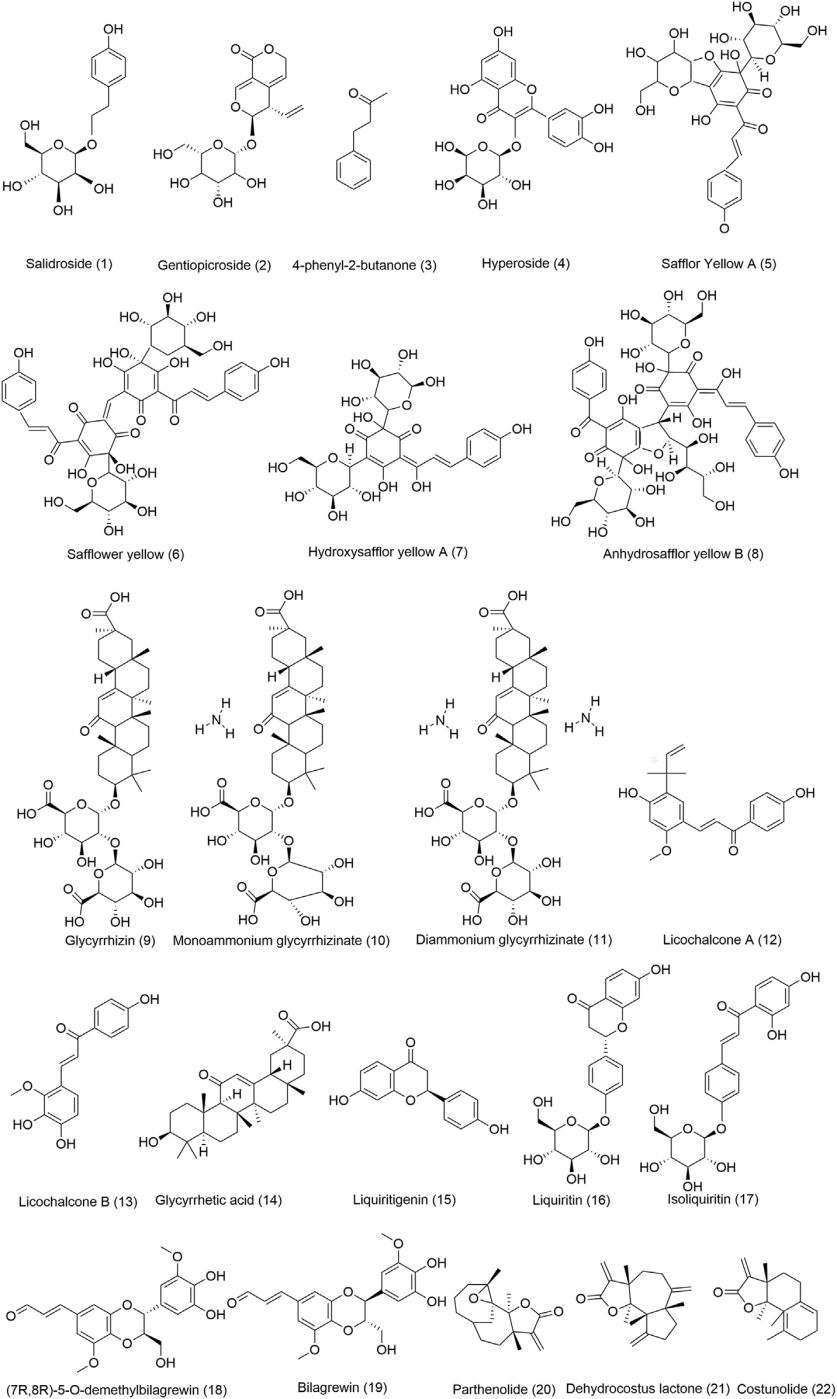
Chemical structures of the main TTM bioactive components with anti-lung diseases. (1) Chemical composition comes from rhodiola, (2) Chemical composition comes from *Gentiana veitchiorum,* (3-4) Chemical compositions come from *Rhododendron anthopogonoides,* (5-8) Chemical compositions come from *Carthamus tinctorius* L., (9-17) Chemical compositions come from *Glycyrrhiza uralensis* Fisch, (18-19) Chemical compositions come from *Santalum album* L., (20-22) Chemical compositions come from *Aucklandia lappa* Decne.

*In vivo* studies have shown that rhodiola and its extract salidroside have shown ameliorative effects on acute lung injury, hyperoxia-induced lung injury (Zhu et al., 2021a), intermittent hypoxia (IH)-induced lung injury (Wu et al., 2019), and acute progressive plateau lung injury (Wang et al., 2022a). Salidroside may improve lung function in mice with acute lung injury from CLP-induced sepsis by inhibiting caveolin-1 and TLR/NF-kappa B pathways *in vivo* inhibiting caveolin-1 and TLR/NF-kappa B pathways *in vivo* (Liu et al., 2017b). It has been shown that synthetic salidroside protects against acute lung damage in rats by inhibiting NF-κB protein phosphorylation in lung tissue and by reducing polymorphonuclear (PMN) aggregation in the lung (Huang et al., 2017). Salidroside is protective against hyperoxia-induced lung injury in mice, and their mechanism of action may be related to changes in the expression of the Notch signaling pathway (Zhu et al., 2021b). Rhodiola protects against IH-induced lung injury, probably through anti-oxidative stress, mitigation of hypoxic damage, and inhibition of inflammatory response pathways (Wu et al., 2019).

*In vivo* studies have shown that salidroside improved the inflammatory response of lung tissue in rats with severe pneumonia, reduced the release of inflammatory mediators and inflammatory cell infiltration in a dose-dependent manner, and its effects were associated with reduced levels of NF-κB and p38 signaling pathway activation (Wang et al., 2021a). A study by Zhao et al. explained the anti-inflammatory and immune-enhancing effects of rhodiola. Salidroside showed dose-dependent effects on reducing right heart hypertrophy index (RVHI), serum interleukin-6 (IL-6), tumor necrosis factor-α (TNF-α), and p-NF-κB/NF-κB and p-p38MAPK/p38MAPK in lung tissues of mice with hypoxic pulmonary hypertension model, and improved cardiopulmonary function in mice (Zhao et al., 2022). Salidroside inhibited the development of emphysema in mice by suppressing cell scorching in lung tissues (Tang, 2022). A clinical study by Lei et al. reported that adjuvant treatment of patients with pulmonary embolism combined with heart failure using rhodiola injection improved the overall effective cure rate, reduced serum amino-terminal brain natriuretic peptide precursor (NT-proBNP) levels, and improved patients’ blood gas indices and echocardiographic parameters (Lei et al., 2019). The clinical efficacy can be further improved by using rhodiola injection in addition to the conventional treatment plan for pulmonary embolism combined with heart failure. The above results suggested that rhodiola may be a candidate for the treatment of a variety of lung diseases.

Rhodiola contains multiple components, but currently only salidroside has been studied for its pharmacological effects, and preclinical studies on other components are needed. Preclinical and clinical studies have shown that rhodiola extract and salidroside can treat various lung diseases, including COPD, lung cancer, pulmonary fibrosis, lung injury, pneumonia, emphysema, *etc.* Rhodiola may have a good potential to develop into a candidate drug for the treatment of various lung diseases.

#### 3.3.2 *Gentiana veitchiorum* Hemsl., *G.przewalskii* Maxim., *G*. *szechenyii* Kanitz., *G*. *algida* Pall., *G. stipitata* Edgew

Bang-jian (Tibetan: སྦལ་ལ་ནག་པོ།)is a common Tibetan medicine used to treat lung diseases. , which comes from a variety of vegetal (medicinal) species in the Gentian family. It is called gentian in English. *Gentiana veitchiorum*, *G. przewalskii*, *G*. *szechenyii* Kanitz, *G*. *algida*, *G. stipitata*. are the most commonly used vegetal (medicinal) species of Bang-jian. According to “Jing Zhu Materia Medica”, there are three species of Bang-jian according to the color: white flower, blue flower and black flower, which are called " Bang-Jian Gabao”, " Bang-Jian Wanbao” and " Bang-Jian Nabao” respectively; white flower gentian grows in the cold zone of high mountains, blooming in late autumn, without stems, with leaves similar to those of *Gentiana macrophylla*, with four to five flowers from the ground, with red luster, and the flowers are united at the base, or born in grassland on the mountain slope, with small leaves and flourishing flowers; blue flower gentian is born in very wet swampy grassland in early autumn, with a similar form as white flower gentian, with small leaves and obvious light blue flowers. Ranjun duoji said: “Bang-Jian Ga Bao clear lung heat, blue flower Bang-Jian with the same effect and cool nature”. Among these five vegetal (medicinal) species, the first two are blue-flowered Bang-jian and the last three are white-flowered Bang-Jian. The dried above-ground parts with flowers are used in the medicine. It is bitter and cool in nature and is used to treat fever, red eyes and sore throat, lung fever and cough, gastritis, meningitis, bronchitis, *etc.*

Fu (2018) divided the mainstream species into two types: among them, type I was represented by *G. szechenyii,* which had little components such as swertiamarin, gentiopicroside, and sweroside while contained gentiournosides D, depressine, gentizechenlioside A and szechenyin A. Type II are mainly from *G. algida*, *G. purdomii* and others containing no iridoid moiety of the benzoyl moiety, but components such as swertiamarin, gentiopicrin, and sweroside were present, and the content of these components in type II is generally higher, and its surrounding chemical composition was more. Flavonoids such as isoorientin, isoscoparin-2″-*β-D*-glucopyranoside and isoscoparin were found in both chemical types, and their contents were generally higher in type II.

At present, only *G. veitchiorum* and its extract gentiopicroside have been studied in modern pharmacology. In a vitro experiment, TGF-β was found to stimulate the inhibited epithelial-mesenchymal transition of lung cancer A-549 cells in a dose-dependent manner, and gentiopicroside significantly downregulated the expression of TGF-β and connective tissue growth factor in mouse lung tissue. Therefore, gentiopicroside inhibits the transformation of lung cancer A-549 cells. On the other hand, gentiopicroside has anti-inflammatory and anti-fibrotic effects, in which alveolar epithelial cells and TGF-β may be the main target cells and molecules of gentiopicroside for the treatment of bleomycin-induced pulmonary fibrosis, respectively, and therefore, it may be an ideal candidate for the treatment of pulmonary fibrosis (Chen et al., 2018). By comparing the therapeutic effects of different polar components on mouse chronic bronchitis model and pulmonary fibrosis cell model, effective components of gentian extract for treating chronic bronchitis and pulmonary fibrosis were screened out (Geng, 2010). The results showed that the ethyl acetate and n-butanol extracts of *G. veitchiorum* extracts were effective in the treatment of pulmonary fibrosis. In addition, the blue yuzan granules made from *Gentiana veitchiorumas* the main fraction and Panax ginseng total saponin as a supplement were effective in treating pulmonary fibrosis caused by acute and chronic respiratory system inflammation (Shi et al., 2016). Hou et al. reported that blue yuzan granules may affect the expression of TNF-α through the ERK pathway, thus achieving an inhibitory effect on inflammation, providing a further experimental basis for the mechanism of action of blue yuzan granules in the treatment of chronic bronchitis (Hou et al., 2011).

Gentian originates from various vegetal (medicinal) species and contains numerous metabolites, but only gentiopicroside has been proven to have anti-lung cancer and therapeutic effects on pulmonary fibrosis. The crude extracts of *G. veitchiorumas* and blue yuzan granules can treat chronic bronchitis. Further in-depth and comprehensive research is required on the material basis and mechanism of its pharmacological effects.

#### 3.3.3 *Hippophae rhamnoides* L., *Hippophae thibetana* Schlechtend, *Hippophae rhamnoides* subsp. Sinensis Rousi, *Hippophae neurocarpa* S. W. Liu et T. N. He

Da-bu (Tibetan: སྟར་བུ།)is called sea buckthorn in English, which is widely used in Tibetan medicine clinics. The most commonly used vegetal (medicinal) species of Tibetan medicine Da-bu include *Hippophae rhamnoides*, *Hippophae Thibetana*, *H. rhamnoides* subsp. Sinensis, and *Hippophae neurocarpa*. Drug Standards of Tibetan Medicine (Commission, 1995) recorded that sea buckthorn paste (Tibetan: སྟར་བུ།)is a decoction of the mature fruit of *H. rhamnoides* L. in the family Elaeagnaceae. According to“Jing Zhu Materia Medica”, Da-bu can remove lung tumors, transform blood, and treat “Baekan (བད་ཀན།) disease”. Da-bu contains many chemical components such as glycosides, flavonoids, phenols, tannins, amino acids, polypeptides and proteins, and organic acids. Among them, total flavonoids and isorhamnetin are the active ingredients stipulated in the pharmacopeia.

Total flavonoids of sea buckthorn are the main pharmacological substance basis for its therapeutic effect. At present, scholars have studied the total flavonoids of sea buckthorn through *in vitro* experiments and network pharmacology, and found that total flavonoids of sea buckthorn have the effect of inhibiting the proliferation of lung cancer cells and treating chronic bronchitis. Jia C et al. investigated the effects of the total flavonoids of *Hippophae thibetana*, *H. rhamnoides*, and *H. neurocarpa* on the proliferation and migration of non-small cell lung cancer A549 cells, and explored their molecular mechanisms of action (Jia et al., 2020). Ren (2019) investigated the pharmacodynamics and mechanism of action of sea buckthorn total flavonoids in the treatment of chronic bronchitis and found that total flavonoids of sea buckthorn also significantly improved the LPS-induced reduction of body mass in mice and reduced the elevation of macrophage, neutrophil and total cell counts in alveolar lavage fluid, which had therapeutic effects on chronic bronchitis. The results of network pharmacology and molecular docking showed that quercetin, isorhamnetin, and kaempferol exerted their therapeutic effects on chronic bronchitis by blocking the Fc epsilon RI signaling pathway, mitogen-activated protein kinase signaling pathway, and vascular endothelial growth factor signaling pathway. Although sea buckthorn is a commonly used Tibetan medicine for treating lung diseases in the Tibetan medical system, current research on its treatment of lung diseases is not sufficient, especially in the absence of pharmacological studies on single pharmacodynamic components. More scholars are needed to devote themselves to the research of the Tibetan medicine sea buckthorn to promote the development of related new drugs.

#### 3.3.4 *Rhododendron anthopogonoides* Maxim., *R. cephalanthum* Franch., *R. primulaeflarum* Bur. Er Franch

Da-li (Tibetan: ད་ལིས།)comes from a variety of vegetal (medicinal) species. Drug Standards of Tibetan Medicine (China, 1995) recorded that Da-li is the dried flowers and leaves of *Rhododendron anthopogonoides*, *R. cephalanthum*, *R. primulaeflarum*. It is said in the “Thirty Chapters of the Ming Explanation” that Da-li is warm and mild in nature, and is used to treat Baekan disease, painful lung disease, and eruptions. The “Illustrated Book” said: “Da-li born in the shade of high mountains. The trunk is white and the fruit tastes sweet, bitter and astringent. It is used to treat Loong disease, Tripa disease, Baekan disease, and lung disease”. In 1971, it was introduced to various parts of China for the treatment of chronic bronchitis (Fan et al., 2016). Da-li contains volatile oils such as 4-phenyl-2-butanone, flavonoids such as hyperoside, and triterpenoids such as ursolic acid (Ji et al., 2002).

Notably, *R. anthopogonoides* and the phytochemicals or extracts obtained from *R. anthopogonoides* have been shown to have some pharmacological activities associated with lung diseases. As a commonly used Tibetan medicine for lung diseases, *R. anthopogonoides* has good efficacy in coughing and phlegm asthma, which are common symptoms of lung diseases. The research group of Lanzhou Medical College for the prevention and treatment of chronic bronchitis found that flavonoids in the ethanolic extract of *R. anthopogonoides* had an inhibitory effect on *Streptococcus* pneumonia (College, 1973a). *In vivo* experiments, *R. anthopogonoides* could reduce mucosal swelling, relax the trachea and bronchi, and accelerate the speed of mucus operation of tracheal cilia, thus having good expectorant and asthma-calming effects (College, 1973a). Li et al. demonstrated that 4-phenyl-2-butanone in the volatile oil of *R. anthopogonoides* has obvious cough suppressant, expectorant and asthmatic effects, which is a central cough suppressant, and its cough suppressant strength is slightly weaker than 1/6 dose of codeine (Li et al., 1980). The phenol red test in mice demonstrated the significant expectorant effect of flavonoids of *R. anthopogonoides* Maxim by intraperitoneal injection, gavage, spray, and intratracheal drip (College, 1973b). Zhang, (1979) found out through *in vivo* experiments that hyperoside, 4-phenyl-2-butanone, a chemical constituent in *R. anthopogonoides*, had cough suppressant effects. In addition, the clinical treatment of chronic tracheitis was clinically treated with anthorhododendrin, and the results showed that the expectorant effect was more prominent (College, 1973a). However, there are no reports of *R. cephalanthum*, *R. primulaeflarum* for the treatment of lung disease. Therefore, it is necessary to conduct phytochemical, pharmacological, and clinical studies on *R. cephalanthum*, *R. primulaeflarum* to verify their efficacy in the treatment of lung diseases and, at the same time, to lay the foundation for the development of new drugs for the treatment of lung diseases. Preclinical studies have shown that some components in the volatile oil of *R. anthopogonoides* have expectorant and asthma-calming effects. Further pharmacological studies can be conducted on other components in volatile oil to fully develop and utilize them.

### 3.4 The five most frequently used Tibetan medicines in Tibetan medicine prescriptions

#### 3.4.1 *Carthamus tinctorius* L

The Tibetan name of *Carthamus tinctorius* L. is སྦལ་ལ་ནག་པོ།, the Chinese name is Honghua, and the English name is safflower. Safflower is spicy in taste and warm in nature and is used as a medicine for pneumonia and tuberculosis with dried flowers (Jia and Zhang, 2016). Safflower is native to the ‘New Crescent’ on the east coast of the Mediterranean and has been cultivated for over 4,500 years (Ren et al., 2017). It is recorded in the “Compendium of Materia Medica” that Zhang Qian introduced safflower to China during his mission to the West (via the Silk Road) during the Han Dynasty. As a result, safflower has been grown and used in China for more than 2000 years. Currently, safflower is widely grown in Asia, Europe, Australia, and America (Ren et al., 2022). Safflower contains a variety of flavonoids, such as safflor yellow A (SYA), safflower yellows B, hydroxysafflor yellow A (HSYA), cathamone, safflomin A, kaempferide, luteolin, etc (Zhong and Song, 2020). Among them, HSYA and kaempferide are the main bioactive prescriptions, which are often used as markers for the quality control of safflower in the pharmaceutical industry and drug standards.

In addition, there have been many reports on the pharmacological activity of safflower for the treatment of lung diseases. *In vitro* studies, safflower injection significantly inhibited proliferation migration and induced apoptosis in A549 cells, and the related mechanism may be related to the GSK-3*β*-NF-KB-Snail pathway (Qiao et al., 2020). *In vivo* studies of safflower injection and safflower monomer components are as follows. Several investigators found that safflower injection may reduce bleomycin-induced pulmonary fibrosis in mice by inhibiting the TGF-β1/Smad3 signaling pathway and downregulating α-SMA expression (Luan et al., 2019; Luan et al., 2021). Bao et al. found that safflower yellow significantly improved COPD in model rats by inhibiting the expression of inflammatory factors and regulating the expression of TLR4/NF-KB pathway-related proteins (Bao et al., 2021). Wang found that SYA, HSYA, and anhydrosafflor yellow B (AHSYB) ameliorated acute lung injury by inhibiting the formation of neutrophils (NETs) and the Raf/MEK/ERK pathway (Wang, 2020). The above studies suggested that safflower has potential effects in the treatment of pulmonary fibrosis, lung cancer, COPD, and acute lung injury.

#### 3.4.2 *Glycyrrhiza uralensis* Fisch

The dried roots and rhizomes of *G. uralensis* known as Xiang-an (Tibetan:སྦལ་ལ་ནག་པོ།), Gancao (Chinese name), or licorice (English name), is commonly used herbal medicine. Xiang-an is sweet in taste and mild in nature and treats lung diseases and bronchitis. It is born in sandy soil at 800–2,800 m altitude and is distributed in Golmud in Qinghai, Gansu, Xinjiang, and Inner Mongolia. Flavonoids are one of the main components of licorice extract, and are also important components reflecting the main medicinal value of licorice. The triterpenoid saponins in licorice are its specific markers, among which glycyrrhetinic acid is a high content. Licorice polysaccharide is the third biologically important substance in addition to licorice flavonoids and saponins (Wang et al., 2022b). At present, many prescriptions have been extracted and isolated from licorice, such as isoglycyrrhizinol, licorice coumarin, liquiritin, glycyrrhizin, glycyrrhetic acid, and so on. Among them, liquiritin and glycyrrhizin are often used as markers for quality control of licorice in the pharmaceutical industry and drug standards.

**FIGURE 9:**
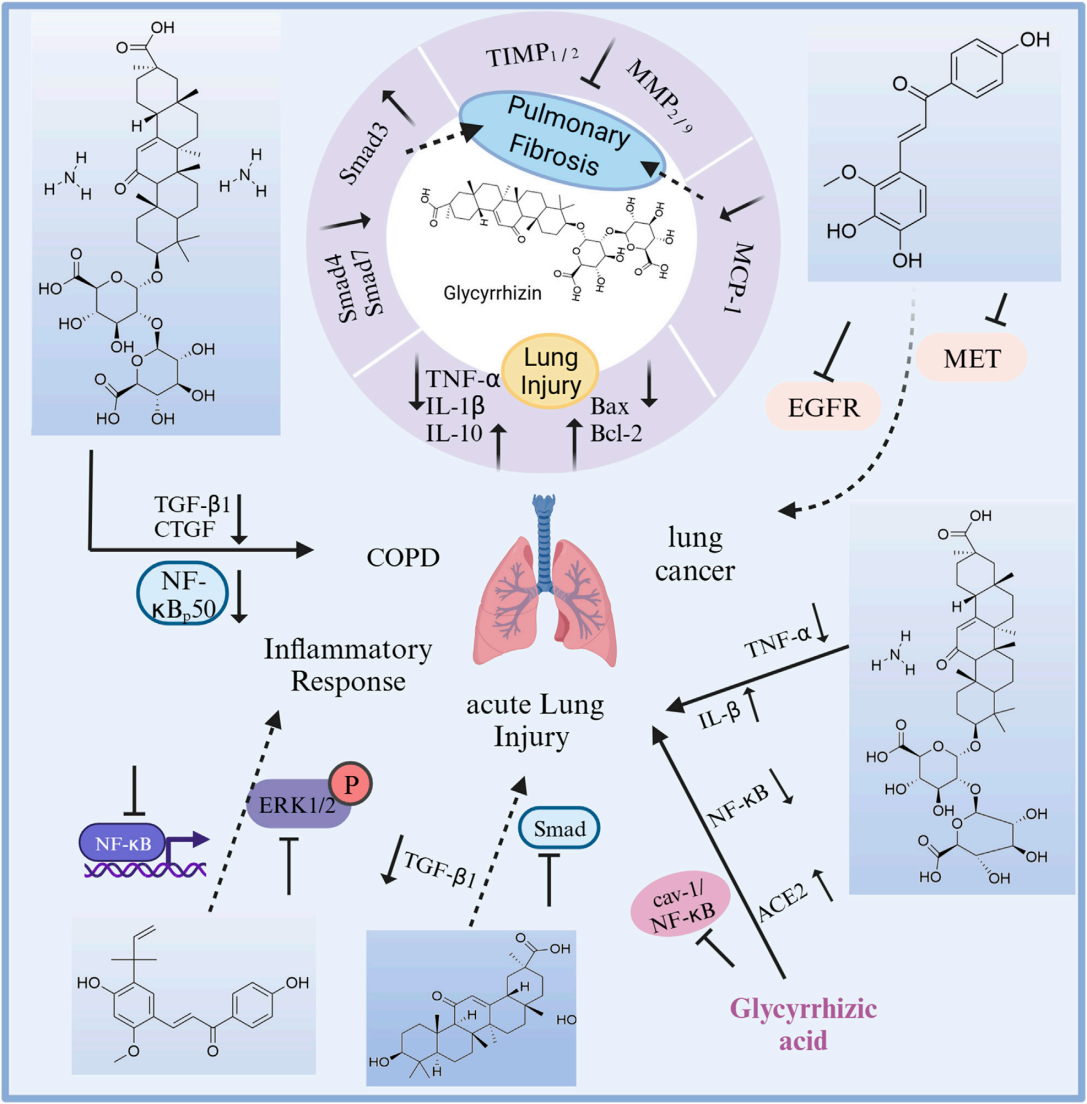
The therapeutic effects and potential mechanisms of chemicals from licorice on lung diseases. Glycyrrhizin protects against pulmonary fibrosis: reversing the abnormal expression of Smad family, and downregulation of MCP-1 expressions. Glycyrrhizin protects against lung injury: regulation on inflammation and cell apoptosis.

Licorice has a wide range of pharmacological activities, including antibacterial, anticancer, antiviral, anti-inflammatory, memory-enhancing, neuroprotective, and pulmonary protective effects, as well as hypoglycemic and cholesterol-lowering effects. To date, many scholars have conducted antiacute lung injury, chronic lung injury, and pulmonary fibrosis studies on licorice (Figure 9). Total flavonoids are one of the effective sites of licorice against acute lung injury. *In vitro* studies have shown that total flavonoids of licorice inhibited the secretion of eosinophil-activated chemokine-1 by human lung fibroblasts, which was expected to block the recruitment of eosinophils to the site of antigenic inflammation (Jayaprakasam et al., 2009). *In vivo* studies of total flavonoids of licorice and monomeric components are as follows. Total flavonoids of were effective against lipopolysaccharide-induced pneumonia (Xie et al., 2009; Xie and Guan, 2009). It was found that both Glycyrrhiza endophytes and licorice produce cough expectorant, improve lung function and reduce lung injury by upregulating water channel protein expression and downregulating inflammatory factor expression in lung tissue (Wang et al., 2021b). Licochalcone A could treat acute lung injury in mice by blocking the ERK/NF-κB signaling pathway, inhibiting inflammatory factor overexpression and correcting oxidative/antioxidant imbalance (Furusawa et al., 2009; Chu et al., 2012; Ren et al., 2016). In addition, the triterpenoid components glycyrrhizin, glycyrrhetic acid, and diammonium glycyrrhizate also had anti-pulmonary fibrosis and lung injury protection effects. Glycyrrhizin and monoammonium glycyrrhizinate produced protective effects against acute lung injury by regulating the release of pro- and anti-inflammatory factors and by inhibiting apoptosis (Tang et al., 2007; Wang et al., 2012); and by inhibiting the cav-1/NF-κB signaling pathway through upregulation of ACE2 expression (Li, 2018b; Chen, 2019). Glycyrrhizin blocked pulmonary fibrosis by downregulating the expression of monocyte chemokine-1 and inhibiting the secretion of growth factors such as TGFβ1, thereby suppressing collagen fibril synthesis and fibroblast proliferation (He et al., 2006; He et al., 2008; 2009), and by inhibiting the IL-17/TGFβ1/Smad pathway in pulmonary fibrosis (Ye et al., 2007). Glycyrrhetic acid produced anti-acute radiation lung injury by blocking the TGFβ1/Smad signaling pathway (Yang, 2014). Diammonium glycyrrhizate played anti-pulmonary fibrosis effects by reducing the secretion of TGFβ1 and connective tissue growth factor in lung tissue (Li, 2018b), and could reduce chronic lung injury through anti-inflammatory effects (Bu et al., 2010).

In addition, the active ingredients in licorice also can induce apoptosis in lung cancer cells and have anti-viral activity. Li et al. predictive analysis of quality markers (Q-Marker) of licorice showed that flavonoids such as glycyrrhizin, isoglycyrrhizin, glycyrrhizin, triterpenoids such as glycyrrhizin and glycyrrhizic acid, and glycyrrhizin coumarin and glycyrrhizin polysaccharides were resistant to SARS (Li et al., 2021). An *in vivo* study had shown that Licochalcone B could induce apoptosis in non-small cell lung cancer cells (Oh et al., 2019). *G*. *uralensis* extract and glycyrrhetinic acid have been clinically effective in the treatment of severe acute respiratory syndrome (SARS) and COVID-19 as well as asthma, lung injury during surgical extracorporeal circulation, *mycoplasma* pneumonia, bronchopulmonary dysplasia and COPD, but these studies are only preliminary clinical observations and a large number of clinical observations with rigorous experimental designs are needed in the future (Zhang and Shen, 2020).

In summary, the flavonoids and triterpenoids in licorice have a wide range of pharmacological activities against lung diseases, such as anti-lung injury, anti-pulmonary fibrosis, anti-lung cancer, anti-pneumonia, and anti-COPD. Licorice may have great potential for development and become a candidate drug for treating lung diseases.

#### 3.4.3 *Santalum album* L

The medicinal part of Zan-dan-ga-bao (Tibetan: སྦལ་ལ་ནག་པོ།) is the dried heartwood of *Santalum album*, which is called Tan-Xiang in Chinese, and sandalwood in English. “The Drop of Manna” recorded that it is cool in nature, slow-acting, light and dry, and used for the treatment of lung fever, pneumonia, and lung abscess. *S. album* is not cultivated on the Qinghai-Tibet Plateau and is mostly imported from India, Australia, and Indonesia. It is also grown in Hainan and Taiwan in China. Tibetan medicine classifies sandalwood into three types: white, slightly yellow, and red sandalwood. Santalum album L. is the white one, and the name “white sandalwood” is also used in prescriptions. Tibetan doctors also use *Syringa reticulata (Blume)* var. *mandshurica* (Maxim.) Hara is a substitute for lignum vitae (Zhong and Song, 2020).

Sandalwood mainly contains sesquiterpenoids, monosteroids, and lignin prescriptions. Among them, α-sandalol and β-sandalol are its characteristic chemical components (Zhang et al., 2020). At present, scholars at home and abroad have found that the terpenoids in the volatile oil of sandalwood have a wide range of anti-tumor and anti-cancer effects, and santalol plays an important anti-cancer role in cancer cells. Meanwhile, some *in vitro* studies have reported that the lignans in sandalwood also have some anti-lung cancer cell effects. For example, Matsuo and Mimaki isolated two lignin-like components ((*7R,8R*)-5-*O*-demethylbilagrewin, bilagrewin) from sandalwood and investigated their antitumor activity, showing that both prescriptions were highly cytotoxic against HL-60 human leukemia early juvenile granulocytes and A549 human lung adenocarcinoma cells (MATSUO and MIMAKI, 2020). The above studies suggest that sandalwood may have good potential as a drug candidate for the treatment of lung cancer.

#### 3.4.4 *Aucklandia costus* Falc

Ru-da (Tibetan: སྦལ་ལ་ནག་པོ།), known as Muxiang (Chinese name), costus (English name), is the dried root of *Aucklandia costus* Falc. This medicine is spicy, warm in nature, and has a sharp effect. “Jing Zhu Materia Medica” recorded: “Ru-da treats gas and blood, stomach distention, lung diseases, throat problems, and removes dead flesh. This medicine is divided into black and white. White Ru-da is produced in the land of Kangmu. Black Ru-da is produced in India and the Gondas, and is available in both mountain and cultivated varieties, with an aromatic scent.” “Tujian snail’s eye” recorded that “Black and white Ru-da like shed antlers”. The plant is planted in mountains and hills at altitudes of 2,500–4,000 m. It is native to India and was later introduced to Yunnan, and it is cultivated in most provinces. So far, the chemical components isolated from *Aucklandia lappa* mainly include sesquiterpene lactones and organic acids. Among them, costunlide (CNL), and dehydrocostus lactone (DHC) are usually used as the marker for controlling the quality of costus in the pharmaceutical industry and drug standards.

Many *in vitro* studies have shown that a variety of active ingredients in woody incense have antitumor activity. Numerous studies have shown that vascular endothelial growth factor (VEGF) is overexpressed in many cancer tissues, including liver, lung, colon, ovarian, and breast cancers. Hao LJ et al. found that the volatile oil, DHC, and costunolide inhibited the secretion of VEGF from human lung adenocarcinoma A549 cells (Hao et al., 2010). Parthenolide can significantly induce apoptosis and inhibit the invasion and migration of non-small cell lung cancer H1975 cells, which may be related to PTL’s inhibition of the PI3K/Akt signaling pathway (Bai et al., 2019). After the action of feverfew lactone on human lung cancer A-549 cell line, it induces the change of apoptosis, and it completes the mechanism of apoptosis induction through the death receptor pathway and mitochondrial pathway, and the increase of reactive oxygen species level after the action of feverfew lactone may participate in the mechanism of inducing apoptosis (Lv et al., 2015). Also, *in vivo* studies have found that certain chemical components in the costus have some anti-inflammatory activity. For example, DHC might induce heme oxygenase-1 (HO-1) production in RAW264.7 cells via the p38MAPK/Nrf2 signaling pathway, significantly reduce serum levels of IL1β and TNF-α, and decrease nitric oxide (NO) synthase expression induced by CLP mouse lung tissue; CNL, DHC, acetone extracts significantly reduced the production of inflammatory lung eosinophils, type 2 cytokines, and specific Ig E and mucus in allergic mice (Pyun et al., 2018). The above results indicate that DHC, costunolide, and parthenolide all have anti-lung cancer activity, and DHC also has anti-pneumonia activity. Clinical studies can be conducted on the above ingredients to further demonstrate their efficacy in treating lung diseases.

#### 3.4.5 *Terminalia chebula* Retz., *Terminalia chebula* Retz var. *tomentella* Kurz

A-ru-la (Tibetan: སྦལ་ལ་ནག་པོ།) is the dried ripe fruit of *Terminalia chebula*. and *Terminalia chebula* Retz var. *tomentella*., called Hezi in Chinese, myrobalan in English. The medicine is bitter and astringent in taste, warm in nature. “Moon King Medicine Clinic” said that myrobalan is good for all diseases, generates body temperature, helps digestion, and is used for “Loong”, “Tripa”, “Baekan” and “blood” diseases and the combination of the four diseases. “Jing Zhu Materia Medica” said that myrobalan is the king of all medicines. It is native to India, Myanmar and, other places, and is also distributed in Yunnan, Tibet, Guangdong, Guangxi, and other places in China. As a traditional botanical medicine in China, Chebulan is frequently used in Tibetan medicine and has a variety of pharmacological activities.

The chemical constituents isolated from myrobalan are classified into three main types according to their structure: phenolic acids, tannins, and triterpenes (Yang, 2016). Among them, the phenolic acids include gallic acid and ellagic acid; the tannins include chebulagic acid and chebulinic acid, *etc.*,; the triterpenoids include arjunolic acid, maslinic acid, *etc. In vitro* studies have shown that the pharmacological activities of myrobalan are mainly antitumor activity and antibacterial activity against lung diseases. Tian et al. investigated the effect of aqueous extract of myrobalan on the proliferation of human lung cancer A549 cells and showed that the inhibition rate of human lung cancer A549 cells increased when the concentration of aqueous extract of myrobalan increased, and the mRNA expression of P53 protein increased, so the ability of myrobalan extract to induce apoptosis in A549 cells may be related to the activation of P53 gene (Tian et al., 2015). Bao et al. found that myrobalan extract inhibited the proliferation of lung cancer A549 cells with a significant dose-dependent intensity of action (Bao et al., 2012), and the mechanism may be related to the Tannins prescriptions in myrobalan (Li et al., 2015). Ravi et al. found that the ethanolic extract of myrobalan can be cytotoxic against human breast cancer cell line MCF-7 and lung cancer cell line A-549 with anti-breast and lung cancer activities (Ravi et al., 2016). Lin et al. found that normal doses of chebulinic acid and andrographolide significantly inhibited HSV-1 invasion into human lung cancer cells A549, mainly by inhibiting the interaction of glycosaminoglycans on the surface of A549 cells with the glycoproteins on the surface of HSV-1 virus, and that chebulinic acid and andrographolide were competitive inhibitors of glycosaminoglycans, thus exhibiting some degree of antiviral effect (Lin et al., 2011). In addition, myrobalan has *in vitro* antibacterial activity against *Actinomyces* pleuropneumonia (Kang et al., 2014). At present, only *in vitro* studies have been conducted on the extract of myrobalan, and there is a lack of corresponding *in vivo* and clinical studies to further prove its anti-lung cancer activity.

## 4 Discussion

Tibetan medical theory suggests that Lung diseases are caused by a combination of internal and external factors. The internal causes are the imbalance of the body’s Loong, Tripa, Baekan, and blood. External causes include the invasion of body fluids into the lungs or the consumption of rotten and acidic, overly salty foods, smoking, long-term colds, and overexertion. Tibetan medicine is rich in content and complete in theory. In its long-term practice, Tibetan medicine has accumulated abundant experience in the treatment of lung diseases. It would be very meaningful if these experiences could serve as a reference for the world’s medical community.

These natural plants have a wide range of medicinal sources and are distributed in 77 families. Among them, Rosaceae, Composite, Ericaceae, Leguminosae, and Gentianaceae are more common. Vegetal (medicinal) species from different families have the potential to treat different types of lung diseases. In traditional Tibetan medicine, vegetal (medicinal) species of the Gentianaceae family are often used to treat lung fever and pneumonia. Leguminosae are mostly used for lung fever, pneumonia, and tuberculosis. Rosaceae are commonly used to treat lung fever, pulmonary congestion, and pulmonary tuberculosis. Composite are often used for bronchitis, pneumonia, tracheitis, and pulmonary tuberculosis. Ericaceae are usually used to treat lung abscess and tracheitis. It provides new directions and ideas for pharmacological research. For example, further research is necessary to investigate the pharmacological substance basis and mechanism of action of Gentianaceae (medicinal) species against pneumonia.

This review provides a detailed review of four types of Tibetan medicines commonly used in Tibetan hospitals and the five most frequently used Tibetan medicines in Tibetan prescriptions. We found that these Tibetan medicines (excluding costus, sandalwood, and myrobalan) have the potential to treat a variety of lung diseases. Research has shown that costus, sandalwood, and myrobalan have anti lung cancer effect. Pharmacological research on rhodiola and licorice is relatively sufficient, but further clinical studies are needed to demonstrate their efficacy. In addition, salidroside in rhodiola can treat various lung diseases and may become a candidate drug for treating a variety of lung diseases. However, preclinical studies on other metabolites are yet to be conducted. Similarly, gentian contains numerous metabolites, but only gentiopicroside has been proven to have anti-lung cancer and therapeutic effects on pulmonary fibrosis. Most of the studies were on crude extracts, and there were fewer studies on single pharmacodynamic components. For example, total flavonoids from sea buckthorn have been proven to have anti-lung cancer and chronic bronchitis effects. Although gentian is a multi-original species, pharmacological and clinical studies were focused on *G. szechenyii*. There were no pharmacological studies on the other vegetal (medicinal) species of gentian. Correspondingly, the pharmacological researches on liexiang dujuan are rather for ages and all of them are about R. anthopogonoides.

In addition, this paper has excavated and organized Tibetan medicines and prescriptions for lung diseases, and screened more than five hundred natural medicines, including botanical, animal and mineral medicines. We found that some Tibetan medicines (e.g.,: *G. veitchiorum* Hemsl., *Ephedra saxatilis* Royle ex Florin, *Schisandra sphenanthera* Rehd. et Wils.) and their active ingredients (e.g.,: gentiopicroside, ephedrine, pseudoephedrine, schisandrin B, schisandrin lignan) have good anti-lung cancer, treatment of lung injury, inhibition of pulmonary fibrosis, and anti-pneumonia effects. Gentiopicroside inhibits lung cancer A-549 cell transformation; pseudoephedrine treats lung tissue damage by influenza A virus; ephedrine effectively alleviates LPS-induced apoptosis in alveolar epithelial cells A549, inflammatory damage and oxidative stress (Shao et al., 2021), and reduces lung coefficients in rats with pulmonary fibrosis (Gao, 2010); pseudoephedrine inhibited the process of pulmonary fibrosis and reduced the extent of pulmonary fibrosis (Wei et al., 2017). Schisandra chinensis extract inhibits the growth of the C. pneumoniae strain (CV6) (Hakala et al., 2015). Schisandra chinensis lignans significantly reduced inflammatory damage in lung tissue (Sun et al., 2021). These natural medicines will be potential drug candidates for the treatment of lung diseases and deserve further research and development. However, less than 10% of the Tibetan medicines collated have been studied in modern pharmacology for lung diseases, most of which are *in vitro*, and more *in vivo* studies are needed to confirm their effectiveness in treating lung diseases. More than 90% of the drugs have not been studied for lung disease-related bioactivity. For example, Hong-lian (*Lagotis glauca* Gaertn.) is a commonly used Tibetan medicine for the treatment of lung diseases and was found to be used more frequently based on the TCMISS study. However, to date, no biological activity has been reported for this medicine to lung disease. Similarly, Gun-zhu-mu (*Vitis vinifera* L.); sea buckthorn paste; Ba-ya-ba (*Lancea tibetica* Hook. f. et Thomson); Suo-luo-ga-bao (*Pegaeophylon scapiflorum* (Hook. f. et Thoms.) Marq. et Shaw) also lack studies related to lung disease.

Tibetan medicine has great potential for the treatment of lung diseases. However, there are still some shortcomings in the current research. First of all, most of the studies were *in vitro* and *in vivo*, and there was a lack of relevant clinical studies. Clinical studies of these drugs should be carried out in the future to verify their therapeutic effects on lung diseases. Secondly, most of the studies were on crude extracts, and there were fewer studies on single pharmacodynamic components, as well as studies on pharmacodynamic mechanisms still need to be further explored and expanded. Most of the Tibetan medicines collated in this article still need more research to fill the gaps in their chemical composition and pharmacological activities in pulmonary diseases. Therefore, a systematic study including phytochemical analysis, bioactivity screening, biosynthetic pathway elucidation and conformational relationship studies is necessary, and these findings will provide a more rational basis for the utilization and maximization of the intended therapeutic effects of Tibetan medicines.

Currently, many of the available treatments for lung diseases are of limited efficacy or are related to side effects. The Tibetan medicine compiled in this article might have potential as drug candidates for the development of lung diseases. These treasures urgently need to be excavated using modern scientific methods.

## 5 Conclusion and perspectives

In this review, a total of 586 Tibetan medicines were compiled through literature research of 25 classical works on Tibetan medicine, drug standards, and some Chinese and English databases. Among them, 33 Tibetan medicines have been studied to show their effectiveness in treating lung diseases. Furthermore, using TCMMIS to mine the prescription data, we screened out the five Tibetan medicines with the highest frequency of medication. The ethnopharmacological, phytochemical, and pharmacological properties of these five Tibetan medicines and the four most commonly used Tibetan medicines in Tibetan hospitals were reviewed. According to modern pharmacological research, these Tibetan medicines have a wide range of pharmacological activities against lung diseases. These include activities such as chronic obstructive pulmonary disease, anti-lung cancer, pulmonary fibrosis, lung injury, pneumonia, *etc.*

Chinese ethnomedicine is a potential resource pool for new drug development, and a large amount of experience in new drug development at home and abroad has proven that screening new drugs from ethnomedicine is far more efficient than random screening in modern biomedicine (Zhang and Du, 2021). These Tibetan medicines have valuable experience in the Tibetan Plateau region of China (including Tibet, Qinghai, Gannan Prefecture in Gansu, Ganzi Prefecture and Aba in Sichuan, and Diqing Prefecture in Yunnan). Modern pharmacology studies and clinical studies should be conducted by screening drugs from them to develop new medicines with fewer side effects and better treatment effects, thereby making a significant contribution to the treatment of these diseases on a global scale.

In addition, at present, some Tibetan medicine resources are scarce, such as G. szechenyii, which belongs to the national first-class endangered Tibetan medicine. These endangered Tibetan medicines need to pay more attention to the protection of their medicinal resources while conducting modern pharmacological research. For example, to meet its pharmacological material basis and clinical application research, some measures on appropriate technologies and industrialization models for the regeneration of Tibetan medicine resources and ecological protection should be carried out.

In summary, we can make fuller and more comprehensive use of Tibetan medicines and realize their great potential.
