# Supplementary material for: Traditional Tibetan medicine: therapeutic potential in lung diseases
Source: Front Pharmacol. 2024 Mar 18;15:1365911. doi: 10.3389/fphar.2024.1365911 (PMC10986185; doi:10.3389/fphar.2024.1365911)
Supplement: Supplementary file 1 [file Table1.docx]

Supplementary material

**Traditional Tibetan medicine: therapeutic potential in Lung diseases**

Canlin Li^a^, Yuan Li^b^, Xi Huang^b^, Si Li^b^, Kangzhuo Sangji^a^, Rui Gu^a^*

^a^School of Ethnic Medicine, Chengdu University of Traditional Chinese Medicine, Chengdu, China

^b^School of Pharmacy, Chengdu University of Traditional Chinese Medicine, Chengdu, China

* Correspondence:

Rui Gu

gurui@cdutcm.edu.cn

**TABLE 1 The related information of the 25 monographs referenced in our paper**

| No. | Monograph | Author | The type of author | Ethnic group of author | Data origin of the monograph | Publication date of the monograph | Press of the monograph | Main content of the monograph | Language of the monograph | The type of the monograph |
| --- | --- | --- | --- | --- | --- | --- | --- | --- | --- | --- |
| 1 | Ethnic medicine prescription herbs for prescription preparations | Zhong,G.Y. and Song, M. X. | Scientist and botanist | Han Chinese | Data compilation | 2020 | The People's Health Press | Drug compilation | Chinese | Print edition |
| 2 | Dictionary of Chinese Ethnic Medicine | Jia, M. R. and Zhang, Y. | Botanist and scientist, respectively | Han Chinese | Data compilation | 2016 | China Medical Science and Technology Press | Drug compilation | Chinese | Print edition |
| 3 | Drug Standards of Tibetan Medicine | Chinese Pharmacopoeia Commission | National institution | — | Data compilation | 1995 | Ministry of Health of the People's Republic of China | Pharmacopeia | Chinese | Print edition |
| 4 | Jing Zhu Materia Medica | Dimaer, D. Z. P. C. | Tibetan doctor | Tibetan | Ancient document and ethnobotanical survey | 2012 | Shanghai Science and Technology Press | Drug classification | Chinese version translated by maojizhu | Print edition |
| 5 | Diqing Tibetan Medicine | Yang, J. S. and Chuchen, J. C. | Botanist and Tibetan doctor, respectively | Han Chinese and Tibetan, respectively | Ethnobotanical survey | 1987 | Nationalities Publishing Press of Yunnan | Drug introduction | Chinese | Print edition |
| 6 | National Tibetan Medicine Standard Complete Book(vol. 1) | Wang, B.Q | Scientist and botanist | Han Chinese | Ethnobotanical survey | 2004 | China Medical Electronic Audio and Video press | Drug introduction | Chinese | Print edition |
| 7 | National Tibetan Medicine Standard Complete Book(vol. 2) | Wang, B.Q | Scientist and botanist | Han Chinese | Ethnobotanical survey | 2004 | China Medical Electronic Audio and Video press | Drug introduction | Chinese | Print edition |
| 8 | National Tibetan Medicine Standard Complete Book(vol. 1-3) | Wang, B.Q | Scientist and botanist | Han Chinese | Ethnobotanical survey | 2004 | China Medical Electronic Audio and Video press | Drug introduction | Chinese | Print edition |
| 9 | Moon King Medicine Clinic | Mao,J. Z and Ma S. L. | — |  | Translation Note | 2012 | Shanghai Science and Technology Press | Basic Theory of Tibetan Medicine | Chinese | Print edition |
|  |  |  |  |  |  |  |  |  |  |  |
| 10 | Chinese Tibetan Medicine (vol. 1) | Qinghai Institute for Drug Control | Local institution | — | Ethnobotanical survey | 1996 | Shanghai Science and Technology Press | Drug introduction | Chinese | Print edition |
| 11 | Chinese Tibetan Medicine (vol. 2) | Qinghai Institute for Drug Control | Local institution | — | Ethnobotanical survey | 1996 | Shanghai Science and Technology Press | Drug introduction | Chinese | Print edition |
| 12 | Chinese Tibetan Medicine (vol. 3) | Qinghai Institute for Drug Control | Local institution | — | Ethnobotanical survey | 1996 | Shanghai Science and Technology Press | Drug introduction | Chinese | Print edition |
| 13 | Chinese Herbalism for Tibetan Medicine | Editorial Board of Chinese Herbalism | National institution | — | Data compilation | 2002 | Shanghai Science and Technology Press | Drug compilation | Chinese | Print edition |
| 14 | Chinese Tibetan Materia Medica | Luo, D. S. | Scientist and botanist | Han Chinese | Ethnobotanical survey | 1997 | Ethnic Publishing Press | Drug introduction | Chinese | Print edition |
| 15 | Chinese Ethnic Medicine | Jia, M. R. | Scientist and botanist | Han Chinese | Ethnobotanical survey | 2005 | China Medical Science and Technology Press | Drug introduction | Chinese | Print edition |
| 16 | Tibetan herbal medicine standard in Sichuan Province | Sichuan Provincial Drug Administration | Local institution | — | Experimental Research | 2020 | Sichuan Science and Technology Press | Pharmacopeia | Chinese | Print edition |
| 17 | Tibetan Medicine Standards(vol. 1) | Health Bureau of Tibet, Qinghai, Sichuan, Gansu, Yunnan, and Xinjiang | Local institution | — | Data compilation | 1979 | Qinghai People’s Publishing Press | Pharmacopeia | Chinese | Print edition |
| 18 | Tibetan Medicine Standards(vol. 2) | Health Bureau of Tibet, Qinghai, Sichuan, Gansu, Yunnan, and Xinjiang | Local institution | — | Data compilation | 1979 | Qinghai People’s Publishing Press | Pharmacopeia | Chinese | Print edition |
| 19 | Tibetan Medicine Journal | Editorial Committee of Tibetan Medicine Journal, Northwest Plateau Institute of Biology, Chinese Academy of Sciences | Scientist and botanist | — | Data compilation | 1991 | Qinghai People’s Publishing Press | Drug introduction | Chinese | Print edition |
| 20 | Medicine Four Continues | Yutuo, Y. D. G.B | Tibetan doctor | Tibetan | Ancient document and ethnobotanical survey | 2012 | Shanghai Science and Technology Press | Tibetan Medical Theory and Treatment | Chinese | Print edition |
| 21 | Yutuo Herbal | Qianyutuo, Y. D. G. B | Tibetan doctor | Tibetan | Ancient document | 2016 | Qinghai People's Publishing Press | Drug compilation | Chinese | Print edition |
| 22 | Dumu Herbal | Xiwacuo | Buddhist | Ancient Indians | Ancient document | 2016 | Qinghai People's Publishing Press | Drug compilation | Chinese | Print edition |
| 23 | Miaoyin Herbal | Bairuozana | The Great Tibetan Interpreter | Tibetan | Ancient document | 2016 | Qinghai People's Publishing Press | Drug compilation | Chinese | Print edition |
| 24 | The Sea of Drug Names | Gema, R. Q. D.J | Living Buddha | Tibetan | Ancient document | 2016 | Qinghai People's Publishing Press | Drug compilation | Chinese | Print edition |
| 25 | Leiwuqi County. Chinese and Tibetan Medicine Resources | Wei, Y. S., Zha, X. D. W., and Li, Y. | Scientist and botanist | Han Chinese | Ethnobotanical survey | 2020 | China Textile Publishing House | Drug compilation | Chinese | Print edition |

**TABLE 2 Natural Botanical Medicines for treating pulmonary diseases in the Traditional Tibetan medical system**

| No. | Latin name | Tibetan name | Family | Life from | Used part | Treated pulmonary diseases |
| --- | --- | --- | --- | --- | --- | --- |
| 1 | （*Gentiana algida*Pall.） | Bang-jian-ga-bao | Gentianaceae | Herb | Dried flower | Cough with lung heat, cough from pneumonia (Zhong and Song, 2020) |
| 2 | (*Gentiana flavomaculata* Hayata) | Bang-jian-ga-bao | Gentianaceae | Herb | Dried flower | Cough with lung heat (Zhong and Song, 2020) |
| 3 | (*Gentiana szechenyii* Kanitz.) | Bang-jian-ga-bao | Gentianaceae | Herb | Dried flower | Cough with lung heat (Dimaer, 2012) |
| 4 | (*Gentiana purdomii*Marq.) | Bang-jian-ga-bao | Gentianaceae | Herb | Dried flower | Cough with lung heat (Yutuo, 2012) |
| 5 | *Gentiana altorum* H. Smith ex Marq. | Bang-jian-en-bao | Gentianaceae | Herb | Dried flower | Lung heat (Control, 1996) |
| 6 | *Gentiana arethusae* var. Delicatula Marq. | Bang-jian-en-bao | Gentianaceae | Herb | Dried flower | Lung heat (Jia and Zhang, 2016) |
| 7 | *Gentiana aristata* Maxim. | Bang-jian-en-bao | Gentianaceae | Herb | Dried flower | Cough with lung heat (Jia and Zhang, 2016) |
| 8 | *Gentiana atuntsiensis* W. W. Smith | Bang-jian-en-bao | Gentianaceae | Herb | Dried flower | Lung heat (Jia and Zhang, 2016) |
| 9 | *Gentiana capitata* Buch-Ham ex D. Don | Wan-bu | Gentianaceae | Herb | Dried flower | Lung heat (Jia and Zhang, 2016) |
| 10 | *Gentiana lawrencei* var. farreri T. N. Ho | Bang-jian-en-bao | Gentianaceae | Herb | Dried flower | Lung heat (Jia and Zhang, 2016) |
| 11 | *Gentiana filistyla*Balf. F. et Forrest ex Marq. | Bang-jian-en-bao | Gentianaceae | Herb | Dried flower | Lung heat, lun diseases (Jia and Zhang, 2016) |
| 12 | *Gentiana futtereri* Diels et Gilg | Ji-jie-mo-bao | Gentianaceae | Herb | Whole plant with flower | Emphysema, cough with lung heat, pneumonia (Jia, 2005) |
| 13 | *Gentiana erectosepala* T. N. Ho | Ji-jie-en-bao | Gentianaceae | Herb | Whole plant with flower | Lung heat (Jia and Zhang, 2016) |
| 14 | *Gentiana macrophylla* Pall. | Ji-jie-na-bao | Gentianaceae | Herb | Whole plant with flower | Fever frome, lun heat (Jia and Zhang, 2016) |
| 15 | *Gentiana nubigena* Edgew. | Bang-jian-ga-bao | Gentianaceae | Herb | Flower; root; leaf | Lung heat, cough frome pneumonia;cough with lung heat;emphysema (Jia, 2005) |
| 16 | *Gentiana robusta* King ex J. D.  Hook. f. | Ji-jie-ga-bao | Gentianaceae | Herb | Flower ;root | Lung heat, pulmonary tuberculosis (Jia and Zhang, 2016) |
| 17 | *Gentiana sino-ornata* Balf. f. | Bang-jian-cha-bao | Gentianaceae | Herb | Flower | Lung heat, cough with lung heat (Jia and Zhang, 2016) |
| 18 | *Gentiana sino- ornata* var. glorisoa Maxq. | Bang-jian-ga-bao | Gentianaceae | Herb | Flower | Lung heat, cough with lung heat (Jia and Zhang, 2016) |
| 19 | *Gentiana stipitata* Edgew. | Bang-jian-cha-bao | Gentianaceae | Herb | Flower | Lung heat (Administration, 2020) |
| 20 | *Gentiana straminea* Maxim. | Ji-jie-ga-bao | Gentianaceae | Herb | Whole plant | Fever frome lun disease (Ma, 2012) |
| 21 | *Gentiana tibetica* King ex Hook. f. | Ji-jie-na-bao | Gentianaceae | Herb | Whole plant | Pulmonary tuberculosis (Ma, 2012) |
| 22 | *Gentiana veitchiorum* Hemsl. | Bang-jian-en-bao | Gentianaceae | Herb | Flower; root; rhizome | Lung heat, cough with lung heat (Luo, 1997) |
| 23 | *Gentiana waltonii* Burk*.* | Ji-jie-ma-bao | Gentianaceae | Herb | Root | Pulmonary tuberculosis (Jia and Zhang, 2016) |
| 24 | *Gentiana yunnanensis* Franch. | Bang-jian-ga-bao | Gentianaceae | Herb | Flower | Lung heat (Jia and Zhang, 2016) |
| 25 | *Gentianella arenaria* (Maxim.)  T. N. Ho |  | Gentianaceae | Herb | Flower | Pulmonary dryness (Jia and Zhang, 2016) |
| 26 | *Gentianopsis barbata*  (Froel.) Ma | Jia-di | Gentianaceae | Herb | Whole plant | Pneumonia (Jia and Zhang, 2016) |
| 27 | *Gentianopsis grandis* Ma | Jia-di | Gentianaceae | Herb | Whole plant | Pneumonia (Jia and Zhang, 2016) |
| 28 | *Gentianopsis paludosa* Ma | Jia-di | Gentianaceae | Herb | Whole plant | Pneumonia (Jia and Zhang, 2016) |
| 29 | *Cupressus torulosa* D. Don | Xiu-ba | Cupressaceae | Tree | Fruit; branches; leaves | Lung disease (Luo, 1997) |
| 30 | （*Platycladus orientalis*(L.)Franco.） | Xiu-ba | Cupressaceae | Tree | Branches; leaves;kernel | Lung disease (Luo, 1997) |
| 31 | *Juniperus chinensis* L. | Xiu-ba | Cupressaceae | Tree | Strobile; branches; leaves;bark | Pneumonia, Pulmonary tuberculosis (Bairuozana, 2016) |
| 32 | *Juniperus convallium* Rehder et E. H. Wilson | Xiu-ba | Cupressaceae | Tree | Branches; leaves | Lung heat (Jia, 2005) |
| 33 | Cupressus torulosa D. Don | Xiu-ba | Cupressaceae | Tree | Fruit; banranches ; leaves | Lung disease (Jia, 2005) |
| 34 | *Plantago asiatica* L. | Ta-ran-mu | Plantaginaceae | Herb | Whole palnt | Pneumonia (Jia, 2005) |
| 35 | *Plantago depressant* Willd. | Ta-ran-mu | Plantaginaceae | Herb | Whole palnt | Pneumonia (Jia, 2005) |
| 36 | *Plantago major* L. | Ta-ran-mu | Plantaginaceae | Herb | Whole palnt | Pneumonia (Zhong and Song, 2020) |
| 37 | *Juglans regia* L. | Da-ga | Juglandaceae | Tree | Dried and ripe seeds | Pulmonary dryness (Zhong and Song, 2020) |
| 38 | *Terminaalia chebula* Retz. | A-ru-la | Combretaceae | Tree | Dried and ripe fruit | Astringing lun for relieving cough (Zhong and Song, 2020) |
| 39 | *Terminaalia chebula* Retz var. *tomentella* Kurz | A-ru-la | Combretaceae | Tree | Dried and ripe fruit | Astringing lun for relieving cough (Zhong and Song, 2020) |
| 40 | *Ephedra intermedia*Schrenk ex Mey. | Ce-dun-mu | Ephedraceae | Herb | Dried grassstem | Bronchial asthma (Zhong and Song, 2020) |
| 41 | *Ephedra sinica* Stapf | Ce-dun-mu | Ephedraceae | Herb | Dried grassstem | Bronchial asthma (Zhong and Song, 2020) |
| 42 | *Ephedra eqqusetina* Bunge. | Ce-dun-mu | Ephedraceae | Herb | Dried grassstem | Bronchial asthma (Zhong and Song, 2020) |
| 43 | *Ephedra saxatilis*Royle ex Florin | Ce-dun-mu | Ephedraceae | Herb | Dried grassstem | Bronchial asthma (Zhong and Song, 2020) |
| 44 | *Bombax ceiba*Linnaeus | Na-ga-ge-sa | bombacaceae | Tree | Dried flower | Lun heat (Jia, 2005) |
| 45 | *Schisandra sphenanthera* Rehd. et Wils. | Da-zhe-he | Magnoliaceae | Vine | Dried ripe fruit | Maintenance of lungs (Zhong and Song, 2020) |
| 46 | *Symplocos paniculata* (Thunb.) Miq. | Xi-kan | alaceae | Tree | Dried leaves | Lung heat (Jia, 2005) |
| 47 | *Cyperus rotundus* L. | La-gang | cyperaceae | Herb | Dried rhizome | Tracheitis, lung heat (Ma, 2012) |
| 48 | *Houttuynia cordata* Thunb. | Nie-zhi-zhuo-wei-ao | Saururaceae | Herb | Fresh whole grass or dried ground parts | Abscess of lung; pneumonia, pulmonary tuberculosis (Luo, 1997) |
| 49 | *Onosma hookeri* Clarke var. *longiflorum* Duthie ex Stapf | Zhe-mo | Boraginaceae | Herb | Dried root or root cork | Pneumonia (Commission, 1995) |
| 50 | *Onosma hookeri* Clarke | Zhe-mo | Boraginaceae | Herb | Dried root or root cork | Pneumonia (Jia, 2005) |
| 51 | *Onosma exsertum* Hemsl. | Zhe-mo | Boraginaceae | Herb | Dried root or root cork | Pneumonia, pulmonary tuberculosi, Abscess of lun (Zhong and Song, 2020) |
| 52 | *Onosma multiramosum* Hand.-Mazz. | Zhe-mo | Boraginaceae | Herb | Dried root or root cork | Pneumonia, pulmonary tuberculosi, Abscess of lung (Yang, 1987) |
| 53 | *Onosma confertum* W. W. Smith | Zhe-mo | Boraginaceae | Herb | Dried root or root cork | Pneumonia, pulmonary tuberculosi, abscess of lung (Wang, 2004) |
| 54 | *Onosma panicul*atum Bur. et Franch. | Zhe-mo | Boraginaceae | Herb | Dried root or root cork | Pneumonia (Jia, 2005) |
| 55 | *Lithospermum erythrorhizon*Sieb. et Zucc. | Zhe-mo | Boraginaceae | Herb | Dried root | Abscess of lung;cough with lung heat (Xiwacuo, 2016) |
| 56 | *Arnebia euchroma* (Royle)Johnst. | Zhe-mo | Boraginaceae | Herb | Dried root | Abscess of lung;cough with lung heat (Jia and Zhang, 2016) |
| 57 | *Arnebia guttata* Bunge | Zhe-mo | Boraginaceae | Herb | Dried root | Abscess of lung;cough with lun heat (Zhong and Song, 2020) |
| 58 | *Vincetoxicum forrestii* (Schltr.) C. Y. Wu et D. Z. LiAscle |  | Piadaceae | Herb | Seed and whole plant | cough with lung heat(Herbalism, 2002) |
| 59 | *Abies delavayi*Franch. | Tang-zhe | Pinaceae | Tree | Strobile | Lung disease (Control, 1996) |
| 60 | Abies aquamata Mast. | Tang-zhe | Pinaceae | Tree | Strobile | Lung disease (Jia and Zhang, 2016) |
| 61 | *Picea crassifolia* Kom. | Tang-cha-he | Pinaceae | Tree | Strobile | Lung disease (Control, 1996) |
| 62 | *Picea likiangensis* var. *rubescens*Rehder & E. H. Wilson | Tang-cha-he | Pinaceae | Tree | Strobile | Lung disease (Control, 1996) |
| 63 | *Picea purpurea* Mast. | Tang-cha-he | Pinaceae | Tree | Strobile | Lung disease (Control, 1996) |
| 64 | *Picea smithiana* (Wall.)  Boiss. | Tang-cha-he | Pinaceae | Tree | Strobile | Lung disease (Control, 1996) |
| 65 | *Pinus armandi* Franch. | Tang-xing | Pinaceae | Tree | Strobile | Tracheitis (Jia, 2005) |
| 65 | *Pinus densata* Mast. | Tang-xing | Pinaceae | Tree | Strobile | Tracheitis (Jia and Zhang, 2016) |
| 67 | *Pinus wallichiana*A. B. Jackson | Tang-xing | Pinaceae | Tree | Strobile | Tracheitis (Jia, 2005) |
| 68 | *Pinus massoniana* Lamb. | Zhong-xiang | Pinaceae | Tree | Strobile | Lung disease (Jia, 2005) |
| 69 | *Pinus yunnanensis* Franch. | Zhong-xiang | Pinaceae | Tree | Strobile | Lung disease (Jia, 2005) |
| 70 | *Themopsis barbata* Benth. | La-wa-se-ma | Leguminosae | Tree | Dried root | Lung heat (Jia, 2005) |
| 71 | *Pterocarpus indicus*willd. | Zan-dan-ma-bu | Leguminosae | Tree | Heartwood | Pneumonia, lung sbscess (Luo, 1997) |
| 72 | *Medicago ruthenica*(L.) Trautv. | Bu-su-hang | Leguminosae | Herb | Dried whole plant | Cough with lung heat (Control, 1996) |
| 73 | *Medicago lupulina* L. | Bu-su-hang | Leguminosae | Herb | Dried whole plant | Cough with lun heat (Ma, 2012) |
| 74 | *Glycyrrhiza uralensis* Fisch | Xiang-an | Leguminosae | Herb | Dried roots and rhizomes | Lung disease (Xiwacuo, 2016) |
| 75 | *Glycyrrhiza inflata* Batal. | Xiang-an | Leguminosae | Herb | Dried roots and rhizomes | Lung disease (Jia, 2005) |
| 76 | *Glycyrrhiza glabra* L. | Xiang-an | Leguminosae | Herb | Dried roots and rhizomes | Lung disease (Jia, 2005) |
| 77 | *Oxytropis kansuensis* Bunge | Sai-ga-er | Leguminosae | Herb | Dried flower | Lung heat (Luo, 1997) |
| 78 | *Oxytropis ochrocephala* Bunge | Sai-ga-er | Leguminosae | Herb | Dried flower | Lung heat (Luo, 1997) |
| 79 | *Senegalia catechu*(L. f.) P. J. H. Hurter & Mabb*.* | Dui-jia | Leguminosae | tree | Extactum; Heartwood | Hemoptysis in tuberculosis, cough with lung heat (Jia, 2005) |
| 80 | *Caragana franchetiana* Kom. | Zha-ma | Leguminosae | tree | Flower | Cough of pulmonary furuncles (Jia and Zhang, 2016) |
| 81 | *Caragana kozlowii* Kom. | Zha-ma | Leguminosae | tree | Stem; bark | Cough of pulmonary furuncles (Jia and Zhang, 2016) |
| 82 | *Caragana junatovii*Gorbunova | Zha-ma | Leguminosae | tree | Stem; bark | Cough of pulmonary furuncles (Jia and Zhang, 2016) |
| 83 | *Caragana spinosa*(L.) DC. | Zha-ma | Leguminosae | tree | Stem; bark | Cough of pulmonary furuncles (Jia, 2005) |
| 84 | *Lathyrus pratensis*L. | Jia-shan | Leguminosae | Herb | Whole plant | Pneumonia (Jia and Zhang, 2016) |
| 85 | *Lathyrus quinquenervius* (Miq.)  Litv. | Jia-shan | Leguminosae | Herb | Whole plant | Pneumonia (Jia and Zhang, 2016) |
| 86 | *Medicago sativa*L. |  | Leguminosae | Herb | Whole plant | Lung heat (Jia and Zhang, 2016) |
| 87 | *Oxytropis chiliophylla* Royle | E-da-xia | Leguminosae | Herb | Whole plant | Pulmonary cough and wheezing (Control, 1996) |
| 88 | *Oxytropis falcata* Bunge | E-da-xia | Leguminosae | Herb | Whole plant | Pulmonary cough and wheezing (Control, 1996) |
| 89 | *Oxytropis melanocalyx* Bunge | Sai-wan | Leguminosae | Herb | Whole plant | Lung heat (Jia and Zhang, 2016) |
| 90 | *Oxytropis ochrocephala* Bunge | Sai-ga-er | Leguminosae | Herb | Whole plant | Cough with lung heat (Jia, 2005) |
| 91 | *Sophora flavescens* Ait. | Le-zhe | Leguminosae | Herb | Stem | Lung disease (Jia and Zhang, 2016) |
| 92 | *Trigonella foenum -graecum* L. | Xu-mu-sa | Leguminosae | Herb | Seeds; fruit | Seed for lung abscess, fruit for pulmonary pus (Luo, 1997) |
| 93 | *Vicia cracca* L. | Xi-wu-sai | Leguminosae | Herb | Whole palnt | Cough with lung heat (Jia and Zhang, 2016) |
| 94 | *Vicia multicaulis* Ledeb. | Cuo-ma-ke-de | Leguminosae | Herb | Whole palnt | Cough with lung heat (Jia and Zhang, 2016) |
| 95 | *Vicia nummularia* Hand. -Mazz.. | Jia-shan | Leguminosae | Herb | Whole palnt | Pneumonia (Jia and Zhang, 2016) |
| 96 | *Vicia tibetica* Prain ex C. A. C. Fisch. | Cuo-ma-ke-de | Leguminosae | Herb | Whole palnt | Cough with lung heat (Jia and Zhang, 2016) |
| 97 | *Astragalus yunnanensis* Franch. | Sai-en | Leguminosae | Herb | Whole palnt | Lung heat (Jia and Zhang, 2016) |
| 98 | *Astragalus floridulus* Podlech | Sa-sai-er | Leguminosae | Herb | Whole palnt | Lung heat (Jia and Zhang, 2016) |
| 99 | *Mucuna birdwoodiana* Tutcher | La-guo-xiao-xia | Leguminosae | Vine | Dried seeds | Lung disease (Control, 1996) |
| 100 | *Aconitum brevicalcaratum*(Finet et Gagnep.) Diels | Ka-pu-de-luo | Ranunculaceae | herb | Root | Pandemic fever; cough with lung heat (Luo, 1997) |
| 101 | *Aconitum naviculare* (Bruhl.) Stapf | Pang-a-ga-bao | Ranunculaceae | herb | Dried whole plan | Lung heat (Ma, 2012) |
| 102 | *Gymnaconitum gymnandrum* (Maxim.) Wei Wang & Z. D. Chen | Zheng-ba-da-che | Ranunculaceae | herb | Dried whole plan | Lung heat (Control, 1996) |
| 103 | *Aconitum tanguticum*(Maxim.) Stapf | Bang-ga | Ranunculaceae | herb | Dried whole plan | Lung heat (Jia, 2005) |
| 104 | *Anemone obtusiloba* D. Don | Su-ga | Ranunculaceae | Herb | Abovegroundpart; root; fruit; flower | Chronic bronchitis, chronic tracheitis (Jia and Zhang, 2016) |
| 105 | *Anemone rivularis* Buch.-Ham. | Su-ga | Ranunculaceae | Herb | Abovegroundpart; root; fruit; flower | Chronic bronchitis, chronic tracheitis (Jia and Zhang, 2016) |
| 106 | *Anemone trullifolia* Hook. f. et Thoms. | Bu-er-qing | Ranunculaceae | herb | Root; flower | Chronic bronchitis (Jia and Zhang, 2016) |
| 107 | *Anemone trullifolia* Hook. f. et Thoms. | Ran-su | Ranunculaceae | herb | Root | Chronic tracheitis (Jia and Zhang, 2016) |
| 108 | *Callianthemum pimpinelloides* Hook. f. et Thoms. | Rao-bao-jue-jie | Ranunculaceae | herb | Whole plant | Cough from pneumonia (Ma, 2012) |
| 109 | *Delphinium forrestii* Diels. | Ka-pu-de-luo | Ranunculaceae | Herb | Whole plant | Cough with lung heat (Luo, 1997) |
| 110 | *Delphinium kamaonense* var.*glabrescens* W. T. Wang | Xia-gang-wa | Ranunculaceae | Herb | Root | Lung heat (Jia and Zhang, 2016) |
| 111 | *Delphinium trichophorum* Franch. | Ka-pu-de-luo | Ranunculaceae | Herb | Aboveground part | Pandemic fever, cough with lung heat (Luo, 1997) |
| 112 | *Delphinium yunnanense* Franch. | Nuo-du-xi-li | Ranunculaceae | Herb | Root | Pulmonary tuberculosis (Jia and Zhang, 2016) |
| 113 | *Delphinium pseudopulcherrimum* W.T.Wang | Ke-bu-di-lu | Ranunculaceae | Herb | Aboveground part | Lung heat (Ma, 2012) |
| 114 | *Thalictrum petaloideum* L. | Zhu-ga-man-ba | Ranunculaceae | Herb | Root; rhizome; fruit | Pneumonia (Luo, 1997) |
| 115 | *Adenophora lilifolioides* Pax. et Hoffm. | Lu-dui-duo-ji-men-ba | Campanulaceae | Herb | Whole palnt | Lung disease (Health Bureau of Tibet, 2019) |
| 116 | *Adenophora stenanthina* (Ledeb.) Kitagawa. | Le-duo-dao-ji-man-ba | Campanulaceae | herb | Root | Tracheitis cough with lung heat; cough due to deficiency of the lung (Jia and Zhang, 2016) |
| 117 | *Codonopsis convolvulacea* Kurz | Ni-wa | Campanulaceae | herb | Root | Cough of pulmonary furuncles (Yang, 1987) |
| 118 | *Pseudocodon convolvulaceus*subsp*. forrestii* (Diels) D.Y.Hong | Nie-wa | Campanulaceae | herb | Root | Lung disease (Jia and Zhang, 2016) |
| 119 | salvia miltilrrhiza Bunge | Ji-zi-mo-bo | Labiatae | Herb | Root; rhizome | Hemoptysis of the lungs (Luo, 1997) |
| 120 | *Eriophyton wallichii* Behth | Bang-yi-bu-ru | Labiatae | Herb | Dried whole plant | Pneumonia, emphysema, pulmonary tuberculosis, cough with lung heat (Luo, 1997) |
| 121 | *Phlomoides betonicoides*(Diels) Kamelin et Makhm. | Lou-mo-er | Labiatae | Herb | Root | Lung disease (Luo, 1997) |
| 122 | *Phlomoides medicinalis* (Diels) Kamelin et Makhm | Lou-mo-er | Labiatae | Herb | Root | Lung disease (Luo, 1997) |
| 123 | *Marmoritis complanata*(Dunn) A. L. Budantzev | Nian-du-ba | Labiatae | Herb | Leaf | Cough with lung heat, pulmonary abscess, pulmonary tuberculosis, pneumonia (Ma, 2012) |
| 124 | phyllophyton tibeticum (Jacq.) C. Y. Wu | Bang-shen-bu-rou | Labiatae | Herb | Leaf | Pulmonary abscess, pulmonary tuberculosis, pneumonia (Jia, 2005) |
| 125 | *Ajuga lupulina* Maxim. | Sen-di | Labiatae | herb | Whole plant | Tracheitis (Jia and Zhang, 2016) |
| 126 | *Phlomis younghusbandii* Mukerjee | Lu-mu-er | Labiatae | Herb | Dried root | Bronchitis (Control, 1996) |
| 127 | *Salvia glutinosa* L. | Lu-mu-er | Labiatae | Herb | Root | Hemoptysis of the lungs (Jia, 2005) |
| 128 | *Salvia prattii* Hemsl. | Ji-zi-qing-mo | Labiatae | Herb | Root | Hemoptysis of the lung (Jia and Zhang, 2016) |
| 129 | *Salvia przewalskii* Maxim. | Ji-zi-mu-bao | Labiatae | Herb | Flower; root | Lung heat, pneumonia, hemoptysis in tuberculosis (Luo, 1997) |
| 130 | *Salvia przewalskii* var. *mandarinorum* (Diels) Stib | Ji-zi-en-bao | Labiatae | Herb | Root | Hemoptysis of the lungs (Jia, 2005) |
| 131 | *Salvia roborowskii* Maxim. | Ji-zi-ga-bao | Labiatae | Herb | Root | Hemoptysis of the lungs (Luo, 1997) |
| 132 | *Malus transitoria* (Batal.)Schneidd. | Ao-se-zhe-bu | Rosaceae | Shrub | Fruit | Lung disease (Ma, 2012) |
| 133 | *Rubus saxatilis* L. | Ga-zha | Rosaceae | Shrub | Dried stem | Lung disease (Zhong and Song, 2020) |
| 134 | *Rubus phoenicolasius* Maxim. | Ga-zha | Rosaceae | Shrub | Dried stem | Lung disease (Control, 1996) |
| 135 | *Rubus sachalinensis* Levl. | Ga-zha | Rosaceae | Shrub | Dried stem | Lung disease (Zhong and Song, 2020) |
| 136 | *Fragaria orientalls* Lozinsk. | Zi-zi-sa-zeng | Rosaceae | Herb | Dried whole plant | Pulmonary venous pleonaemia; pulmonary sputum (Jia, 2005) |
| 137 | *Rosa omeiensis* Rolfe | Sai-wa | Rosaceae | Herb | Dried flower | Cough with lung heat (Jia, 2005) |
| 138 | *Rosa omeiensis* Rolfe | Sai-wa | Rosaceae | Herb | Dried flower | Cough with lung heat (Jia, 2005) |
| 139 | *Rosa multiflora* Thunb. | Sai-wa | Rosaceae | Herb | Dried flower | Cough with lung heat (Jia, 2005) |
| 140 | *Rosa rugosa* Thunb. | Sai-wa | Rosaceae | Herb | Dried flower | Cough with lung heat (Zhong and Song, 2020) |
| 141 | *Geum aleppicum* Jacq. | Lan-bu-zheng | Rosaceae | Herb | Dried whole plant | Atrophic (Control, 1996) |
| 142 | *Chaenomeles speciosa* Nakai | Sai-ya | Rosaceae | Tree | Fruit | Inhibited lung qi (Jia and Zhang, 2016) |
| 143 | Fragaria ananassa Duch. | Zi-zi-sa-zeng | Rosaceae | Herb | Dried whole plant | Pulmonary venous pleonaemia (Wang, 2004) |
| 144 | *Fragaria gracilis* Losinsk. | Zi-zi-sa-zeng | Rosaceae | Herb | Dried whole plant | Pulmonary tuberculosis, abscess of lung (Jia and Zhang, 2016) |
| 145 | *Fragaria moupinensis*(Franch.) Card. | Zi-zi-sa-zeng | Rosaceae | Herb | Dried whole plant | Pulmonary tuberculosis, abscess of lung, pneumonia, Pulmonary venous pleonaemia (Wei et al., 2020) |
| 146 | *Fragaria nilgerrensis* Schltdl. ex Gay | Zi-zi-sa-zeng | Rosaceae | Herb | Dried whole plant | Pulmonary tuberculosis, abscess of lung, pneumonia, Pulmonary venous pleonaemia (Jia, 2005) |
| 147 | *Fragaria nubicola* Lindl.ex Lacaita | Zi-zi-sa-zeng | Rosaceae | Herb | Dried whole plant | Pulmonary tuberculosis, abscess of lung, Pulmonary venous pleonaemia (Ma, 2012) |
| 148 | *Fragaria vesca* L. | Zi-zi-sa-zeng | Rosaceae | Herb | Dried whole plant | Pulmonary tuberculosis(Jia, 2005) |
| 149 | *Dasiphora fruticosa*(L.) Rydb. | Ben-na | Rosaceae | Shrub | Flower | Lung disease (Yang, 1987) |
| 150 | *Dasiphora arbuscula*(D. Don) Soják | Ben-na | Rosaceae | Shrub | Flower | Lung disease (Jia and Zhang, 2016) |
| 151 | *Dasiphora glabra*(G. Lodd.) Soják | Ben-na | Rosaceae | Shrub | Flower; leaf | Lung disease (Luo, 1997) |
| 152 | *Potentilla reptans L.* var. *sericophylla* Franch. | Zi-zi-sa-zeng | Rosaceae | Herb | Dried whole plant | Pulmonary venous pleonaemia (Jia and Zhang, 2016) |
| 153 | *Rosa graciliflora* Rehd. et Wils. | Se-wa | Rosaceae | Tree | flower | Cough with lung heat (Jia and Zhang, 2016) |
| 154 | *Rosa koreane* Kom. | Se-wa | Rosaceae | Tree | flower | Cough with lung hea (Jia and Zhang, 2016) |
| 155 | *Rosa laevigata* Michx. | Se-rong | Rosaceae | Tree | Flower; fruit | Cough due to deficiency of the lung (Jia and Zhang, 2016) |
| 156 | *Rosa mairei*Lévl. | Se-wei-mei-duo | Rosaceae | Tree | flower | Cough with lung heat (Jia and Zhang, 2016) |
| 157 | *Rosa moyesii* Hemsl. | Se-yong | Rosaceae | Tree | flower | Cough with lung heat (Jia and Zhang, 2016) |
| 158 | *Rosa omeiensis* f. *pteracantha* Rhed. et Wils. | Se-wa | Rosaceae | Tree | flower | Cough with lung heat (Jia and Zhang, 2016) |
| 159 | *Rosa primula* Bouleng. | Sai-wei-mei-duo | Rosaceae | Tree | flower | Cough with lung heat (Jia and Zhang, 2016) |
| 160 | *Rosa platyacantha*Schrenk | Se-wei-mei-duo | Rosaceae | Tree | flower | Cough with lung heat (Jia and Zhang, 2016) |
| 161 | *Rosa sikangensis* Yu et Ku | Se-wa | Rosaceae | Tree | flower | Cough with lung heat (Jia and Zhang, 2016) |
| 162 | *Rosa taronensi*s Yu et Ku | Sai-guo | Rosaceae | Tree | flower | Cough with lung heat (Jia and Zhang, 2016) |
| 163 | Rosa webbiana Wall. ex Royle | Sai-guo | Rosaceae | Tree | flower | Cough with lung heat (Jia and Zhang, 2016) |
| 164 | *Rubus amabilis* Focke | Gan-da-ga-ri | Rosaceae | Tree | Remove the peel of stem and branches | Cough with lung heat (Jia, 2005) |
| 165 | *Rubus amabilis* Focke | Ga-zha-ga-ri | Rosaceae | Tree | Remove the peel of stem | Cough with lung heat (Jia and Zhang, 2016) |
| 166 | *Rubus corchorifolius* L. f. | Gan-zha-ga-ri | Rosaceae | Tree | Root; fruit | Lung disease (Jia and Zhang, 2016) |
| 167 | *Rubus hypopitys* Focke | Gan-zha-ya-ga | Rosaceae | shrub | Aboveground part or fruit | Lung disease (Jia and Zhang, 2016) |
| 168 | *Rubus irritans* Focke | Gan-da-ga-ri | Rosaceae | Shrub | Remove the peel of stem and branches | Cough with lung heat (Ma, 2012) |
| 169 | *Rubus maershanensis*Huang C. Wang et H. Sun | Gen-zha-ga-ri | Rosaceae | shrub | Aboveground part or fruit | Lung disease (Jia and Zhang, 2016) |
| 170 | *Rubus niveus* Thunb. | Gen-zha-ga-ri | Rosaceae | shrub | Stem; branches | Cough with lung heat (Yang, 1987) |
| 171 | Rubus stans Focke | Ga-zha-ga-ri | Rosaceae | Shrub | Remove the peel of stem | Cough with lung heat (Jia and Zhang, 2016) |
| 172 | *Rubus subomatus* var. *melandenus* Focke | Gan-zha-ga-re | Rosaceae | Shrub | Whole plant | Lung disease (Control, 1996) |
| 173 | *Rubus sumatranus* Miq. | Gen-zha-ga-ren | Rosaceae | Shrub | Stem; barnches | Cough with lung heat (Jia and Zhang, 2016) |
| 174 | *Sibbaldia adpressa* Bunge | Tong-bao-jiu-mu-ji | Rosaceae | Herb | Whple plant | Cough with lung heat, pulmonary tuberculosis, pulmonary abscess, lun disease (Jia and Zhang, 2016) |
| 175 | *Sibbaldia pentaphylla* J. Krause | Tong-bao-jiu-mu-ji | Rosaceae | Herb | Whple plant | Cough with lung heat, pulmonary tuberculosis, pulmonary abscess, lun disease (Jia and Zhang, 2016) |
| 176 | *Sibbaldia aphanopetala*Hand.-Mazz | Tong-bao-jiu-mu-ji | Rosaceae | Herb | Whple plant | Cough with lung heat, pulmonary tuberculosis, pulmonary abscess, lun disease, cough from pneumonia |
| 177 | *Sibbaldia purpurea* Royle | Tong-bao-jiu-mu-ji | Rosaceae | Herb | Whple plant | Cough with lung heat, pulmonary tuberculosis; pulmonary abscess; lun disease (Jia and Zhang, 2016) |
| 178 | Spiraea alpina Pall. | Ma-hei | Rosaceae | Herb | Flower; leaf | Pulmonary venous pleonaemia (Wang, 2004) |
| 179 | *Spiraea bella* Sims. | Ma-xi | Rosaceae | Herb | Flower; leaf; epicormic branch | Pulmonary venous pleonaemia (Jia and Zhang, 2016) |
| 180 | *Spiraea lasiocarpa* Karelin et Kirilov | Ma-xi | Rosaceae | Herb | Flower | Pulmonary venous pleonaemia (Luo, 1997) |
| 181 | *Spiraea myrtilloides* Rehd. | Ma-xie | Rosaceae | Herb | Root | Pulmonary venous pleonaemia (Zhong and Song, 2020) |
| 182 | *Spiraea schneideriana* Rehd. | Ma-xie | Rosacea | Herb | Flower; leaf; root | Pulmonary venous pleonaemia (Zhong and Song, 2020) |
| 183 | Potentilla parvifolia Fisch. Apud Lehm. Var. Armerioides(Hook. f.) Yu et Li | Ban-ma | Rosacea | Herb | Flower; leaf | Lung disease (Ma, 2012) |
| 184 | *Rubia manjin* Roxb. ex Flem. | Zuo-xin-ba | Rubiaceae | Herb | Whole plant | Lung heat, pneumonia (Ma, 2012) |
| 185 | *Rubia oncotricha* Hand.-Mazz. | Zuo | Rubiaceae | Herb | Whole plant | Pneumonia (Jia, 2005) |
| 186 | *Galium boreale* L. | Si-la-ga-bao | Rubiaceae | Herb | Whole plant | Pneumonia (Jia and Zhang, 2016) |
| 187 | *Galium boreale* var. *Ciliatum* Nalai | Sang-zi-ga-bo | Rubiaceae | Herb | Whole plant; root | Pneumonia, pneumorrhagia (Jia and Zhang, 2016) |
| 188 | Galium aparine Linn. Var. Tenerum (Gren. Et Godr.)Rchb. | Sang-zi-ga-bu | Rubiaceae | Herb | Whole plant; root | Pneumorrhagia (Wei et al., 2020) |
| 189 | *Rubia tibetica*Hook. f. | Zuo | Rubiaceae | Herb | Dried toot and rhizome | Lung heat (Control, 1996) |
| 190 | *Rubia cordifolia*L. | Zuo | Rubiaceae | Herb | Dried toot and rhizome | Lung heat (Ma, 2012) |
| 191 | *Galium verum* L. | Zuo | Rubiaceae | Herb | Whole plant | Pneumonia (Ma, 2012) |
| 192 | *Galium hoffmeisteri*(Klotzsch) Ehrendorfer et Schonbeck-Temesy ex R. R. Mill | Jia-ju-ma | Rubiaceae | Herb | Whole plant | Pneumonia (Jia and Zhang, 2016) |
| 193 | *Galium baldensiforme* Hand. -Mazz. | Sang-zi-ga-bo | Rubiaceae | Herb | Whole plant; root | Pneumonia, Pneumorrhagia (Jia and Zhang, 2016) |
| 194 | *Foeniculum vulgare* Mill. | Si-la-ga-bao | Umbelliferae | Herb | Fwhole plant | Lung heat, pneumonia, pulmonary tuberculosis (Luo, 1997) |
| 195 | *Cuminum cyminum* L. | Si-re-ga-bu | Umbelliferae |  | Dried fruit | Lung heat, pneumonia (Jia, 2005) |
| 196 | *Anthriscus sylvestris* (L.)  Hoffm. | Jia-wa | Umbelliferae | herb | Root | Cough due to dificiencyc of the lung (Jia and Zhang, 2016) |
| 197 | Bupleurum chinense DC. | Si-la-se-bao | Umbelliferae | herb | Root | Lung heat (Jia and Zhang, 2016) |
| 198 | *Pegaeophylon scapiflorum* (Hook. f. et Thoms.) Marq. et Shaw | Suo-luo-ga-bao | Cruciferae | Herb | Dried root and rhizome | Lung heat; hemoptysis in pulmonary disease (Luo, 1997) |
| 199 | *Erysimum hieraci*fpolium L. | Gang-tuo-ba | Cruciferae | Herb | Seed | Lung disease (Jia, 2005) |
| 200 | *Thlaspi arvense* L. | Tai-ka | Cruciferae | Herb | Seed | Cough with lung heat (Jia, 2005) |
| 201 | Capsella bursa - pastoris  (L.) Medic. | Suo-ga-wa | Cruciferae | herb | Whole palnt | Cough with lung heat(Herbalism, 2002) |
| 202 | *Erysimum amurense* Kitagawa. | Gang-tuo-ba | Cruciferae | Herb | Whole plant; fruit | Pulmonary tuberculosis (Jia and Zhang, 2016) |
| 203 | *Erysimum benthamii* P.Monnet | Gang-tuo-ba | Cruciferae | Herb | Seed | Lung disease (Jia and Zhang, 2016) |
| 204 | *Erysimum wardii* Polatschek | Gang-tuo-ba | Cruciferae | Herb | Seed | Cough from tuberculosis (Jia and Zhang, 2016) |
| 205 | *Erysimum funiculosum* J. D. Hooker & Thomson | Gang-tuo-ba | Cruciferae | Herb | Seed | Cough from tuberculosis (Jia and Zhang, 2016) |
| 206 | *Erysimum canescens*Roth | Gang-tuo-ba | Cruciferae | Herb | Seed | Cough from tuberculosis (Jia and Zhang, 2016) |
| 207 | *Megacarpaea delavayi* Franchet | Qia-gao-bei | Cruciferae | Herb | Whole plant | Cough with lung heat (Jia and Zhang, 2016) |
| 208 | *Porippa elata* (Hook. f. et Thoms.)  Hand. -Mazz | Gang-tuo-ba | Cruciferae | Herb | Seed; aboveground plant | Lung disease (Jia, 2005) |
| 209 | *Solms -laubachia* minor Hand.-Mazz. | Suo-luo-mu-bao | Cruciferae | Herb | Whole plant | Pneumonia, cough with lung heat (Jia and Zhang, 2016) |
| 210 | *Solms -Laubachia platycarpa* (Hook. f. et Thoms.) Botsch. | Suo-luo-mu-bao | Cruciferae | Herb | Whole plant | Pneumonia, cough with lung heat (Jia and Zhang, 2016) |
| 211 | *Solms -Laubachia pulcherrima* Muschl. | Suo-luo-mu-bao | Cruciferae | Herb | Whole plant[2、4、6、14、24] | Pneumonia, lung abscess, lung heat, tracheitis (Jia and Zhang, 2016) |
| 212 | *Sisymbrium altissimum* L. | Gang-tuo-ba | Cruciferae | Herb | Seed | Lung heat (Jia and Zhang, 2016) |
| 213 | *Sisymbrium brassiciforme* C. A. Mey. | Gang-tuo-ba | Cruciferae | Herb | Seed | Lung heat (Jia, 2005) |
| 214 | *Sisymbrium heteromallum* C. A. Mey. | Gang-tuo-ba | Cruciferae | Herb | Seed；fruit | Lung heat (Jia, 2005) |
| 215 | *Solms -Laubachia lanata* Botsch | Suo-luo-mu-bao | Cruciferae | Herb | Whole plant | Lung heat;pneumonia, lung heat, hemoptysis in pulmonary disease |
| 216 | *Solms -Laubachia linearifolia* (W. W. Smith) O. E. Sohulz. | Suo-luo-mu-bao | Cruciferae | Herb | Whole plant | Pneumonia, cough with lung heat (Jia and Zhang, 2016) |
| 217 | *Solms -Laubachia eurycarpa* (Maxim.) Botsch. | Suo-luo-mu-bao | Cruciferae | Herb | Whole plant | Pneumonia; lung abscess; lung heat; tracheitis (Luo, 1997) |
| 218 | *Thlaspi yunnanense*var. *dentata*Diels | Sa-ga-wa | Cruciferae | Herb | Whole plant | Pneumonia, lung heat (Jia and Zhang, 2016) |
| 219 | Cheiranthus younghusbandii Prain. | Su-luo-su-zha | Cruciferae | Herb | Whole plant | Pneumonia, lung abscess (Jia, 2005) |
| 220 | *Eremogone kansuensis*(Maxim.) Dillenb et Kadereit | A-zhong | Caryophyllaceae | Herb | Dried whole plant | Pneumonia (Wang, 2004) |
| 221 | *Arenaria kansuensis* Maxim. Var.ovatipetata Tsui. | A-zhong | Caryophyllaceae | Herb | Dried whole plant | Pneumonia (Wang, 2004) |
| 222 | *Arenaria przewalskii* Maxim. | Xiang-lin-mu-bu | Caryophyllaceae | Herb | Dried whole plant | Pneumonia, pulmonary tuberculosis, pulmonary cough and wheezing (Ma, 2012) |
| 223 | *Eremogone bryophylla* (Fernald) Pusalkar et D.K.Singh | A-zhong-ga-bao | Caryophyllaceae | Herb | Whole plant | Cough with lung heat; bronchitis (Wei et al., 2020) |
| 224 | *Dolophragma juniperinum* (D. Don) Fenzl； | A-zhong-ga-bao | Caryophyllaceae | Herb | Whole plant | Cough with lung heat; bronchitis (Jia and Zhang, 2016) |
| 225 | *Arenaria lancangensis*L. H. Zhou. | A-zhong-ga-bao | Caryophyllaceae | Herb | Whole plant | Cough with lung heat, bronchitis (Luo, 1997) |
| 226 | *Eremogone festucoides* (Benth.) Pusalkar & D.K.Singh | A-zhong-ga-bao | Caryophyllaceae | Herb | Whole plant | Lung heat; pneumonia,(Herbalism, 2002) |
| 227 | Arenaria kansuensis Maxim. | A-zhong-ga-bao | Caryophyllaceae | Herb | Root | Pneumonia (Wei et al., 2020) |
| 228 | *Arenaria serpyllifolia* Linn. | Du-zai-gang-xia | Caryophyllaceae | Herb | Whole plant | Pulmonary tuberculosis; pneumonia; cough with lung heat; cough of pulmonary furuncles (Luo, 1997) |
| 229 | *Lepyrodiclis holosteoides* (C. A. Meyer) Fenzl. ex Fisher et C. A. Meyer | Xing-zhi-ga-bu | Caryophyllaceae | Herb | Whole plant | Cough with lung heat (Jia, 2005) |
| 230 | *Silene conoidea* L. | Pu-po-zi | Caryophyllaceae | Herb | Whole plant | Pulmonary tuberculosis (Luo, 1997) |
| 231 | *Silene jenisseensis* Willd. | Pu-po-zi | Caryophyllaceae | Herb | Whole plant | Pulmonary tuberculosis (Luo, 1997) |
| 232 | *Silene repens* Patr. | Pu-po-zi | Caryophyllaceae | Herb | Whole plant | Pulmonary tuberculosis (Luo, 1997) |
| 233 | *Stellaria graminea* L. | Qi-xiang-ga-mao | Caryophyllaceae | Herb | Whole plant | Pneumonia (Jia and Zhang, 2016) |
| 234 | *Arisaema elephas* Buchet | Ta-wa | Araceae | Herb | Tuber | Chronic bronchitis, bronchiectasis (Jia and Zhang, 2016) |
| 235 | *Arisaema erubescens* (Wall.) Schott | Ta-wa | Araceae | Herb | Tuber | Chronic bronchitis, bronchiectasis (Jia and Zhang, 2016) |
| 236 | *Arisaema flavum* (Forsk.)  Schott； | Da-wei-zha-wa | Araceae | Herb | Tuber | Chronic bronchitis; bronchiectasis (Jia and Zhang, 2016) |
| 237 | *Arisaema franchetianum* Engl. | Da-wei-zha-wa | Araceae | Herb | Tuber | Chronic bronchitis; bronchiectasis (Jia and Zhang, 2016) |
| 238 | *Aristolochia gentilis* Franch. | Ba-le-ga | Aristolochiaceae | Herb | Stem; rhizome | Lung heat (Jia and Zhang, 2016) |
| 239 | *Aristolochia griffithii*Hook. f. et Thoms. ex Duchartre | Pa-le-ga | Aristolochiaceae | Herb | Stem; aboveground parts | Lung heat (Yang, 1987) |
| 240 | *Aristolochia heterophylla* Hemsl. | Pa-le-ga | Aristolochiaceae | Herb | Stem; root; fruit | Lung heat (Jia and Zhang, 2016) |
| 241 | *Aristolochia moupinensis* Franch. | Pa-le-ga | Aristolochiaceae | Herb | Root; rhizome; aboveground parts | Lung heat (Jia, 2005) |
| 242 | *Aristolochia debilis* Sieb. et Zucc. | Ba-li-ga | Aristolochiaceae | Herb | Root; rhizome | Lung heat (Control, 1996) |
| 243 | *Aristolochia contorta* Bunge. |  | Aristolochiaceae | Herb | Dried aboveground parts | Lung heat (Zhong and Song, 2020) |
| 244 | *Aristolochia macrocarpa C. Y. Wu et S. Y. Wu* | Pa-le-ga | Aristolochiaceae | Herb | Dried aboveground parts | Lung heat (Editorial Committee of Tibetan Medicine Journal, 1991) |
| 245 | *Artemisia adamsii*Bess. | Kan-ba-se-bao | Comopsite | Herb | Aboveground parts | Pneumonia (Jia and Zhang, 2016) |
| 246 | *Anaphalis nepalensis* (Spreng.)Hand. -Mazz. | Gan-da-ba-zha | Comopsite | Herb | Aboveground parts | Tracheitis (Jia and Zhang, 2016) |
| 247 | *Ajania tenuifolia*(Jacq.) Tzvel. | Kan-ba-ga-bao | Comopsite | Herb | Baranches and stem | Lung disease (Jia and Zhang, 2016) |
| 248 | *Aucklandia lappa* Decne. | Ru-da | Comopsite | Herb | Dried root | Pneumonia (Yang, 1987) |
| 249 | *Artemisia frigida* Willd. | Kan-jia | Comopsite | Herb | Dried aboveground parts | Lung disease (Control, 1996) |
| 250 | *Aster souliei*Franch. | Mei-duo-lou-mei | Comopsite | Herb | Dried inflorescence | Bronchitis (Zhong and Song, 2020) |
| 251 | *Aster flaccidus* Bunge | Mei-duo-lou-mei | Comopsite | Herb | Dried inflorescence | Bronchitis (Zhong and Song, 2020) |
| 252 | *Aster diplostephioides* (DC.) C. B. Clarke | Mei-duo-lou-mei | Comopsite | Herb | Dried inflorescence | Bronchitis (Zhong and Song, 2020) |
| 253 | *Artemisia dubia*Wall. ex Bess. | Pu-mang | Comopsite | Herb | Dried aboveground parts | Cough with lung heat;; tracheitis (Zhong and Song, 2020) |
| 254 | *Artemisia scoparia* Waldst. et Kit. | Ca-er-bang | Comopsite | Herb | Whole plant | Lung heat(Control, 1996) |
| 255 | Artemisia adamsii Besser | Kan-ba-se-bao | Comopsite | Herb | Dried aboveground parts | Pneumonia (Yang, 1987) |
| 256 | *Artemisia vestita* Wall.ex Bess. | Pu-er-na | Comopsite | Herb | Dried aboveground parts | Cough with lung heat, tracheitis (Zhong and Song, 2020) |
| 257 | *Artemisia capillaris* Thunb. | Cha-weng | Comopsite | herb | Seedling; root | Tracheitis, (Jia, 2005) |
| 258 | *Artemisia carvifolia* Buch.-Ham. ex Roxb. | Kan-ba | Comopsite | Herb | Whole plant | Lung disease (Jia and Zhang, 2016) |
| 259 | *Artemisia conaensis* Ling et Y. R. Ling | Cha-er-bang | Comopsite | Herb | Whole plant | Tracheitis, (Jia and Zhang, 2016) |
| 260 | *Artemisia demissa* Krasch | Cha-er-wang-mu-bao | Comopsite | Herb | Whole plant | Lung disease (Jia, 2005) |
| 261 | *Artemisia desertorum* Spreng | Yao-mao-na-bao | Comopsite | Herb | Whole plant | Tracheitis (Jia and Zhang, 2016) |
| 262 | *Artemisia dracunculus* L. | - | Comopsite | Herb | Whole plant | Lung disease (Jia and Zhang, 2016) |
| 263 | *Artemisia dubia* var.*subdigitata* (Mattf.)  Y. R. Ling | Pu-mang-na-bu | Comopsite | Herb | Aboveground parts | Cough with lung heat; tracheitis; emphysema (Jia and Zhang, 2016) |
| 264 | *Artemisia stricta*Heyne. et DC. nec Edgew.: Fisc ex Herd. | Cha-er-wang-ga-bao | Comopsite | Herb | Whole plant | Tracheitis (Jia, 2005) |
| 265 | *Artemisia eriopoda* Bge. | Cha-er-wang-na-bao | Comopsite | Herb | Whole plant | Lung disease (Control, 1996) |
| 266 | *Artemisia giraldii* Pamp. | Cha-er-jiang | Comopsite | Herb | Root; seedling | Tracheitis (Jia and Zhang, 2016) |
| 267 | *Artemisia halodendron* Turez. ex Besser | Kan-a-zhong | Comopsite | Herb | Aboveground parts | Pneumonia (Jia and Zhang, 2016) |
| 268 | *Artemisia integrifolia* L. | Pu-mang-na-bo | Comopsite | Herb | Aboveground parts | Cough with lung heat; emphysema; tracheitis (Jia and Zhang, 2016) |
| 269 | *Artemisia japonica* Thunb. | Cha-er-wang-na-bao | Comopsite | Herb | Aboveground parts | Lung disease (Jia, 2005) |
| 270 | *Artemisia minor* Jacp. ex Bess. | Kan-ba-a-zhong | Comopsite | Herb | Leaf; new shoot | Pneumonia(Herbalism, 2002) |
| 271 | *Artemisia moorcroftiana* Wall. ex DC. | Kan-ba-ma-bao | Comopsite | Herb | Aboveground parts | Lung disease (Jia and Zhang, 2016) |
| 272 | *Artemisia ordosica* Krasch. | Cha-er-wang-na-bao | Comopsite | Herb | Root; seedling | Cough with lung heat; emphysema; tracheitis (Jia and Zhang, 2016) |
| 273 | *Artemisia pewzowi* C. Winkl. | Cha-er-wang-na-bao | Comopsite | Herb | Whole plant | Lung disease (Control, 1996) |
| 274 | *Artemisia sieversiana* Ehrh. ex Willd. | Kan-ba-ga-bu | Comopsite | Herb | Aboveground parts | Lung disease (Jia, 2005) |
| 275 | *Aster asteroides* (DC.) O. Ktze. | Mei-duo-lou-mei | Comopsite | Herb | Inflorescence | Bronchitis (Jia and Zhang, 2016) |
| 276 | *Aster handelii* Onno | Lu-mei | Comopsite | Herb | Inflorescence | Bronchitis (Jia and Zhang, 2016) |
| 277 | *Aster himalaicus* C. B. Clarke | Qu-de-wa | Comopsite | Herb | Inflorescence | Cough with lung heat (Jia and Zhang, 2016) |
| 278 | *Aster souliei* Franch. | Lu-mei | Comopsite | Herb | Inflorescence; root | Bronchitis(Herbalism, 2002) |
| 279 | *Aster tongolensis* Franch. | Mei-duo-lou-mo | Comopsite | Herb | Inflorescence | Bronchitis (Jia and Zhang, 2016) |
| 280 | *Calendula officinalis* L. | Ge-gong-mai-duo | Comopsite | Herb | Inflorescence | Lung disease (Jia and Zhang, 2016) |
| 281 | *Carthamus tinctorius* L. | Ku-gong | Comopsite | Herb | Flower | Pneumonia, pulmonary tuberculosis (Jia, 2005) |
| 282 | *Cirsium exculentum* (Sievers) C. A. Mey. | Qia-rao-niu-ma | Comopsite | Herb | Whole plant | Lung heat, lung abscess(Herbalism, 2002) |
| 283 | *Pseudognaphalium hypoleucum* (Candolle) Hilliard & B. L. Burtt | Ga-na-ba | Comopsite | Herb | Whole plant | Cough with lung heat (Jia and Zhang, 2016) |
| 284 | *Ixeris chinensis* (Thunb.) Nakai | Za-chi | Comopsite | Herb | Whole plant | Cough with lung heat, pulmonary tuberculosis, lung abscess (Jia and Zhang, 2016) |
| 285 | *Leibnitzia anandria*(Linnaeus) Turczaninow | Zhao-he-qiong-wa | Comopsite | Herb | Whole plant | Cough with lung heat (Jia and Zhang, 2016) |
| 286 | *Leibnitzia nepalensis* (Kunze) Kitamura | Gun-ba-jia-jia | Comopsite | Herb | Whole plant | Cough with lung heat (Jia and Zhang, 2016) |
| 287 | *Pertya discolor* Rehd. | Qi-xiang | Comopsite | Herb | Flower | Tracheitis, pulmonary tuberculosis (Jia, 2005) |
| 288 | *Pertya discolor* var. *calvescens* Ling | Qi-xiang | Comopsite | Herb | Flower | Tracheitis, pulmonary tuberculosis (Jia and Zhang, 2016) |
| 289 | *Pertya monocephala* W. W. Smith | Qi-xiang | Comopsite | Herb | Flower | Tracheitis, pulmonary tuberculosis (Jia and Zhang, 2016) |
| 290 | *Pertya phylicoides* J. F. Jeffrey | Qia-ma | Comopsite | Herb | Flower | Tracheitis, pulmonary tuberculosis; bronchitis (Jia and Zhang, 2016) |
| 291 | Senecio scandens Buch.-Ham. ex D. Don | Sai-bao-gu-zhui | Comopsite | Herb | Whole plant | Pneumonia (Jia and Zhang, 2016) |
| 292 | *Tagetes erecta* L. | Xiao-wan-shou-ju | Comopsite | Herb | Whole plant | Pulmonary abscess (Jia and Zhang, 2016) |
| 293 | *Taraxacum mongolicum* Hand.-Mazz. | Ke-er-mang | Comopsite | Herb | Whole plant | Pneumonia (Jia and Zhang, 2016) |
| 294 | *Dolomiaea souliei*var. *cinerea* (Y. Ling) Q. Yuan | Bu-ga-mo-la | Comopsite | Shrub | Root | Lung disease (Jia and Zhang, 2016) |
| 295 | *Artemisia mongolica* (Fisch. ex Bess.) Nakai | Pu-er-mang-mo-bao | Comopsite | Herb | Aboveground parts | Lung disease (Ma, 2012) |
| 296 | *Asparagus cochinchinensis* (Lour.) Merr. | Ni-xin | Liliaceae | Herb | Tuber | Atrophic, abscess of lung (Jia and Zhang, 2016) |
| 297 | *Asparagus filicinus*D. Don | Nie-xiang | Liliaceae | Herb | Tuber | Persistent pulmonary cough (Jia and Zhang, 2016) |
| 298 | *Polygonatum odoratum*(Mill.)Druce | Lu-ni | Liliaceae | Herb | Dried rhizome | Pulmonary dryness (Zhong and Song, 2020) |
| 299 | *Fritillaria cirrhosa* D. Don | A-bu-ka | Liliaceae | Herb | Dried bulb | Tracheitis, cough with lung heat (Wei et al., 2020) |
| 300 | *Fritillaria unibracteata* Hsiao et K. C. Hsia | A-bu-ka | Liliaceae | Herb | Dried bulb | Tracheitis, cough with lung heat(Control, 1996) |
| 301 | *Fritillaria przewalskii* Maxim. | A-bu-ka | Liliaceae | Herb | Dried bulb | Tracheitis, cough with lung heat(Control, 1996) |
| 302 | *Fritillaria delavayi* Franch | A-bu-ka | Liliaceae | Herb | Dried bulb | Tracheitis, cough with lung heat(Control, 1996) |
| 303 | Fritillaria cirrhosa D. Don | A-bei-ka | Liliaceae | Herb | Dried bulb | Lung heat(Herbalism, 2002) |
| 304 | *Fritillaria taipaiensis* P. Y. Li | A-bu-ka | Liliaceae | Herb | Dried bulb | Tracheitis; cough with lung heat (Zhong and Song, 2020) |
| 305 | *Cardiocrinum giganteum*  (Wall.) Makino | Da-si-mei-duo-xian-ba | Liliaceae | Herb | Dried bulb | Tracheitis, cough with lung heat (Jia, 2005) |
| 306 | *Lilium davidii* Duchartre ex Elwes | A-bei-ka | Liliaceae | Herb | Dried bulb | Cough with lung heat (Control, 1996) |
| 307 | *Fritillaria sichuanica* S. C. Chen | A-pi-ka | Liliaceae | Herb | Dried bulb | Cough with lung heat (Jia and Zhang, 2016) |
| 308 | *Lilium bakerianum* Coll.et Hemsl | Da-ri-mai-duo | Liliaceae | Herb | Dried bulb | Lung disease (Jia and Zhang, 2016) |
| 309 | *Lilium duchartrei* Franch. | Da-ri-mai-duo | Liliaceae | Herb | Dried bulb | Lung disease (Jia and Zhang, 2016) |
| 310 | *Lilium lophophorum*  (Bur. et Franch.) Franch. | Da-si-mai-duo | Liliaceae | Herb | Dried bulb | Cough in Lung disease (Jia and Zhang, 2016) |
| 311 | *Lilium pumilum* DC. | A-bi-ka | Liliaceae | Herb | Dried bulb | Lung disease (Jia and Zhang, 2016) |
| 312 | *Lilium wardii* Stapf exStearn | Da-se-duo-mei | Liliaceae | Herb | Dried bulb | Cough in Lung disease (Jia, 2005) |
| 313 | *Notholirion bulbuliferum* (Lingelsh. ex H. Limpricht)Stearn | Da-si-mai-duo | Liliaceae | Herb | Dried bulb | Cough in Lung disease (Jia and Zhang, 2016) |
| 314 | *Ophiopogon intermedius* D. Don | Zha-zhu | Liliaceae | Herb | Root | Tracheitis (Jia and Zhang, 2016) |
| 315 | *Polygonatum prattii* Baker | Luo-ni | Liliaceae | Herb | Tuber | Xeropulmonary cough (Jia and Zhang, 2016) |
| 316 | *Polygonatum sibiricum* Delar. ex Redoute | Re-ni | Liliaceae | Herb | Tuber | Pulmonary tuberculosis (Herbalism, 2002) |
| 317 | *Smilax menispermoidea* A. DC. | Zhe-er-niu | Liliaceae | Shrub | Root | Cough from tuberculous tracheitis (Jia and Zhang, 2016) |
| 318 | *Berbers brachypoda* Maxim. | Ji-er-wa | Liliaceae | Herb | Root; stem | Pneumonia (Jia and Zhang, 2016) |
| 319 | *Berberis ferdinandi-coburgii* Schneid. | Jie-wei-wa-xing | Liliaceae | Herb | Root | Pneumonia (Jia and Zhang, 2016) |
| 320 | *Bergenia pacumbis* C. Y. Wuet J. T. Pan | Ka-tu-er | Saxifragaceae | Herb | Rhizome | Pneumonia, Pulmonary tuberculosis (Jia and Zhang, 2016) |
| 321 | *Saxifraga atrata* Engl. | Song-di-ga-bao | Saxifragaceae | Herb | Whole plant | Pneumonia, (Luo, 1997) |
| 322 | *Saxifraga brunonis* Wall. ex Ser. | Song-di | Saxifragaceae | Herb | Whole plant | Pulmonary tuberculosis; pulmonary crests (Herbalism, 2002) |
| 323 | *Saxifraga divaricata* Engl. et Irmsch. | Song-di-ga-bao | Saxifragaceae | Herb | Whole plant | Lung disease (Luo, 1997) |
| 324 | Saxifraga gemmuligera Engl. | Xiang-lian-mu-bao | Saxifragaceae | Herb | Dried-whole plant | Pulmonary tuberculosis; pneumonia (Ma, 2012) |
| 325 | (*Bergenia purpurascens* (Hook. F. et Thoms.)Engl.) | Li-ga-du | Saxifragaceae | Herb | Dried-whole plant | Lung heat (Luo, 1997) |
| 326 | *Bergenia crassifolia*(L.)Fritsch. | Li-ga-du | Saxifragaceae | Herb | Dried-whole plant | Lung heat (Zhong and Song, 2020) |
| 327 | *Betula delavayi* Franch. | Chuo-ba | Betulaceae | Tree | Endothelium of stem | Pneumonia (Luo, 1997) |
| 328 | *Betula platyphylla* Suk. | Chuo-ba | Betulaceae | Tree | Endothelium of stem | Pneumonia (Jia, 2005) |
| 329 | *Betula utilis* D. Don | Qi-bi-ba | Betulaceae | Tree | Endothelium of stem | Pneumonia (Jia, 2005) |
| 330 | *Japanobotrychum lanuginosum* (Wallich ex Hooker et Greville) M. Nishida ex Tagawa | Jia-yi-a-ma | botrychiaceae | Herb | Whole plant | Pulmonary cough and wheezing (Jia and Zhang, 2016) |
| 331 | *Botrychium lunaria* Sw. | Jia-yi-a-ma | botrychiaceae | Herb | Whole plant | Pulmonary cough and wheezing (Jia and Zhang, 2016) |
| 332 | *Lycium barbarum* L. | Pang-ma | Solanaceae | Tree | Velamen | Pulmonary tuberculosis (Jia and Zhang, 2016) |
| 333 | *Mandragora caulescens* C. B. Clarke. | Tang-chong-ga-bo-man-ba | Solanaceae | Tree | Root; rhizome; fruit | Lung abscess (Luo, 1997) |
| 334 | *Buddleja crispa* Benth. |  | Loganiacea | Herb | Whole plant | Lung heat (Jia and Zhang, 2016) |
| 335 | *Caryopteris forrestii* Diels | Pu-na | Verbenaceae | Herb | Whole plant | Tracheitis (Jia and Zhang, 2016) |
| 336 | *Vitex trifolia* L. |  | Verbenaceae | Shrub | Fruit | Pulmonary crests; Pneumonia (Jia and Zhang, 2016) |
| 337 | *Catabrosa aquatica*  (L.)  Beauv. | Dan-bu-ga-la | Gramineae | Herb | Whole plant | Pneumonia, pulmonary trauma (Jia, 2005) |
| 338 | *Hordeum vulgare* var. *coeleste* Linnaeus | Nai | Gramineae | Herb | Fruit | Cough with lung heat; infantile pneumonia (Wei et al., 2020) |
| 339 | *Indosasa crassiflora* McClure | Lv-ju-feng | Gramineae | Herb | A dry mass of fluid in a rod | Lung heat, (Jia and Zhang, 2016) |
| 340 | (*Bambusa textilis* McClure)、、 | Ju-gang | Gramineae | Shrub | A dry mass of fluid in a rod | Lung heat (Zhong and Song, 2020) |
| 341 | *Krascheninnikovia ceratoides* (Linnaeus) Gueldenstaedt | Qi-xiang | Chenopodiaceae | Herb | Fruit | Lung disease (Ma, 2012) |
| 342 | *Kali collinum (Pall.)* Akhani et Roalson | Da-cai-er | Chenopodiaceae | Herb | Aboveground parts | Lung disease (Jia, 2005) |
| 343 | *Ceratostigma griffithii* C. B. Clarke |  | Plumbaginaceae | Herb | Whole plant | Lung disease (Jia and Zhang, 2016) |
| 344 | *Ceratostigma minus* Stapf ex Prain | Qia-pao-zi | Plumbaginaceae | Herb | Root | Hemoptysis in pulmonary disease (Administration, 2020) |
| 345 | *Cinnamomum inters* Reinw. ex Bl. | Xing-ca | Lauraceae | Tree | Bark; branches | Lung abscess (Jia and Zhang, 2016) |
| 346 | *Cinnamomum tamala* (Buch.-Ham.) Th | Xing-ca | Lauraceae | Tree | Branches; bark | Lung abscess (Luo, 1997) |
| 347 | *Cinnamomum wilsonii* Gamble | Xiang-cha | Lauraceae | Tree | Branches; bark | Lung abscess (Ma, 2012) |
| 348 | *Cinnamomum cassia* Presl | Xin-ca | Lauraceae | Tree | Bark | Abscess of lung (Ma, 2012) |
| 349 | *Corydalis crispa* Prain | Long-e | Papaveraceae | Herb | Whole palnt | Pneumonia (Jia and Zhang, 2016) |
| 350 | *Corydalis hookeri* Prain | Dang-ri-si-wa | Papaveraceae | Herb | Whole palnt | Pneumococcosis (Ma, 2012) |
| 351 | *Corydalis pachypoda* Hand.- Mazz. | Rui-jin-ba | Papaveraceae | Herb | Whole palnt | Lung disease (Jia and Zhang, 2016) |
| 352 | *Corydalis stenantha* Franch. | Mu-qiong-s-wa | Papaveraceae | Herb | Whole palnt | Cough in Lung disease pulmonary furnucles (Jia and Zhang, 2016) |
| 353 | *Corydalis straminea* Maxim. | Yu-zhou-si-wa | Papaveraceae | Herb | Whole palnt | Cough from pneumonia (Jia and Zhang, 2016) |
| 354 | *Corydalis tibetoalpina* C. Y. Wu et T. Y. Su | Mu-qiong-si-wa | Papaveraceae | herb | Whole palnt | Cough in Lung disease pulmonary furnucles (Jia, 2005) |
| 355 | *Meconopsis argemonantha* Prain | O-bei-ga-bao | Papaveraceae | herb | Flower | Lung heat (Jia, 2005) |
| 356 | *Meconopsis discigera* Prain | Mu-qiong-dian-yun | Papaveraceae | herb | Whole palnt | Pneumonia, cough with lung heat (Jia and Zhang, 2016) |
| 357 | *Meconopsis florindae* Kingdon - Ward | O-bei-sai-bao | Papaveraceae | herb | Whole palnt | Lung heat (Jia and Zhang, 2016) |
| 358 | *Meconopsis grandis* Prain | O-bei-mo-bao | Papaveraceae | herb | Whole palnt | Pneumonia, Lung heat (Jia and Zhang, 2016) |
| 359 | *Meconopsis henrici* Bur.et Franch. | O-bei-wan-bao | Papaveraceae | herb | Whole palnt | Pneumonia, Lung heat (Jia and Zhang, 2016) |
| 360 | *Meconopsis horridula* Hook. f. et Thoms. | A-qia-cai-wen | Papaveraceae | Herb | Flower | Lung heat (Jia and Zhang, 2016) |
| 361 | *Meconopsis impedita* Prain | O-bei-ma-bo | Papaveraceae | Herb | Whole palnt | Lung heat (Jia, 2005) |
| 362 | *Meconopsis napanlensis* DC. Prodr. | O-bei-sai-bao | Papaveraceae | Herb | Whole palnt | Lung heat (Jia and Zhang, 2016) |
| 363 | *Meconopsis paniculata* (D. Don.)  Prain | O-bei-sai-bo | Papaveraceae | Herb | Whole palnt | Lung heat, pneumonia (Jia, 2005) |
| 364 | *Meconopsis pinnatifolia* C. Y. Wu et H. Chuang ex L. H. Zhou | O-bei-ma-bao | Papaveraceae | Herb | Whole palnt | Lung heat (Jia and Zhang, 2016) |
| 365 | *Meconopsis simplicifolia* (D. Don) Walp. Rep. Bot. Syst. | Mu-qiong-dian-yun | Papaveraceae | Herb | Whole palnt | Lung heat (Luo, 1997) |
| 366 | *Meconopsis speciosa* Prain | O-bei-wan-bo | Papaveraceae | Herb | Flower | Lung heat (Jia, 2005) |
| 367 | *Meconopsis superba* King ex Prain | O-bei-ga-bao | Papaveraceae | Herb | Flower | Lung heat (Jia, 2005) |
| 368 | *Meconopsis torrquata* Prain | Wu-bai-en-bu | Papaveraceae | Herb | Whole palnt | Lung heat, pneumonia (Jia, 2005) |
| 369 | *Hypecoum leptocarpum*Hook. f. et Thoms.， | Ba-er-ba-da | Papaveraceae | Herb | Whole palnt | Cough from pneumonia (Qianyutuo, 2016) |
| 370 | *Hypecoum erectum*L. | Ba-er-ba-da | Papaveraceae | Herb | Whole palnt | Cough from pneumonia (Jia, 2005) |
| 371 | *Meconopsis integrifolia* (Maxim.) Franch. | Wu-bai-en-bu | Papaveraceae | Herb | Whole palnt | Pneumonia, lung heat (Luo, 1997) |
| 372 | *Meconopisis quintuplinervia* Regel | Wu-bai-en-bu | Papaveraceae | Herb | Whole palnt | Pneumonia; lung heat(Control, 1996) |
| 373 | *Meconopsis lancifolia*(Franch.) Franch. ex Prain | Wu-bai-en-bu | Papaveraceae | Herb | Whole palnt | Pneumonia, lung heat (Jia, 2005) |
| 374 | *Meconopsis punicea* Maxim. | Wu-bai-en-bu | Papaveraceae | Herb | Whole palnt | Pneumonia, lung heat (Luo, 1997) |
| 375 | *Cuscuta chinensis* Lam. | Zhu-xia-ba | Convolvulaceae | Herb | Dried aboveground parts | Pneumonia; lung heat (Ma, 2012) |
| 376 | *Cuscuta australis* R. Br. | Zhu-xia-ba | Convolvulaceae | Herb | Dried aboveground parts | Pneumonia; lung heat (Zhong and Song, 2020) |
| 377 | *Convolvulus ammannii* Desr. | Bo-er-qiong | Convolvulaceae | Herb | Whole plant | Lung disease (Jia and Zhang, 2016) |
| 378 | *Cuscuta approximata*Babington*.* | Zhu-xia-ba | Convolvulaceae | Herb | Whole plant | Lung heat (Jia and Zhang, 2016) |
| 379 | *Cuscuta europaeam* L. | Sai-ge | Convolvulaceae | Herb | Whole plant | Pneumonia; lung heat (Jia and Zhang, 2016) |
| 380 | *Cuscuta japonica* Choisy | Sai-ge | Convolvulaceae | Herb | Whole plant | Pneumonia; lung heat (Jia and Zhang, 2016) |
| 381 | *Cuscuta reflexa* Roxb. | Zhu-xiao | Sai-ge | Convolvulaceae | Herb | Whole plant (Jia and Zhang, 2016) |
| 382 | *Elaeagnus pungens* Thunb. | Da-bu | Elaeagnaceae | Shrub | Fruit | Lung disease (Jia and Zhang, 2016) |
| 383 | *Hippophae neurocarpa* S. W. Liu et T. N. He | Da-bu | Elaeagnaceae | Shrub | Fruit | pulmonary tuberculosis (Ma, 2012) |
| 384 | *Hippophae rhamnoides* subsp. *Sinensis* Rousi | Da-bu | Elaeagnaceae | Shrub | Fruit | Lung neoplasms (Jia and Zhang, 2016) |
| 385 | *Hippophae rhamnoides* subsp. *yunnanensis* Rousi | Da-bu | Elaeagnaceae | Shrub | Fruit | Lung neoplasms (Jia and Zhang, 2016) |
| 386 | *Hippophae salicifolia* D. Don | Da-bu | Elaeagnaceae | Shrub | Fruit | Lung disease (Jia and Zhang, 2016) |
| 387 | *Hippurs vulgaris* L. | Dong-bu-ga-la | Elaeagnaceae | Herb | Whole plant | Pneumoonia, Cough in Lung disease pulmonary furuncles, pulmonary trauma; pulmonary tuberculosis; pulmonary fever (Jia and Zhang, 2016) |
| 388 | *Hippophae rhamnoides* L. | Da-bu | Elaeagnaceae | Shrub | Dried ripe fruit or decoction and paste of fruit | Tracheitis (Bairuozana, 2016) |
| 389 | *Hippophae rhamnoides*subsp.*wolongensis*Y. S. Lian et al. | Da-bu | Elaeagnaceae | Shrub | Dried ripe fruit or decoction and paste of fruit | Tracheitis (Zhong and Song, 2020) |
| 390 | *Hippophae gyantsensis* (Rousi) Lian | Da-bu | Elaeagnaceae | Shrub | Dried ripe fruit or decoction and paste of fruit | Tracheitis (Gema, 2016) |
| 391 | *Hippophae. thibetana* Schlechtend | Da-bu | Elaeagnaceae | Shrub | Dried ripe fruit or decoction and paste of fruit | Tracheitis (Administration, 2020) |
| 392 | *Euphrasia regelii* Wettst | Xing-tuo-li-ga-bao | Scrophulariaceae | Herb | Flower | Cough with lung heat (Jia and Zhang, 2016) |
| 393 | *Lagotis brachystachya* Maxim. | Zhi-da-sa-zeng | Scrophulariaceae | Herb | Whole plant | Cough with lung heat; lung abscess (Ma, 2012) |
| 394 | *Lagotis macrosiphon*Tsoong et Yang | Hong-lin | Scrophulariaceae | Herb | Whole plant | Lung disease (Jia and Zhang, 2016) |
| 395 | *Lagotis praecox* W. W. Smith | Hong-lian | Scrophulariaceae | Herb | Whole plant | Lung disease (Jia and Zhang, 2016) |
| 396 | *Lagotis ramalana* Batalin | Hong-lin | Scrophulariaceae | Herb | Whole plant | Lung disease (Jia and Zhang, 2016) |
| 397 | *Lagotis yunnanensis* W. W. Smith | Hong-lun | Scrophulariaceae | Herb | Whole plant | Lung disease (Jia and Zhang, 2016) |
| 398 | *Lancea tibetica* Hook. f. et Thomson | Ba-ya-ba | Scrophulariaceae | Herb | Whole plant | Pneumoonia; lung abscess (Jia and Zhang, 2016) |
| 399 | *Lagotis brevituba* Maxim. | Hong-lian | Scrophulariaceae | Herb | Dried whole plant | Lung disease (Jia, 2005) |
| 400 | *Lagotis integra* W. W. Smith. | Hong-lian | Scrophulariaceae | Herb | Dried whole plant | Lung disease (Zhong and Song, 2020) |
| 401 | *Lagotis glauca* Gaertn. | Hong-lian | Scrophulariaceae | Herb | Dried whole plant | Lung disease (Ma, 2012) |
| 402 | *Lagotis alutacea* W. W. Smith | Hong-lian | Scrophulariaceae | Herb | Dried whole plant | Lung disease (Zhong and Song, 2020) |
| 403 | *Picrorhiza scrophulariiflora* (Pennell) D. Y. Hong |  | Scrophulariaceae | Herb | Dried rhizome | Lung heat (Zhong and Song, 2020) |
| 404 | *Pedicularis cranolopha* Maxim. | Lang-nuo-sai-bao | Scrophulariaceae | Herb | Flower | Pneumoonia (Jia and Zhang, 2016) |
| 405 | *Pdicularis muscicola* Maxim. | Lu-ru-mu-bao | Scrophulariaceae | Herb | Flower | Lung disease (Jia and Zhang, 2016) |
| 406 | *Lancea tibetica* Hook. f et Thoms. | Ba-ya-ba | Scrophulariaceae | Herb | Dried whole plant | Lung abscess (Jia, 2005) |
| 407 | *Koenigia divaricata*(L.) T. M. Schust. & Reveal | Cha-fen-liao | Polygonaceae | Herb | Root | Pulmonary fever dullness (Yutuo, 2012) |
| 408 | *Fagopyrum tataricum* (L.)  Gaert. | Cha-wu | Polygonaceae | Herb | Whole plant | Lung cancer (Jia and Zhang, 2016) |
| 409 | *Fallopia aubertii* (L. Henry) Holub | Le-zhe | Polygonaceae | Herb | Stem | Lung disease (Ma, 2012) |
| 410 | *Polygonum paleaceum*  (Wall. ex Hook. f.) Yonekura et H. Ohashi | La-gang-yong-wa | Polygonaceae | Herb | Tuber | Cough with lung heat (Jia and Zhang, 2016) |
| 411 | *Polygonum sibiricum* (Laxmann) Tzvelev | Qu-ma-zi | Polygonaceae | Herb | Whole plant | Lung disease (Jia and Zhang, 2016) |
| 412 | *Polygonum tortuosum* (D. Don) T. M. Schust. & Reveal | Ni-luo | Polygonaceae | Herb | Whole plant | Pulmonary fever dullness (Jia, 2005) |
| 413 | *Polygonum viviparum* L. | Ran-bu | Polygonaceae | Herb | Whole plant | Lung disease (Jia and Zhang, 2016) |
| 414 | *Rumex acetosa* L. | Xiao-mang | Polygonaceae | Herb | Root; rhizome | Pulmonary tuberculosis (Jia, 2005) |
| 415 | *Rumex nepalensis* Spreng. | Xiao-mang | Polygonaceae | Herb | Root | Pulmonary tuberculosis;lung heat (Jia, 2005) |
| 416 | *Fagopyrum dibotrys* (D. Don) Hara | Cha-wu | Polygonaceae | Herb | Dried rhizome | Lung cancer (Jia, 2005) |
| 417 | *Fagopyrum esculentum*Moench | Cha-wu | Polygonaceae | Herb | Dried rhizome | Lung cancer (Luo, 1997) |
| 418 | *Geranium dahuricum* DC. | La-gang | Geraniaceae | Herb | Whole plant with fruit | Pneumonia (Jia and Zhang, 2016) |
| 419 | *Geranium platyanthum* Duthie. | La-gang | Geraniaceae | Herb | Aboveground parts | Pneumonia (Jia and Zhang, 2016) |
| 420 | *Geranium napuligerum* Franch. | La-gang | Geraniaceae | Herb | Whole plant | Pneumonia, lung heat (Jia and Zhang, 2016) |
| 421 | *Geranium pratense* L. | La-gang | Geraniaceae | Herb | Aboveground parts | Lung heat (Jia, 2005) |
| 422 | *Geranium pylzowianum* Maxim. | La-gang | Geraniaceae | Herb | Whole plant | Pneumonia, lung heat(Jia, 2005) |
| 423 | *Geranium refractum* Edgew. et Hook. f. | Bang-ma-man-ba | Geraniaceae | Herb | Root | Lung disease (Jia and Zhang, 2016) |
| 424 | *Geranium sibiricum* L. | La-gang | Geraniaceae | Herb | Whole plant | Pneumonia, lung heat (Jia and Zhang, 2016) |
| 425 | *Geranium wallichianum* D. Don ex Sweet | Ge-xian-sheng-bai | Geraniaceae | Herb | Root | Swelling of the lungs (Jia and Zhang, 2016) |
| 426 | *Erodium stephanianum* Willd. | Bo-er-qiong | Geraniaceae | Herb | Dried aboveground parts | Lung disease (Zhong and Song, 2020) |
| 427 | *Geranium wilfordii* Maxim. | Bo-er-qiong | Geraniaceae | Herb | Dried aboveground parts | Lung disease (Zhong and Song, 2020) |
| 428 | *Geranium carolinianum* L. | Bo-er-qiong | Geraniaceae | Herb | Dried aboveground parts | Lung disease (Zhong and Song, 2020) |
| 429 | *Geranium nepalens*e Sweet | Bo-er-qiong | Geraniaceae | Herb | Dried aboveground parts | Lung disease (Zhong and Song, 2020) |
| 430 | *Geranium thunbergii*Siebold ex Lindley et Paxton | Bo-er-qiong | Geraniaceae | Herb | Dried aboveground parts | Lung disease (Zhong and Song, 2020) |
| 431 | *Gymnadenia conopsea*(L.)R. Br. | Wang-la | Orchidaceae | Herb | Dried tuber | pulmonary cough and wheeze (Control, 1996) |
| 432 | *Gymnadenia orchidis* Lindl. | Wang-la | Orchidaceae | Herb | Dried tuber | pulmonary cough and wheeze (Zhong and Song, 2020) |
| 433 | *Gymnadenia bicornis* T. Tang et K. Y. Lang | Wang-bao-la-ba | Orchidaceae | Herb | Tuber | Pulmonary cough and wheeze (Jia and Zhang, 2016) |
| 434 | *Gymnadenia crassinervis* Finet | Wang-bao-la-ba | Orchidaceae | Herb | Tuber | Pulmonary cough and wheeze (Jia and Zhang, 2016) |
| 435 | *Habenaria szechuanica* Schltr. | Wang-la-man-ba | Orchidaceae | Herb | Root | Pulmonary cough and wheeze (Jia and Zhang, 2016) |
| 436 | *Habenaria tibetica*  Schltr. ex Limpricht | Wang-la-man-ba | Orchidaceae | Herb | Root | Pulmonary cough and wheeze (Jia and Zhang, 2016) |
| 437 | *Herminium alaschanicum* Maxim. | Wang-la-man-ba | Orchidaceae | Herb | Tuber | Pulmonary cough and wheeze (Jia and Zhang, 2016) |
| 438 | *Herminium chloranthum* Tang et F. T. Wang | Wang-la-man-ba | Orchidaceae | Herb | Tuber | Pulmonary cough and wheeze (Jia and Zhang, 2016) |
| 439 | *Herminium monorchis* (L.) R. Br. | Wang-la-man-ba | Orchidaceae | Herb | Tuber | Pulmonary cough and wheeze, pulmonary trauma, pneumococcosis (Jia and Zhang, 2016) |
| 440 | *Bletilla striata*(Thunb. ex Murray) Rchb. F. | Ba-duo-la | Orchidaceae | herb | Whole plant | Hemoptysis in tuberculosis (Jia and Zhang, 2016) |
| 441 | *Dactylorhiza viridis*(Linnaeus) R. M. Bateman, Pridgeon & M. W. Chase | Wang-la | Orchidaceae | herb | Root | Pneumococcosis (Jia and Zhang, 2016) |
| 442 | *Epipactis helleborine* (L.) Crantz. |  | Orchidaceae | herb | Root | Cough with lung heat (Jia and Zhang, 2016) |
| 443 | *Satyrium yunnanense* Rolfe. | Wang-la-ga-bao | Orchidaceae | Herb | Tuber | Xeropulmonary cough (Jia and Zhang, 2016) |
| 444 | *Holboellia angustifolia* Wallich | Le-zhe | Lardizabalaceae | Wine | Stem | Lung disease (Jia and Zhang, 2016) |
| 445 | *Incarvillea delavayi* Bur. et Franch. | Wu-qu-ma-bao | Bignoniaceae | Herb | Flower; seed; root; aboveground parts | ,pulmonary tuberculosis, pneumorrhagia (Jia and Zhang, 2016) |
| 446 | *Incarvillea forrestii* Fletcher | Wu-que-ma-bo | Bignoniaceae | Herb | Flower | Pulmonary tuberculosis, pneumonia; pneumorrhagia (Jia and Zhang, 2016) |
| 447 | *Incarvillea Iutea* Bur. et Franch. | Wu-que-ma-bo | Bignoniaceae | Herb | Flower | Pulmonary tuberculosis, pneumonia; pneumorrhagia (Jia and Zhang, 2016) |
| 448 | *Incarvillea younghusbandii* Sprague | Wu-que-ma-bo | Bignoniaceae | Herb | Whole plant | Pulmonary tuberculosis; pneumonia; pneumorrhagia (Jia and Zhang, 2016) |
| 449 | *Oroxylum indicum* (L.) Bentham ex Kurz | Zhan-ba-ga | Bignoniaceae | Herb | Seed | Pneumonia (Jia, 2005) |
| 450 | *Roscoea tibetica* Bat. | Wu-xiong-di | Zingiberaceae | Herb | Whole plant | Pulmonary abscess (Jia and Zhang, 2016) |
| 451 | *Alpinia officinarum* Hance | Ga-ma | Zingiberaceae | Herb | Rhizome | Pulmonary abscess (Jia and Zhang, 2016) |
| 452 | *Alpinia galanga* (L.) Willd. | Su-mai | Zingiberaceae | Herb | Rhizome | Pulmonary abscess (Jia and Zhang, 2016) |
| 453 | Zingiber officinale Roscoe | Ga-jia | Zingiberaceae | Herb | Rhizome | Lung disease (Jia and Zhang, 2016) |
| 454 | *Kaempferia galanga* L. | - | Zingiberaceae | Herb | Rhizome | Pulmonary abscess (Jia, 2005) |
| 455 | *Lagenaria siceraria* (Molina) Standl. | Ga-bei-zhe-bu | Cucurbitaceae | Herb | Dried and ripe fruit or seed | Lung disease (Control, 1996) |
| 456 | *Lagenaria siceraria* Standl. var. *microcarpa* (Naud.) | Ga-bei | Cucurbitaceae | Herb | Dried and ripe fruit or seed | Lung disease (Jia and Zhang, 2016) |
| 457 | *Lepisorus bicolor* Ching | Cha-bei | Polypodiaceae | Herb | Whole plant | Cough with lung heat (Jia, 2005) |
| 458 | *Lepisorus clathratus*  (C. B. Clarke) Ching | Zha-bei | Polypodiaceae | Herb | Whole plant | Cough with lung hea (Luo, 1997) |
| 459 | *Lepisorus contiryus* (Christ) Ching | Cha-bei | Polypodiaceae | Herb | Whole plant | Cough with lung heat (Yang, 1987) |
| 460 | *Lepisorus morrisonensis* (Hayata) H. Ito | Cha-bei | Polypodiaceae | Herb | Whole plant | Cough with lung heat (Jia, 2005) |
| 461 | *Lepisorus pseudonudus* Ching | Cha-bei | Polypodiaceae | Herb | Whole plant | Cough with lung heat (Jia and Zhang, 2016) |
| 462 | *Pyrrosia gralla* (Gies.) Ching | Cha-bei | Polypodiaceae | Herb | Whole plant | Cough with lung heat (Jia, 2005) |
| 463 | *Pyrrosia heteractis* (Mett. ex. Kuhn) Ching | Cha-bei-zheng-wa | Polypodiaceae | Herb | Whole plant | Cough with lung heat (Jia and Zhang, 2016) |
| 464 | *Pyrrosia mollis* (C. Presl) Hovenk. | Cha-bei-zheng-wa | Polypodiaceae | Herb | Whole plant | Cough with lung heat (Jia and Zhang, 2016) |
| 465 | *Pyrrosia stenophylla* (Bedd.)  Ching | Cha-bei-zheng-wa | Polypodiaceae | Herb | Whole plant | Cough with lung heat (Jia and Zhang, 2016) |
| 466 | *Pyrrosia lingua* (Thunb) Earwell | Zha-bei | Polypodiaceae | Herb | Dried leaf | Cough with lung heat (Jia, 2005) |
| 467 | *Pyrrosia petiolosa* (Christ) Ching | Zha-bei | Polypodiaceae | Herb | Dried leaf | Cough with lung heat (Zhong and Song, 2020) |
| 468 | *Pyrrosia sheareri* (Baker.) Ching | Zha-bei | Polypodiaceae | Herb | Dried leaf | Cough with lung heat (Jia, 2005) |
| 469 | *Pyrrosia drakeana* (Franch.) Ching | Zha-bei | Polypodiaceae | Herb | Dried leaf | Cough with lung heat (Jia, 2005) |
| 470 | *Pyrrosia calvata* (Baker) Ching | Zha-bei | Polypodiaceae | Herb | Dried leaf | Cough with lung heat (Luo, 1997) |
| 471 | *Ligustrum robustum* (Roxb.)Blume | Jia-xing-man-ba | Oleaceae | Tree | Fruit | Pulmonary tuberculosis (Jia and Zhang, 2016) |
| 472 | *Syringa reticulata*subsp.*amurensis* (Ruprecht) P. S. Green et M. C. Chang | Zhan-tan-ga-bao | Oleaceae | Tree | Trunkk; branche | Fei xu re (Jia and Zhang, 2016) |
| 473 | *Triosteum pinnatifidum* Maxim. | Da-ma-mai-duo | Caprifoliaceae | Herb | Fruit | Pulmonary dryness; pulmonary congestion (Jia and Zhang, 2016) |
| 474 | *Lonicera ligustrina*var.*pileata*(Oliv.) Franch. | Nu-xing-cha-ga | Caprifoliaceae | Vine | Fruit | Portopulmonary disease (Jia and Zhang, 2016) |
| 475 | *Lonicera rupicola* Hook.f.et Thoms. | Pang-ma | Caprifoliaceae | Vine | Fruit; seed; branche; leaf | pneumonia (Jia and Zhang, 2016) |
| 476 | *Lonicera webbiana* Wall. ex DC. | Pang-ma | Caprifoliaceae | Vine | Flower | Pneumonia (Jia and Zhang, 2016) |
| 477 | Lonicera tibetica Bur.et Franch. | Qi-xiang起象 | Caprifoliaceae | Vine | Fruit; seed; branche; leaf | Pneumonia(Ma, 2012) |
| 478 | *Morus serrata* Roxb. | Ta-er-xing | Moraceae | Tree | Leaf; bast of baranch | Tracheitis (Jia and Zhang, 2016) |
| 479 | *Myricaria davurica*(Willd.) Ehrenb. | Wen-bu | Tamarix chinensis | Shrub | Aboveground parts | Lung disease (Jia, 2005) |
| 480 | *Myricaria paniculata* P. Y. Zhang et Y. J. Zhang | Wong-bu | Tamarix chinensis | Shrub | Twig | Pneumonia; toxic fever in pneumonia (Jia and Zhang, 2016) |
| 481 | *Myricaria rosea* W. W. Sm. | Wang-bu | Tamarix chinensis | Shrub | Twig; leaf | Pneumonia; toxic fever in pneumonia (Jia, 2005) |
| 482 | 三春柳*Myricaria squamosa* Desv. | Wong-bu | Tamarix chinensis | Shrub | Twig; leaf | Pneumonia; toxic fever in pneumonia (Jia and Zhang, 2016) |
| 483 | *Polygala sibirica* L. | Qi-xiang-ga-mo | Polygalaceae | Herb | Whole plant | Tracheitis (Jia and Zhang, 2016) |
| 484 | *Polygala tenuifolia* Willd. | Qi-xiang-ga-mo | Polygalaceae | Herb | Whole plant | Tracheitis (Jia and Zhang, 2016) |
| 485 | *Populus alba* L. | Ma-geng | Salicaceae | Tree | Bark; teig;leaf | Lung abscess (Luo, 1997) |
| 486 | *Populus nigra*var. *italica*(Moench)Koehne | Ma-geng | Salicaceae | Tree | Bark | Lung abscess (Jia and Zhang, 2016) |
| 487 | *Salix cheilophila* Schneid. | Jiang-ma | Salicaceae | Tree | Stem; stem barksall; leaf | Lung abscess (Luo, 1997) |
| 488 | *Salix microstachya*var.*bordensi*s (Nakai) C.F.Fang | Jiang-ma | Salicaceae | Tree | Stem; root; stem barksall | Lung abscess (Jia and Zhang, 2016) |
| 489 | *Salix oritrepha* Scheid. | Jiang-ma | Salicaceae | Tree | Stem; root; stem barksall | Lung abscess (Jia and Zhang, 2016) |
| 490 | *Salix babylonica* L. | Jiang-ma | Salicaceae | Tree | Fresh or dried shoots | Lung abscess (Luo, 1997) |
| 491 | *Populus davidana* Dode | Ma-geng | Salicaceae | tree | Dried stem and branche | lung abscess (Luo, 1997) |
| 492 | *Populus simonii*Carr. |  | Salicaceae | tree | Dried stem and branche | Lung disease (Control, 1996) |
| 493 | *Populus adenopoda* Maxim. | Ma-geng | Salicaceae | tree | Dried stem and branche | Lung abscess (Jia, 2005) |
| 494 | *Populus rotundifolia* var. *duclouxiana* (Dode) Gomb. | Ma-ka | Salicaceae | Tree | Dried bark | Lung disease (Ma, 2012) |
| 495 | *Potamogeton pectinatus* (Linnaeus) Borner | Suo-dun-ba | Potamogetonaceae | Herb | Whole plant | Pneumonia (Jia and Zhang, 2016) |
| 496 | *Primula blinii* Levl. | Xiang-di-ga-bao | Primulaceae | Herb | Flower | Lung disease (Jia and Zhang, 2016) |
| 497 | *Primula crocifolia* Pax et Hoffm. | Xing-xing-zhe-wu | Primulaceae | Herb | Whole pant | Lung disease (Ma, 2012) |
| 498 | *Primula dryadifolia* Franch. | Xiang-xiang-zhe-wu | Primulaceae | Herb | Whole pant | Lung disease (Jia and Zhang, 2016) |
| 499 | *Primula orbicularis* Hemsl. | Xiang-zhi-sai-bao | Primulaceae | Herb | Flower | Lung disease (Jia and Zhang, 2016) |
| 500 | *Primula poissoni* Franch. | O-rui-mo-niang | Primulaceae | Herb | Whole pant | Cough with lung heat (Jia, 2005) |
| 501 | *Primula pulchella* Franch. | Xiang-zhi-en-bao | Primulaceae | Herb | Whole pant | Cough with lung heat (Jia and Zhang, 2016) |
| 502 | *Primula russeola* Balf. F. et Forr. |  | Primulaceae | Herb | Flower | Lung heat (Jia and Zhang, 2016) |
| 503 | *Primula serratifolia* Franch. | Xiang-zhi-ma-bao | Primulaceae | Herb | Flower | Lung disease (Jia, 2005) |
| 504 | *Primula sikkimensis* Hook. | Xiang-zhi-se-bao | Primulaceae | Herb | Flower | Lung heat (Jia and Zhang, 2016) |
| 505 | *Primula sonongii* Chen et C. M. Hu | Xiang-zhi-sai-bao | Primulaceae | Herb | Flower | Lung disease (Jia and Zhang, 2016) |
| 506 | *Primula stenocalyx* Maxim. | Xiang-zhi-mo-bao | Primulaceae | Herb | Flower | Lung abscess (Jia and Zhang, 2016) |
| 507 | *Primula szechuanica* Pax | Xiang-zhi-sai-bao | Primulaceae | Herb | Flower | Lung disease (Jia and Zhang, 2016) |
| 508 | *Primula tangutica* Duthie | Ke-e-da-mo | Primulaceae | Herb | Flower | Lung disease (Jia and Zhang, 2016) |
| 509 | *Primula tangutica* var. *flavescens* Chen et C.M.Hu | Xiang-zhi-sai-bao | Primulaceae | Herb | Flower | Lung disease (Jia and Zhang, 2016) |
| 510 | *Pyrola calliantha* H. Andr. | Qi-men-shi | Pyrolaceae | Herb | Whole plant | Pulmonary crests (Jia and Zhang, 2016) |
| 511 | *Rhodiola crenulata* (Hook. f. et Thoms.) H. Ohba | Suo-luo-ma-bao | Crassulaceae | Herb | Rhizome; root; flower | Lung heat, Pulmonary crests, pulmonary tuberculosis, pneumonia, tracheitis (Jia, 2005) |
| 512 | *Rhodiola atuntsuensis* (Praeg.)  S. H. Fu | Suo-luo-ma-bao | Crassulaceae | Herb | Rhizome; root | Tracheitis, (Jia and Zhang, 2016) |
| 513 | *Rhodiola bupleuroides* (Wall.ex Hook. F. et .Thoms.) S. H. Fu | Suo-luo-ma-bao | Crassulaceae | Herb | Rhizome; root | Tracheitis, (Jia and Zhang, 2016) |
| 514 | *Rhodiola coccinea* (Royle) Borissova | Suo-luo-ma-bao | Crassulaceae | Herb | Rhizome | Tracheitis, (Jia and Zhang, 2016) |
| 515 | *Rhodiola dumulosa* (Franch.) S. H. Fu | Can-qiong-wa | Crassulaceae | Herb | Root | Lung heat (Jia and Zhang, 2016) |
| 516 | *Rhodiola fastigiata*  (Hook. f. et Thoms.) S. H. Fu | Suo-luo-ma-bao | Crassulaceae | Herb | Whole plant | Tracheitis, cough with lung heat (Administration, 2020) |
| 517 | *Rhodiola kirilowii* (Regel) Maxim. | Ga-du-er | Crassulaceae | Herb | Whole plant | Pneumonia, lung heat (Luo, 1997) |
| 518 | *Rhodiola litwinowii* A. Bor. | Suo-luo-ma-bao | Crassulaceae | Herb | Rhizome | Pneumonia (Jia and Zhang, 2016) |
| 519 | *Rhodiola macrocarpa* (Praeg.)  S. H. Fu | Suo-luo-ma-bao | Crassulaceae | Herb | Rhizome | Pneumonia (Jia and Zhang, 2016) |
| 520 | *Rhodiola ovatisepala* (Hamet) S. H. Fu | Suo-luo-ma-bao | Crassulaceae | Herb | Rhizome | Pneumonia (Jia and Zhang, 2016) |
| 521 | *Rhodiola quadrifida*  (Pall.)  Fisch. et mey. | Suo-luo-ma-bao | Crassulaceae | Herb | Whole plant | Pneumonia, cough with lung heat (Jia and Zhang, 2016) |
| 522 | *Rhodiola sacra* (Prain ex Hamet)  S. H. Fu | Can-ga-er | Crassulaceae | Herb | Whole plant | Pneumonia; pulmonary tuberculosis, tracheitis, pneumonia (Jia, 2005) |
| 523 | *Sedum shigatsens*e Fröd. | Suo-luo-ma-bao | Crassulaceae | Herb | Rhizome; root | tracheitis (Jia and Zhang, 2016) |
| 524 | *Rhodiola wallichiana*var.*cholaensis* (Praeg.) S.H.Fu | Suo-luo-ma-bao | Crassulaceae | Herb | Whole plant | Pneumonia (Jia, 2005) |
| 525 | *Rhodiola yunnanensis* (Franch.)  S. H.Fu | Suo-luo-ma-bao | Crassulaceae | Herb | Rhizome | Tracheitis (Jia, 2005) |
| 526 | Rhododendron aganniphum Balf. F. et K. Ward | Da-ma | Ericaceae | Shrub | Flower; leaf; fruit | Lung sbscess (Jia and Zhang, 2016) |
| 527 | *Rhododendron augustinii* Hemsl. | Mei-duo-ma-er-bu | Ericaceae | Shrub | Flower | Tracheitis (Jia and Zhang, 2016) |
| 528 | *Rhododendron bulu* Hutch. | Ta-le-na-bao | Ericaceae | Shrub | Flower; leaf; twig | Abscess of lung (Jia and Zhang, 2016) |
| 529 | *Rhododendron coryanum* Tagg et Forrest | Da-ma | Ericaceae | Shrub | Flower; leaf | Lung abscess, tracheitis (Jia and Zhang, 2016) |
| 530 | *Rhododendron decorum* Franch. | Da-ma | Ericaceae | Shrub | Flower; leaf; fruit | Lung abscess (Jia and Zhang, 2016) |
| 531 | *Rhododendron flavidum* Franch. | Da-le | Ericaceae | Shrub | Flower; leaf; twig | Lung abscess, emphysema, tracheitis (Jia and Zhang, 2016) |
| 532 | *Rhododendron hypenanthum* Balf.f. | Ta-li-ga-bao | Ericaceae | Shrub | Leaf | Lung disease (Ma, 2012) |
| 533 | *Rhododendron intricatum* Franch. | Da-le | Ericaceae | Shrub | Flower; leaf; twig | Abscess of lung (Ma, 2012) |
| 534 | *Rhododendron lapponicum* (L.) Wahl. |  | Ericaceae | Shrub | Flower; leaf | Abscess of lung (Jia and Zhang, 2016) |
| 535 | *Rhododendron laudandum* Cowan | Ta-li-ga-bao | Ericaceae | Shrub | Leaf | Tracheitis (Jia and Zhang, 2016) |
| 536 | *Rhododendron lutescens* Franch. | Da-le | Ericaceae | shrub | Leaf; root; flower | Lung disease (Jia and Zhang, 2016) |
| 537 | *Rhododendron mainlingense* S. H. Huang et R. C. Fang | Ta-li-ga-bao | Ericaceae | Shrub | Leaf | Tracheitis (Jia and Zhang, 2016) |
| 538 | *Rhododendron nyingchiense* R. C. Fang et S. H. Huang | Ta-li-ga-bao | Ericaceae | Shrub | Leaf | Tracheitis (Jia and Zhang, 2016) |
| 539 | *Rhododendron phaeochrysum*var.*agglutinatum*(Balf.f.et Forrest) Chamb. ex Cullen et Chamb | Ta-li-ga-bao | Ericaceae | Shrub | Leaf | Tracheitis, lung abscess (Jia and Zhang, 2016) |
| 540 | *Rhododendron pingianum* Fang | Da-ma | Ericaceae | Shrub | Leaf; flower; seed | Lung abscess (Jia and Zhang, 2016) |
| 541 | *Rhododendron rufescens* Franch. | Da-le | Ericaceae | Shrub | Leaf; flower; branch | Emphysema, abscess of lung and tracheitis (Jia and Zhang, 2016) |
| 542 | *Rhododendron temenium* Balf. F. et Forrest | Ta-li-ga-bao | Ericaceae | Shrub | Leaf | Tracheitis (Jia and Zhang, 2016) |
| 543 | *Rhododendron thymifolium* Maxim | Da-le | Ericaceae | Shrub | Leaf; flower | Lung abscess (Jia, 2005) |
| 544 | *Rhododendron triflorum* Hook. f. | Da-ma | Ericaceae | Shrub | Leaf; flower | Lung abscess and tracheitis (Jia and Zhang, 2016) |
| 545 | *Rhododendron tubulosum* Ching ex W. Y. Wang | Ta-li-mo-bao | Ericaceae | Shrub | Leaf; flower | lung abscess and tracheitis (Jia and Zhang, 2016) |
| 546 | *Rhododendron vernicosum* Franch. | Da-ma | Ericaceae | Shrub | Leaf; flower; fruit | Lung abscess (Jia and Zhang, 2016) |
| 547 | *Rhododendron violaceum* Rehd. et Wils. | Da-le | Ericaceae | Shrub | Leaf; flower; fruit | Lung abscess (Jia and Zhang, 2016) |
| 548 | *Rhododendron dabanshanese* Fang et Wang | Da-ma | Ericaceae | Shrub | Dried flower | Lung abscess, tracheitis (Luo, 1997) |
| 549 | *Rhododendron przewalskii* Maxim | Da-ma | Ericaceae | Shrub | Dried flower | Lung abscess, tracheitis and (Jia, 2005) |
| 550 | *Rhododendron simsii* Planch. | Da-ma | Ericaceae | Shrub | Dried flower | Lung abscess, tracheitis and (Zhong and Song, 2020) |
| 551 | *Rhododendron anthopogonoides* Maxim. | Da-li | Ericaceae | Shrub | Dried flower; leaf | Tracheitis, emphysema (Jia, 2005) |
| 552 | *Rhododendron cephalanthum* Franch. | Da-li | Ericaceae | Shrub | Dried flower; leaf | Tracheitis and emphysema (Ma, 2012) |
| 553 | *Rhododendron anthopogon* D. Don | Ta-le-ga-bao | Ericaceae | Shrub | Flower; leaf; twig | Emphysema (Editorial Committee of Tibetan Medicine Journal, 1991) |
| 554 | *Santalum album* L. | Zan-dan-ga-bao | Santalaceae | Tree | Heartwood | Lung heat, pneumonia and lung abscess (Jia, 2005) |
| 555 | *Thesium chinense* Turcz. | Ao-chu-se-bu | Santalaceae | Herb | Whole plant | Lung heat, lung abscess (Jia, 2005) |
| 556 | *Thesium longiflorum* Hand. -Mazz. | Ao-chu-se-bu | Santalaceae | Herb | Whole plant | Lung heat, lung abscess (Luo, 1997) |
| 557 | *Thesium longiflorumTurcz.* | Ao-chu-se-bu | Santalaceae | Herb | Whole plant | Lung heat, lung abscess (Ma, 2012) |
| 558 | *Thesium ramosoides* Hendry. | Ao-chu-se-bu | Santalaceae | Herb | Whole plant | Lung heat, lung abscess (Jia and Zhang, 2016) |
| 559 | *Skimmia multinervia* Huang | Xie-kan | Rutaceae | Herb | Leaf | Lung heat (Jia and Zhang, 2016) |
| 560 | *Tinospora capillipes* (Oliv.) Gagnep. | Le-zhe | Menispermaceae | Vine | Rattan | Pneumonia (Jia and Zhang, 2016) |
| 561 | *Tinospora sagittata*  (Oliv.)  Gagnep. | Le-zhe | Menispermaceae | Vine | Rattan | Pneumonia (Ma, 2012) |
| 562 | *Tinospora cordifolia* Miers | Le-zhe | Menispermaceae | Vine | Rattan | Lung disease (Jia, 2005) |
| 563 | *Tinospora sinensis*Merr. | Le-zhe | Menispermaceae | Vine | Rattan | Lung disease (Jia, 2005) |
| 564 | *Valeriana amurensis* Smir. ex Komarov | Jia-bie | Valerianaceae | Herb | Whole plant | Lung abscess, pulmonary crest abscess (Jia and Zhang, 2016) |
| 565 | *Valeriana fauriei* Briq. | Zhi-ma-er | Valerianaceae | Herb | Whole plant | Lung abscess; pulmonary crest abscess (Jia and Zhang, 2016) |
| 566 | *Valeriana hardwickii* Wall. | Jia-bie | Valerianaceae | Herb | Whole plant | Lung abscess; pulmonary crest abscess (Jia and Zhang, 2016) |
| 567 | 缬草*Valeriana officinalis* L. | Zhi-ma-er | Valerianaceae | Herb | Whole plant | Lung abscess, pulmonary crest abscess (Jia, 2005) |
| 568 | *Vitis bryoniifolia*Bunge | Gun-zhu-mu | Vitaceae | Vine | Fruit | Pneumonia, pulmonary tuberculosis, lung heat (Jia and Zhang, 2016) |
| 569 | *Vitis amurensis* Rupr. | Gun-zhu-mu | Vitaceae | Vine | Fruit | Neumonia, pulmonary tuberculosis, lung heat and (Jia and Zhang, 2016) |
| 570 | *Vitis flexuosa* Thunb. | Gun-zhu-mu | Vitaceae | Vine | Fruit | Neumonia, pulmonary tuberculosis, lung heat and (Jia and Zhang, 2016) |
| 571 | *Vitis heyneana* Roem. et Schult | Gun-zhu-mu | Vitaceae | Vine | Fruit | Neumonia, pulmonary tuberculosis, lung heat and (Jia and Zhang, 2016) |
| 572 | *Vitis vinifera* L. | Gun-zhu-mu | Vitaceae | Vine | Fruit | Neumonia, pulmonary tuberculosis, lung heat and (Jia, 2005) |
| 573 | *Daphne tangutica* Maxim. | Se-xiang-na-ma | Thymelaeaceae | Tree | Flower | Lung abscess (Jia, 2005) |
| 574 | *Crocus sativus* L. | Gou-ri-gou-mu | Iridaceae | Herb | Flower and style | Pneumonia (Ma, 2012) |
| 575 | *Botrypus lanuginosum* Wall. | Jia-qia-a-ma | Botrychiaceae | Herb | Whole plant | Cough with lung heat (Jia, 2005) |
| 576 | *Pteris actiniopteroides* Christ |  | Pteridaceae | Herb | Whole plant | Cough with lung heat (Jia and Zhang, 2016) |
| 577 | *Hippuris vulgaris* L. | Dan-bu-ga-la | Taxophyllaceae | Herb | Leaf and branch | Lung heat (Luo, 1997) |
| 578 | *Mycenastrum* corium(Guers. ) Desv | Zhe-mo-xia | Cladocosae |  | Subentity | Cough with lung heat (Jia, 2005) |
| 579 | *Auricularia auricula* (L. ex Hook)Underw. | Mo-ruo-na-bu | Auriculaceae |  | Subentity | Cough due to deficiency of the lung (Jia, 2005) |
| 580 | *Trametes cinnabarina* (Jacq.) Franeh | Xin-ge-xiao-meng | Polyporaceae |  | Subentity | Tracheitis (Jia and Zhang, 2016) |
| 581 | *Ganoderma leucocontextum* T.H.Li,W.Q.Deng,Dong M. Wang & H.P.Hu. | Xia-guo | Polyporaceae |  | Subentity | Cough due to deficiency of the lung (Administration, 2020) |
| 582 | *Tremella fuciformis* Berk. | Mo-ruo-ga-bu | Tremella |  | Subentity | Cough due to deficiency of the lung;dry pulmonaray cough;atrophic (Jia, 2005) |
| 583 | *Tremella mesenterica* Fries | Mo-ruo-ga-bu | Tremella |  | Subentity | Cough due to deficiency of the lung; pulmonary tuberculosis (Luo, 1997) |
| 584 | *Usnea longissima* Ach. |  | Usneaceae |  | Dried thallus | Lung heat (Zhong and Song, 2020) |
| 585 | *Usnea diffracta* Vain |  | Usneaceae |  | Dried thallus | Lung heat (Zhong and Song, 2020) |
| 586 | *Thambolia subuliformis* (Ehrh.) W Culb. | Sai-gu | Rhizaceae |  | Thallus | Pulmonary tuberculosis hot flashes and lung heat (Ma, 2012) |

**Reference**

Administration, S.P.D., 2020. Tibetan herbal medicine standard in Sichuan Province. Sichuan Science and Technology Press, Sichuan.

Bairuozana, 2016. Miaoyin Herbal. Qinghai People's Publishing Press, Qinghai.

Commission, C.P., 1995. Drug Standards of Tibetan Medicine. Ministry of Health of the People's Republic of China, Beijing.

Control, Q.I.f.D., 1996. Chinese Tibetan Medicine (vol. 1-3). Shanghai Science and Technology Press, Shanghai.

Dimaer, D.Z.P.C., 2012. Jing Zhu Materia Medica. Shanghai Science and Technology Press, Shanghai.

Editorial Committee of Tibetan Medicine Journal, N.P.I.o.B., Chinese Academy of Sciences, 1991. Tibetan Medicine Journal. Qinghai People’s Publishing Press, Qinghai.

Gema, R.Q.D.J., 2016. The Sea of Drug Names. Qinghai People's Publishing Press, Qinghai.

Health Bureau of Tibet, Q., Sichuan, Gansu, Yunnan, and Xinjiang 2019. Tibetan Medicine Standards(vol. 1-2). Qinghai People’s Publishing Press, Qinghai.

Herbalism, E.B.o.C., 2002. Chinese Herbalism for Tibetan Medicine. Shanghai Science and Technology Press, Shanghai.

Jia, M.R., 2005. Chinese Ethnic Medicine. China Medical Science and Technology Press, Beijing.

Jia, M.R., Zhang, Y., 2016. Dictionary of Chinese Ethnic Medicine. China Medical Science and Technology Press, Beijing.

Luo, D.S., 1997. Chinese Tibetan Materia Medica. Ethnic Publishing Press, Beijing.

Ma, S.l., 2012. Moon King Medicine Clinic. Shanghai Science and Technology Press, Shanghai.

Qianyutuo, Y.D.G.B., 2016. Yutuo Herbal. Qinghai People's Publishing Press, Qinghai

Wang, B.Q., 2004. National Tibetan Medicine Standard Complete Book(vol. 1-3). China Medical Electronic Audio and Video press, Beijing.

Wei, Y.S., Wa, Z.X.D., Li, Y., 2020. Leiwuqi County. Chinese and Tibetan Medicine Resources. China Textile Publishing House, Beijing.

Xiwacuo, 2016. Dumu Herbal. Qinghai People's Publishing Press, Qinghai

Yang, J.S., 1987. Diqing Tibetan Medicine. Nationalities Publishing Press of Yunnan, Yunnan.

Yutuo, Y.D.G.B., 2012. Medicine Four Continues. Shanghai Science and Technology Press, Shanghai.

Zhong, G.Y., Song, M.X., 2020. Ethnic medicine prescription herbs for prescription preparations. The People's Health Press, Beijing.
